# Supplementary material for: Structural basis for the assembly and quinone transport mechanisms of the dimeric photosynthetic RC–LH1 supercomplex
Source: Nat Commun. 2022 Apr 13;13:1977. doi: 10.1038/s41467-022-29563-3 (PMC9007983; doi:10.1038/s41467-022-29563-3)
Supplement: Supplementary file 1 — Supplementary Information [file 41467_2022_29563_MOESM1_ESM.pdf]

Supplementary Information

for

Cao et al., “Structural basis for the assembly and quinone transport mechanisms of the dimeric photosynthetic RC–LH1 supercomplex”

SUPPLEMENTARY METHODS

Parameters of the coarse-grained (CG) models of the ligands: The parameters of the coarse-grained (CG) models of bacteriochlorophyll *a* (BCL), bacteriopheophytin *a* (BPH), and tetrastearoyl cardiolipin (TSCL) were created by modifying those of chlorophyll A (CLA) [1], pheophytin A (PHA) [1], and tetraoleoyl cardiolipin (TOCL) [2], respectively. BCL and BPH have an acetyl group in place of the vinyl group of CLA and PHA. Therefore, the SP3 beads of CLA and PHA that represent the vinyl group were replaced by Na beads. The parameters of TSCL were created by replacing the five beads representing the unsaturated aliphatic tails of TOCL with the four beads representing saturated aliphatic tails, according to the parameters of other lipids [3] (Data S2). The atoms of spheroidene (SPO) were mapped to the CG beads as shown in Fig. S36. The bead types and the parameters are listed in Data S3. The bonded parameters of SPO were determined based on the comparison of the results of coarse-grained molecular dynamics (CGMD) simulations with those of all-atom molecular dynamics (AAMD) simulations performed for a SPO molecule in water. The system for the AAMD simulation was composed of an SPO molecule and 6,988 TIP3P water molecules. The OPLS-AA force field [4] was used for the all-atom (AA) model of SPO and its parameters were determined using the LigParGen server [4,5]. The AAMD simulations were performed for 50 ns with the following methods. The temperature was kept at 303.15 K using the Nose-Hoover method [6,7] with coupling constants of 1.0 ps. The pressure was kept at 1.0×10^5 Pa using the Parrinello-Rahman method [8] with coupling constants of 5.0 ps. Electrostatic interactions were calculated using the reaction-field method [9] with a cutoff of 1.4 nm. Van der Waals interactions were calculated with a modified Lennard-Jones potential that was shifted so that the potential value is zero at the cut-off distance of 1.4 nm. Bond lengths involving hydrogen atoms were constrained using the linear constraint solver (LINCS) algorithm [10,11] to allow a time step of 2 fs. The system for the CGMD simulation was composed of an SPO CG model and 1,900 CG water models. The CGMD simulation was performed for 5 ns with the same methods as described in the Methods section of the text. We confirmed that the distributions of the bond lengths, the angles, and the dihedrals of the CG model of SPO were agreed well with those of the corresponding values calculated from the AAMD simulation (Fig. S36). Validity of the mapping of the CG bead types was confirmed by calculating water-octanol partition coefficient based on the CGMD simulations. The SPO-water system was composed of an SPO CG model and 1,700 CG water models. The SPO-octanol system was composed of an SPO CG model, 50 CG water models, and 760 1-octanol CG models. For each system, the CGMD simulations were performed for 2 ns each in 21 states with the λ values of 0, 0.05, 0.1, 0.15, 0.2, 0.25, 0.3, 0.35, 0.4, 0.45, 0.5, 0.55, 0.6, 0.65, 0.7, 0.75, 0.8, 0.85, 0.9, 0.95, and 1. Free-energy differences between the neighboring states were calculated using the Bennett acceptance ratio method [12,13]. Solvation free energies in water (ΔG_{wat}) and in 1-octanol (ΔG_{oct}) were calculated as the sum of the free-energy differences obtained from the CGMD simulations performed for the SPO-water and the SPO-octanol systems, respectively. The calculated ΔG_{wat} and ΔG_{oct} values were −13.08 kJ mol^{-1} and −97.26 kJ mol^{-1}, respectively. The partition coefficient calculated from these values was 14.51, which was well agreed with the value predicted by XLOGP3 (14.7) [14].

SUPPLEMENTARY FIGURES

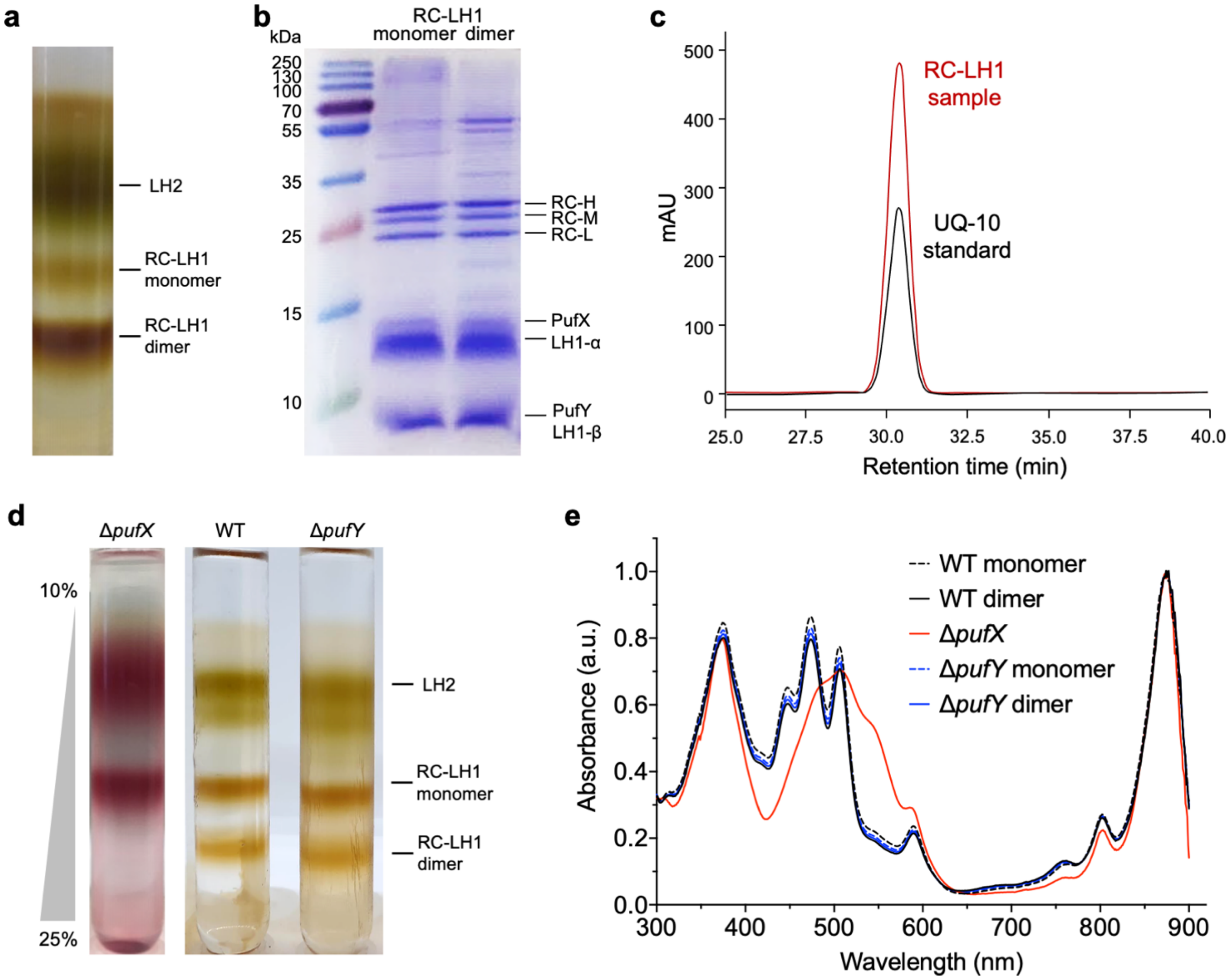

Fig. S1. Isolation and characterization of the *Rba. sphaeroides* RC−LH1 core complexes. (**a**) Separation of membrane proteins from WT *Rba. sphaeroides* using a 10-25 % continuous sucrose gradient. The pigmented fractions were identified as LH2, RC–LH1 monomer and RC–LH1 dimer from the top to the bottom. (**b**) SDS-PAGE of purified RC–LH1 monomers and dimers, stained by Coomassie Brilliant Blue. Analysis was repeated independently more than 3 times. Source data are provided as a Source Data file. (**c**) HPLC analysis of quinones in the RC-LH1 dimers (red) using UQ-10 as a standard (black), monitored at 275 nm. Analysis was repeated 3 times independently. (**d**) Separation of photosynthetic proteins from the WT, Δ*pufX*, and Δ*pufY Rba. sphaeroides* using 10-25 % continuous sucrose gradients. (**e**) Absorbance spectra of purified WT and mutant *Rba. sphaeroides* RC–LH1 complexes. Note that the Δ*pufX* strain was grown microoxically in the dark, given its inability to photosynthesize. The Δ*pufX* RC–LH1 complex has a broad absorption and maxima at 481, 509 and 545 nm, and thus a visibly different color, because of the conversion of the carotenoid spheroidene to spheroidenone under the microoxic culture conditions. Source data are provided as a Source Data file.

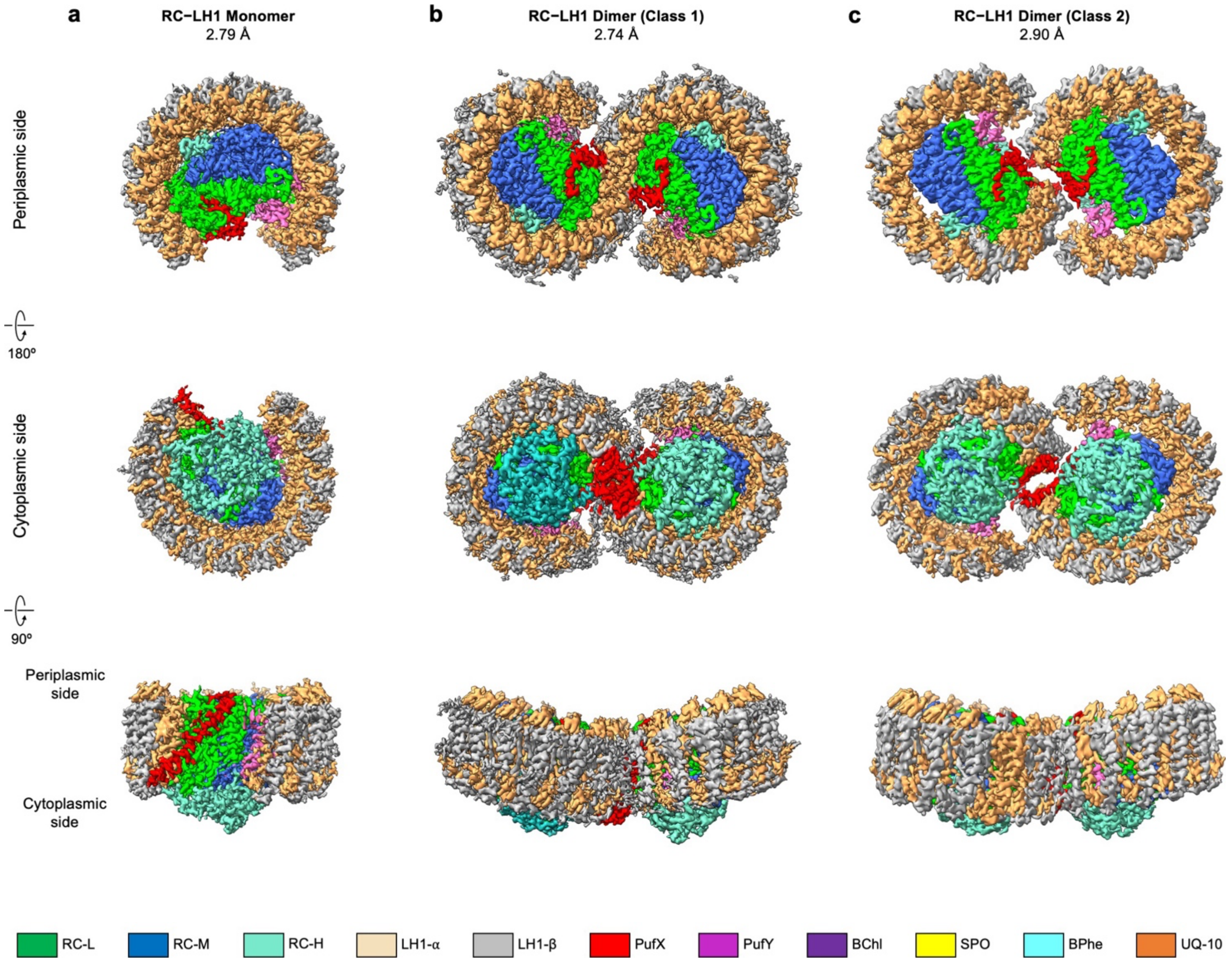

Fig. S2. Color-coded electron density maps of the RC–LH1 core complexes from *Rba. sphaeroides*. (**a**) The RC–LH1 monomer. (**b**) The Class-1 RC–LH1 dimer. (**c**) The Class-2 RC–LH1 dimer. Color scheme is presented as the same as shown in Fig. 1: LH1-α, wheat; LH1-β, grey; PufX, red; PufY, magenta; RC-L, green; RC-M, marine; RC-H, teal; bacteriochlorophylls (BChls), purple; bacteriopheophytins (BPhes), cyan; carotenoids, yellow; quinones, orange.

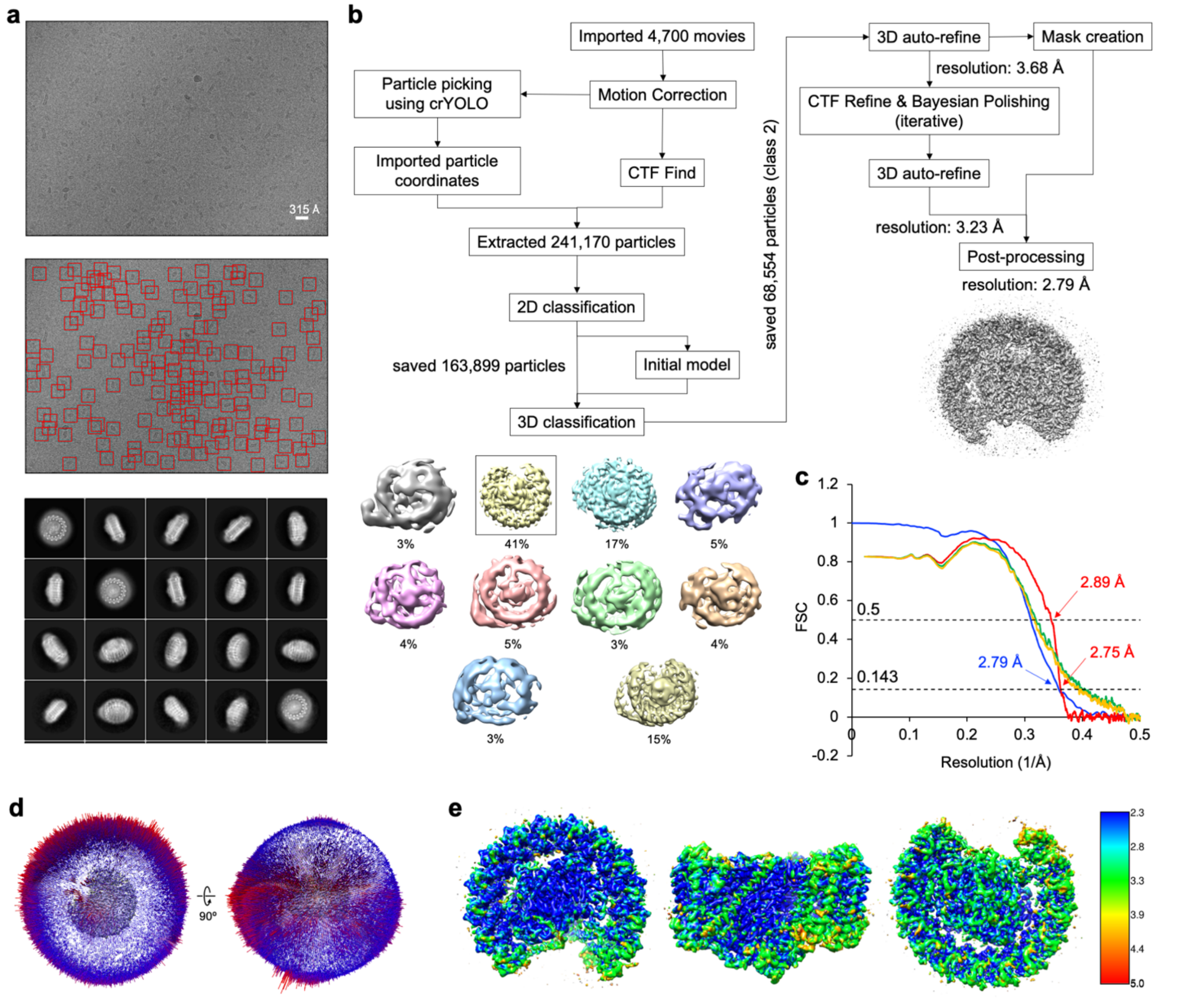

Fig. S3. Cryo-EM data process of the *Rba. sphaeroides* WT RC–LH1 monomer. (**a**) Motion-corrected example of a cryo-EM captured movie (4,700 movies in total) is shown on top and all particles picked from that micrograph are marked with red squares below. Imaged area is 610.6 nm × 433.7 nm. The red squares measure 318 × 318 Å. Representative reference-free 2D class averages are shown at the bottom. (**b**) Overview of cryo-EM data processing workflow for the WT RC–LH1 monomer dataset. Every major step is shown with the associated number of particles, percentage of particles per class, or estimated resolution. Selected 3D class that went into further processing is marked with a rectangle. (**c**) Fourier Shell Correlation (FSC) curves including FSC between two independently refined half-maps generated by RELION (blue) and model-to-map FSC generated by Phenix (red). Green line: FSC of the model refined against the 1st half map versus the 1st half map; Yellow line: FSC of the model refined against the 1st half map versus the 2nd half map. Global resolution values were calculated according to the gold-standard FSC = 0.5 and 0.143. Source data are provided as a Source Data file. (**d**) Angular distribution of the particles used to generate the final electron density map. The direction and length of each cylinder represent the view and the number of particles respectively. (**e**) Local resolution of the cryo-EM map as seen from the periplasmic view (left), side view (middle), and cytoplasmic view (right), estimated by RELION 3.1.

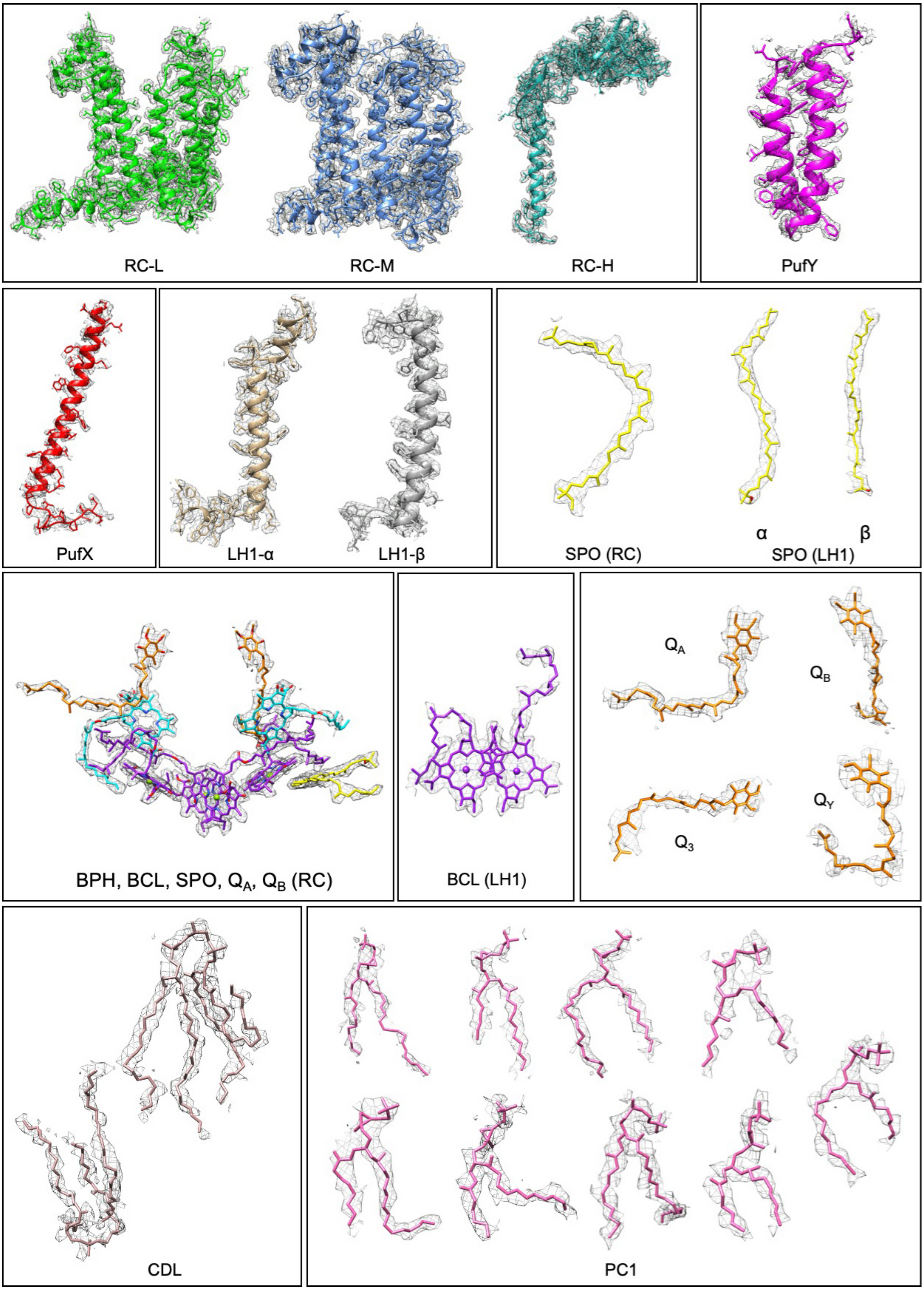

Fig. S4. Cryo-EM map densities and structural models of protein peptides and cofactors in the WT RC–LH1 monomer.

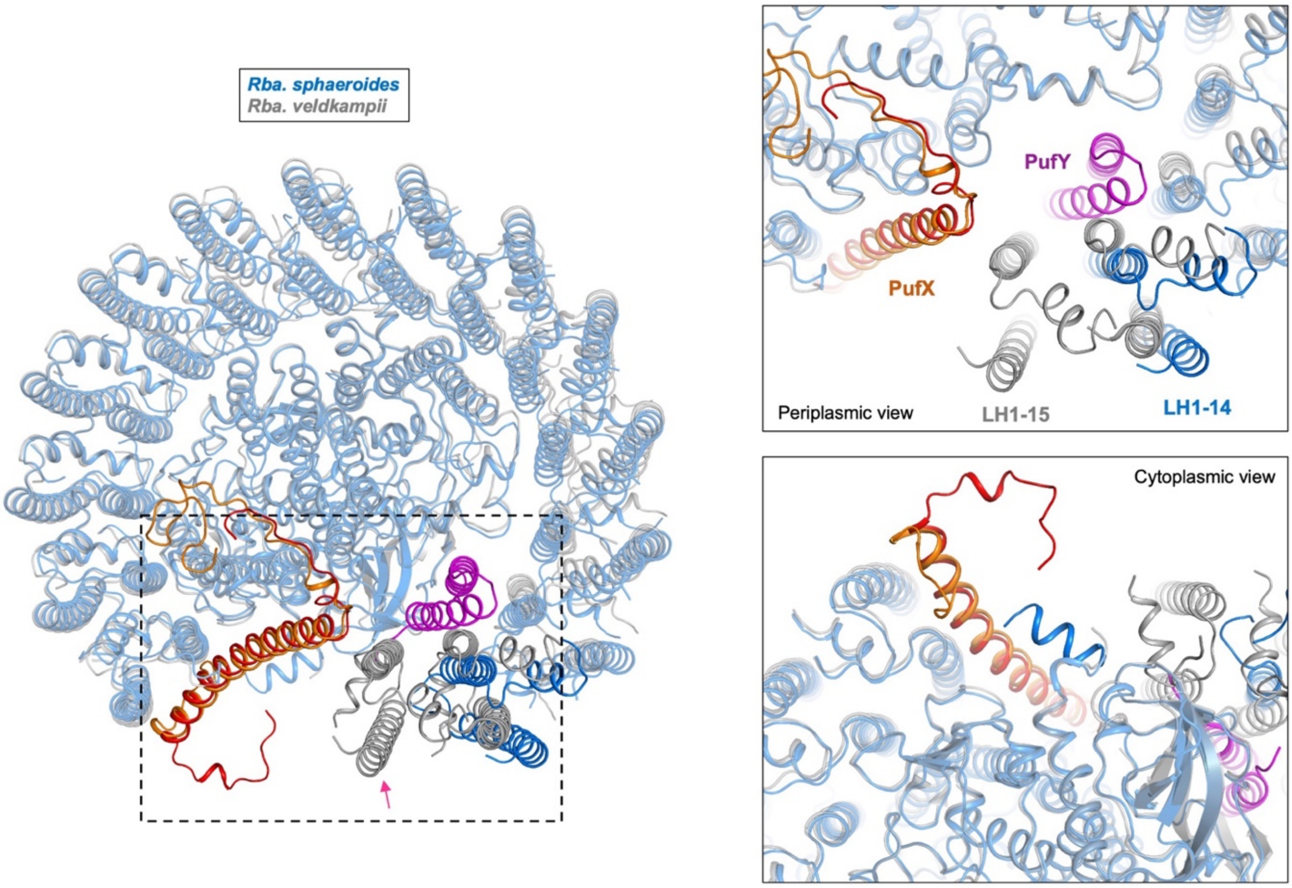

Fig. S5. Comparison of the RC–LH1 monomers from *Rba. sphaeroides* and *Rba. veldkampii* (PDB ID: 7DDQ [http://doi.org/10.2210/pdb7DDQ/pdb]**).** *Rba. sphaeroides* model is colored in blue, with PufX colored in red and PufY colored in magenta. *Rba. veldkampii* model is colored in grey, with PufX colored in orange. The 15th LH1 subunit (arrow) is present in the RC–LH1 monomer from *Rba. veldkampii* but is missing in *Rba. sphaeroides*. On the right, the top panel shows a close-up periplasmic view of the area around PufY at the periplasmic side; the bottom panel shows a focused cytoplasmic view of the PufX N-terminus at the cytoplasmic side.

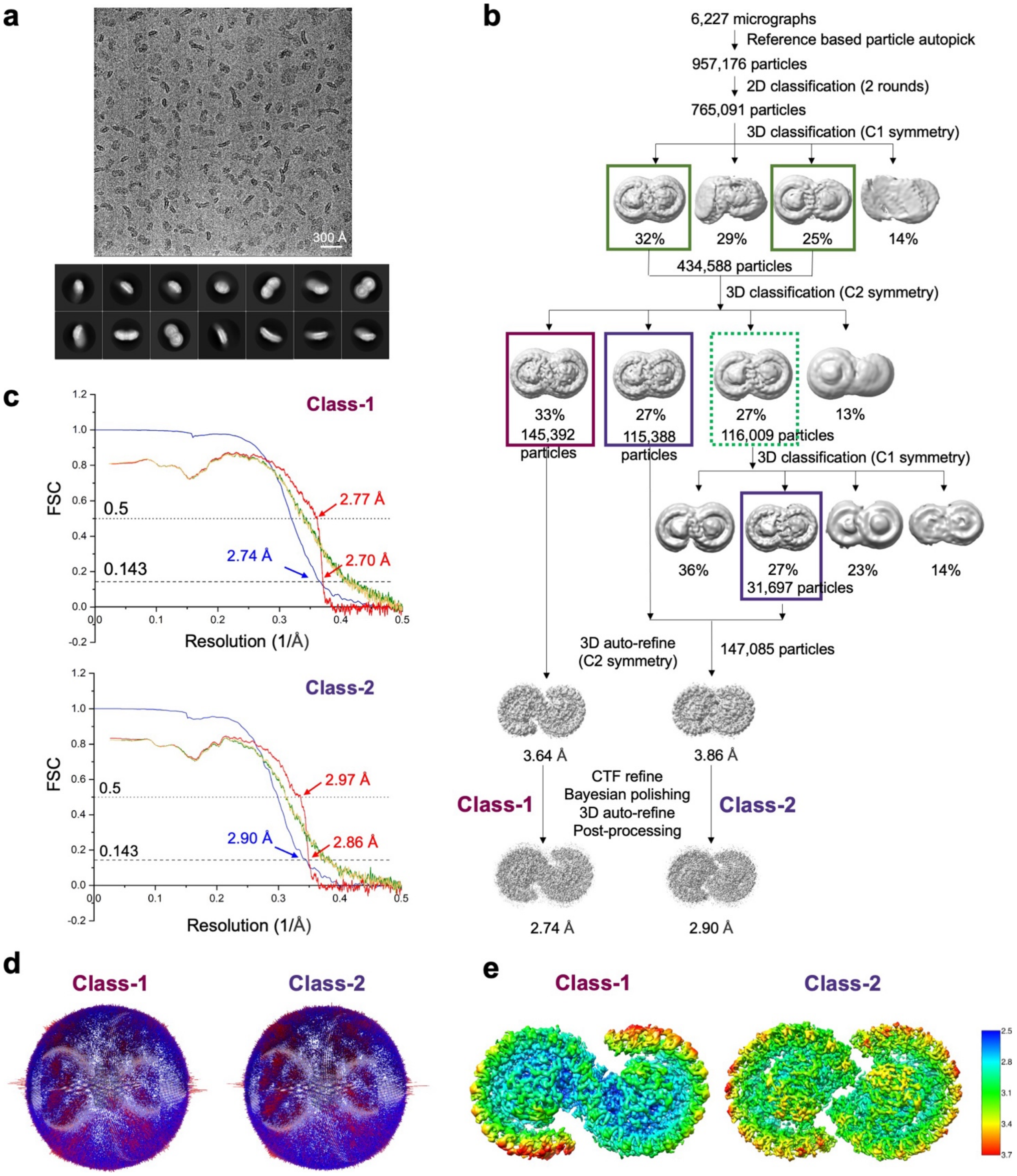

Fig. S6. Cryo-EM data process of the *Rba. sphaeroides* RC–LH1 dimers. (**a**) Representative cryo-EM micrograph (6,227 micrographs in total) of RC-LH1 dimeric supercomplex (upper) and representative 2D class averages (lower). The square size in 2D class average image is 416 Å × 416 Å. (**b**) Cryo-EM data processing workflow. (**c**) Gold-standard FSC curves of the Class-1 and Class-2 dimers, including FSC between two independently refined half-maps generated by RELION (blue) and model-to-map FSC generated by Phenix (red). Green line: FSC of the model refined against the 1st half map versus the 1st half map; Yellow line: FSC of the model refined against the 1st half map versus the 2nd half map. Global resolution values were calculated according to FSC = 0.5 and 0.143. Source data are provided as a Source Data file. (**d**) Angular distribution of Class-1 (left) and Class-2 (right) in the final 3D refinement with C2 symmetry. (**e**) Local resolution of Class-1 (left) and Class-2 (right) maps seen from the cytoplasmic view.

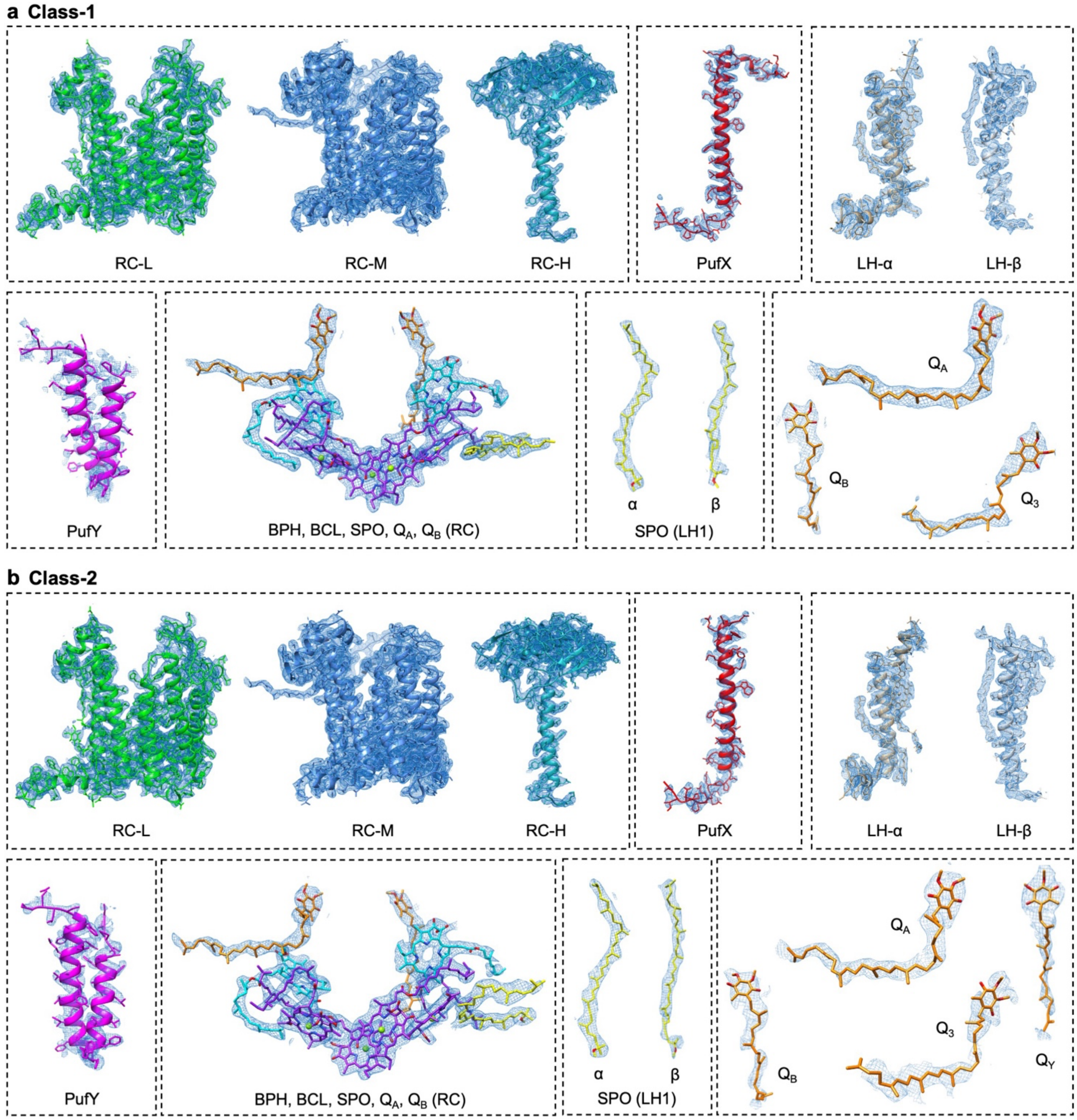

Fig. S7. Cryo-EM map densities and structural models of protein peptides and cofactors in the RC–LH1 dimers. (**a**) Cryo-EM densities of subunits and cofactors in Class-1. (**b**) Cryo-EM densities of subunits and cofactors in Class-2.

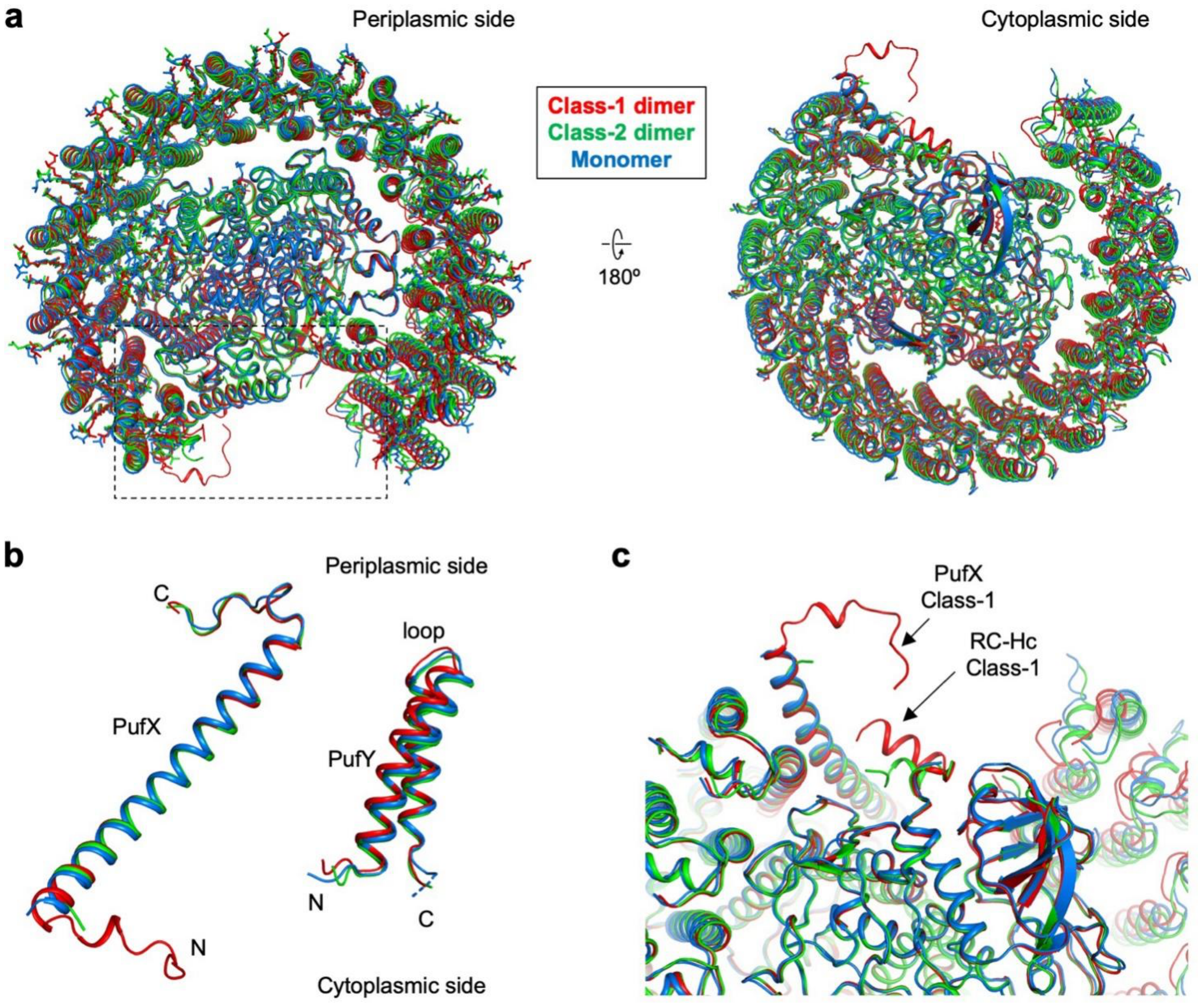

Fig. S8. Comparison of the monomeric structure of the WT Class-1 and Class-2 dimers and monomer superimposing on their RC subunits. (**a**) Superimposition of a monomer from the Class-1 dimer (red), Class-2 dimer (green), and RC−LH1 monomer (blue), as seen from the periplasmic side (left) and the cytoplasmic side (right). (**b**) Focused view of PufX and PufY in (**a**). (**c**) Focused view of PufX N-terminal region at the cytoplasmic side from the boxed region in (**a**). The extended tail of PufX and the C-terminus region of RC-H (RC-Hc) in the Class-1 dimer are marked with arrows.

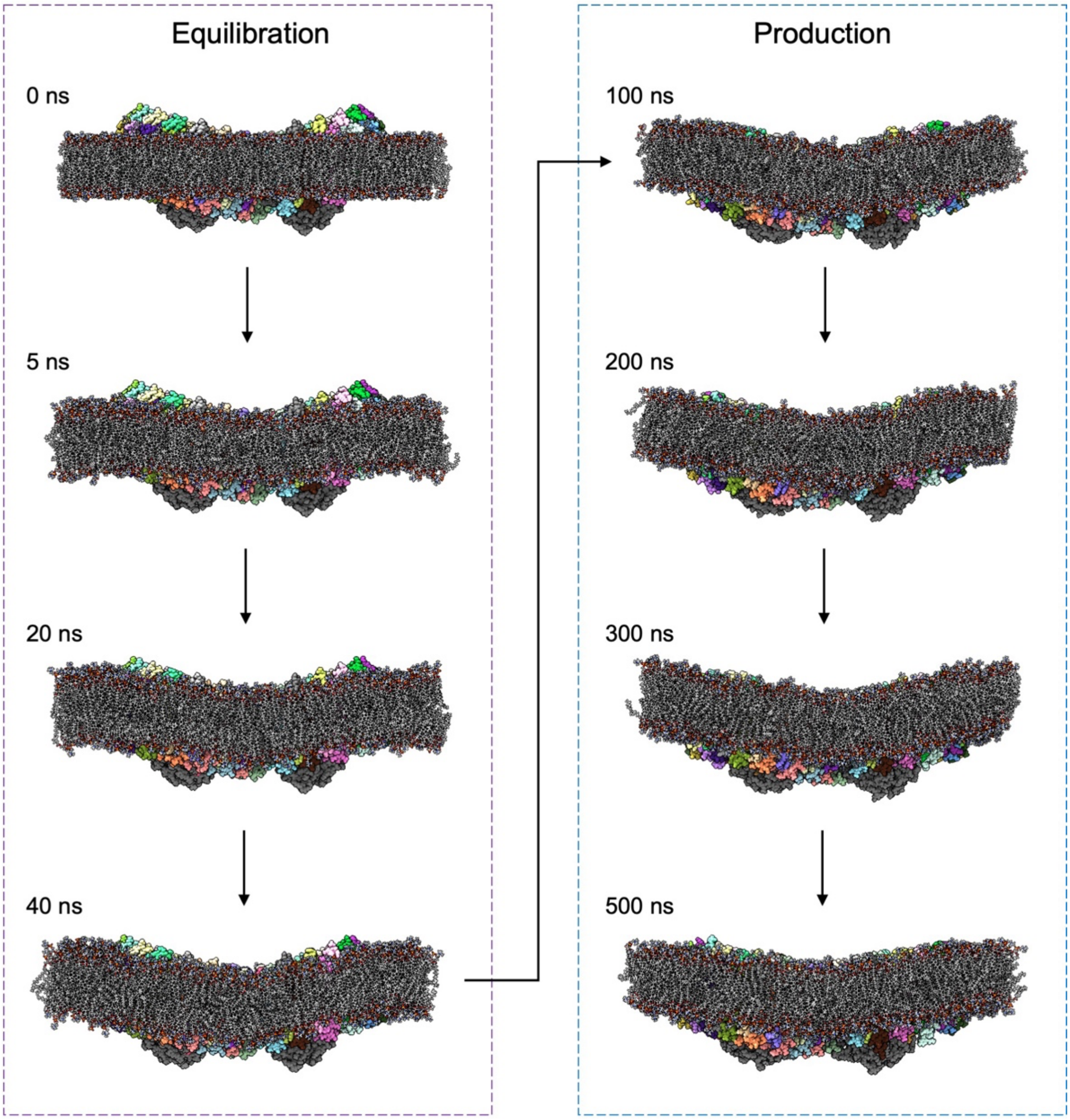

Fig. S9. Computational simulations of dimer-induced membrane curvature. Side views of the structures of the RC–LH1 dimer embedded in a lipid bilayer at 0, 20, and 40 ns of the equilibration and at 500 ns of the production all-atom molecular dynamics (AAMD) simulations are shown. Equilibration 0 ns to 40 ns represents the membrane curvature induced by the bent conformation of the RC–LH1 dimer (left). The curved structure of the lipid bilayer was maintained during the 500-ns unrestrained AAMD simulations. Protein chains are shown with molecular surfaces colored by chain. The atoms of ligands and lipids are shown in sticks. Hydrogen, carbon, nitrogen, oxygen, and phosphorous atoms are colored in white, gray, blue, red, and orange, respectively.

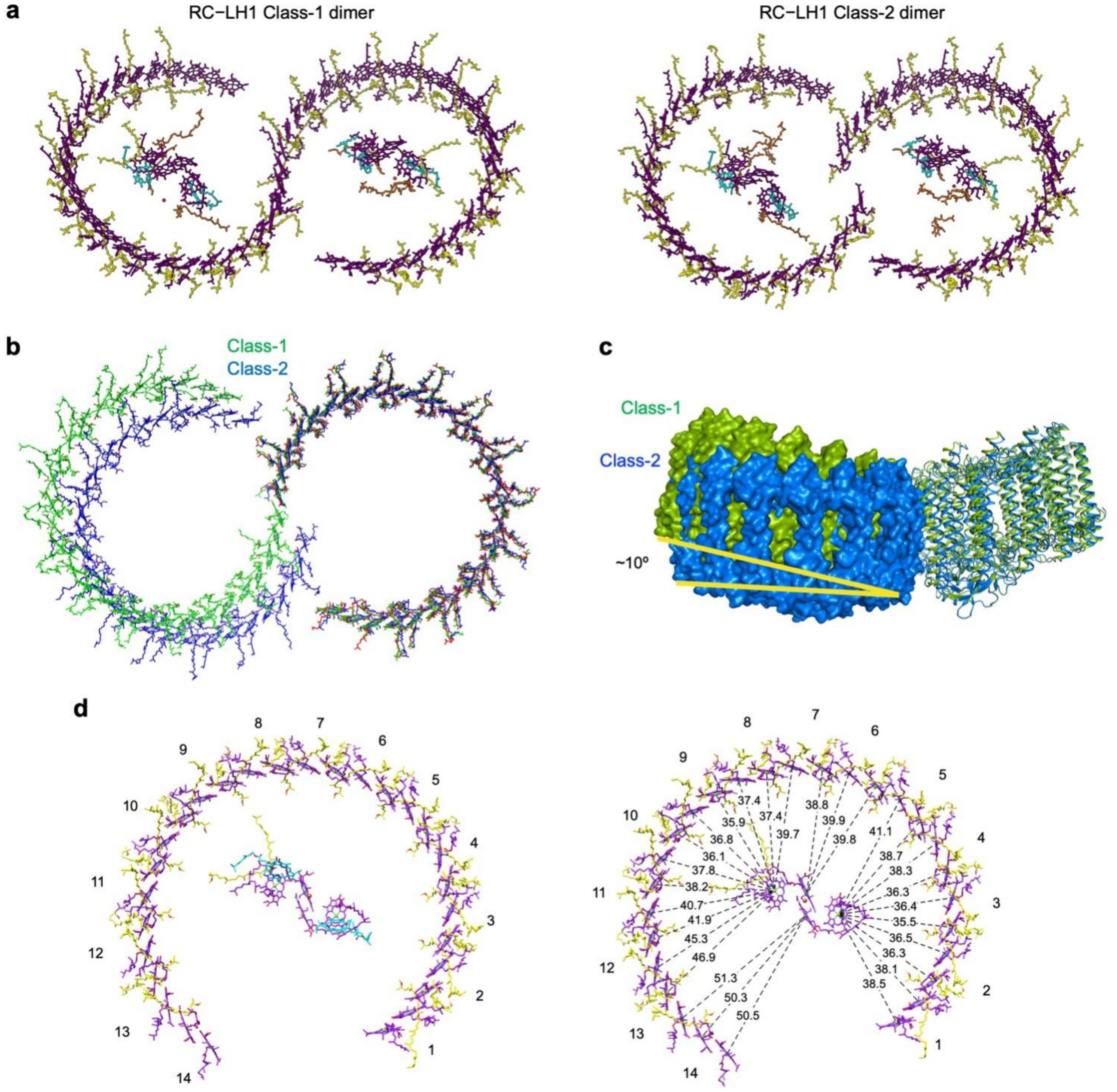

Fig. S10. Pigment arrangement and comparison of the two classes of the WT RC–LH1 dimers from *Rba. sphaeroides*. (**a**) Pigment arrangement within the WT RC–LH1 Class-1 dimer (left) and Class-2 dimer (right). Bacteriochlorophylls (BChls) are colored in purple, spheroidenes (SPOs) in yellow, bacteriopheophytins (BPhes) in cyan, and ubiquinone-10s (UQ-10s) in orange. (**b**) LH1 ring associated pigment comparison between Class-1 (green) and Class-2 (blue). Monomers on the right of each model were superimposed and the other monomer followed in its relation to the first. (**c**) Comparison of the tilted conformations of Class-1 (green) and Class-2 (blue). Monomers on the right of each model were superimposed and the other monomer followed in its relation to the first. (**d**) Pigment arrangement within the RC–LH1 monomer. The LH1 subunit numbers are depicted on the outside. On the right, the Mg-Mg distance of each LH1 BChl to the nearest RC BChl is shown in Å. BChls are colored in purple, SPOs in yellow, BPhes in cyan.

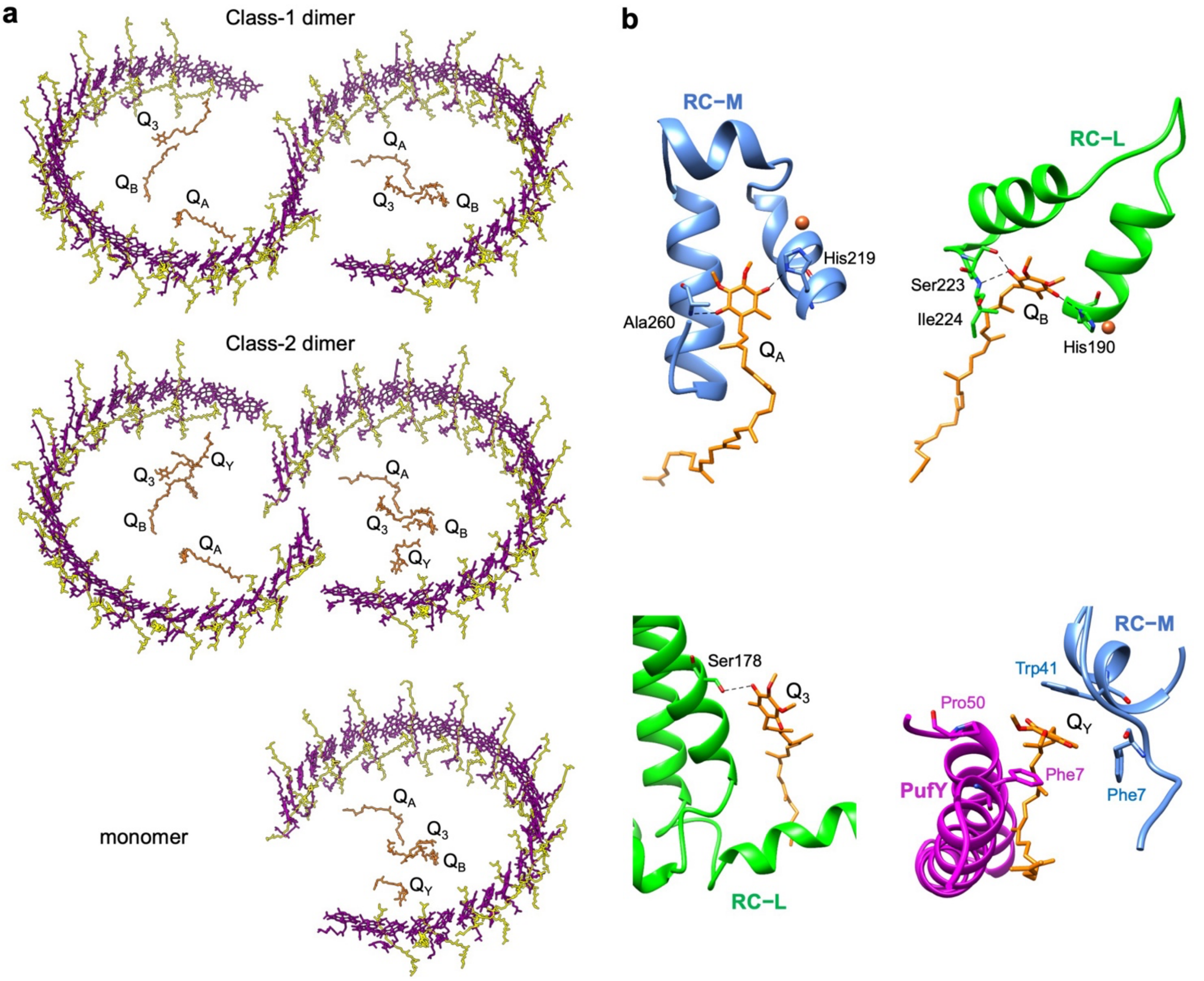

Fig. S11. Quinones in the *Rba. sphaeroides* RC–LH1 complexes. (**a**) Positions of identified quinone molecules in respect to the LH1 ring in the Class-1 dimer (top), the Class-2 dimer (middle), and the WT monomer (bottom). (**b**) Focused views of each identified quinone. The residues they interact with are marked and shown in sticks. The head group of Q_Y is surrounded by a specific pocket formed by the aromatic rings of the Trp41 and Phe7 residues of RC-M and the Phe7 and Pro50 residues of PufY.

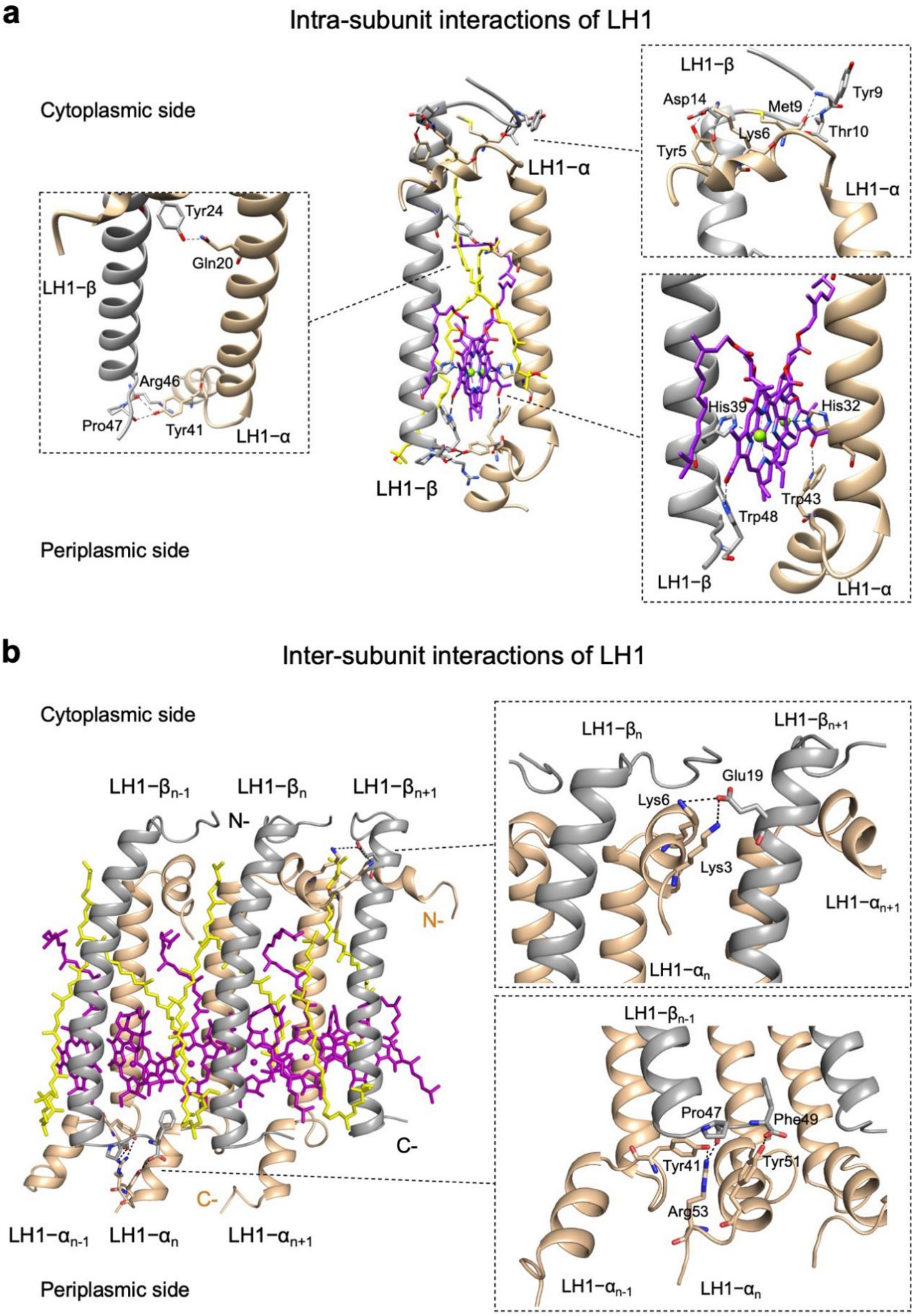

Fig. S12. Interactions within the LH1 subunit and between neighboring LH1 subunits. (**a**) Representative LH1 subunit organization. Associated pigments (BChl – purple, SPO – yellow) and interacting residues are shown in sticks. Close-up views of interactions within an LH1 subunit are shown in boxes on the side. (**b**) Representative LH1-LH1 interface. Associated pigments (BChl – purple, SPO – yellow) and interacting residues are shown in sticks. Close-up views of interactions between neighboring LH1 subunits are shown in boxes on the side. Interaction types and distances are displayed in Table S3.

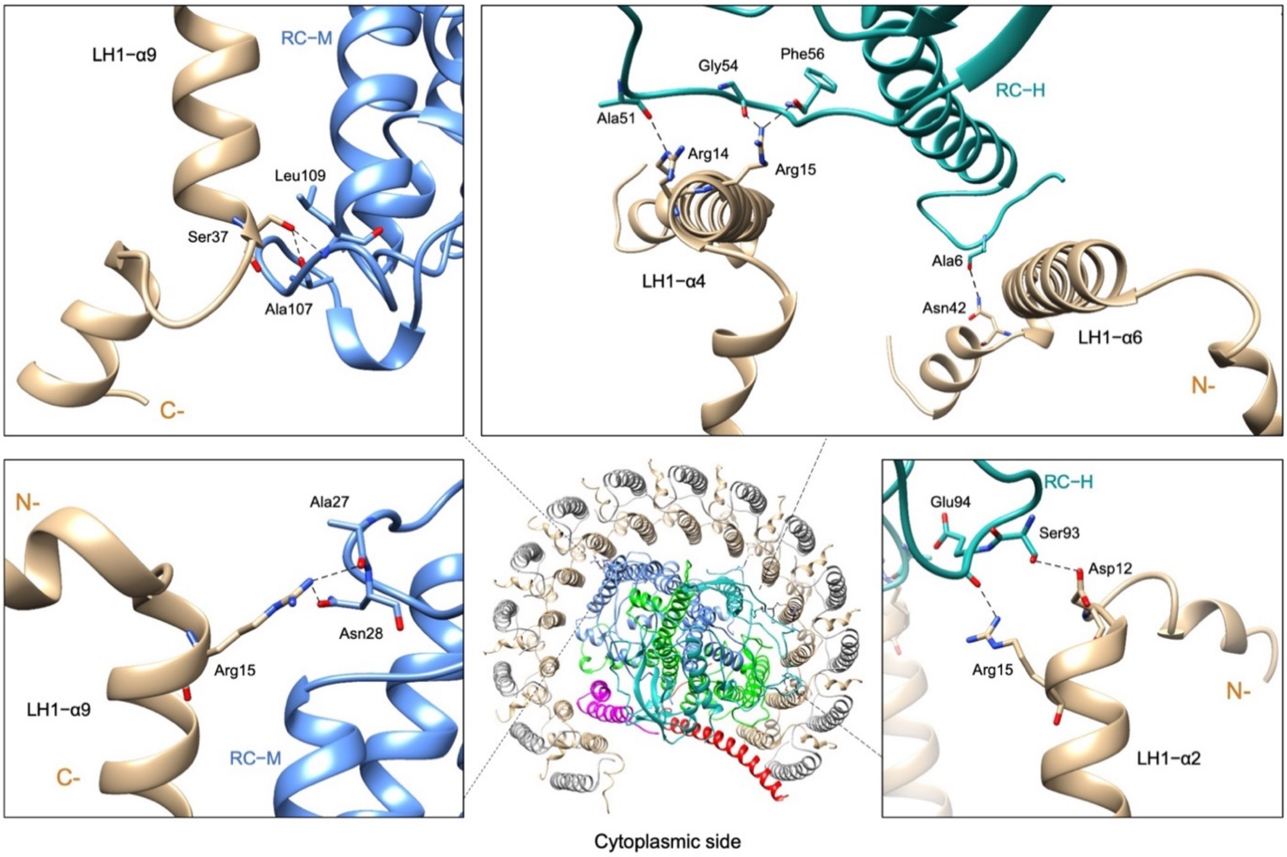

Fig. S13. Interactions between the RC and LH1 in the *Rba. sphaeroides* RC–LH1 complex. Interacting residues are shown in sticks. Detailed views of interactions are displayed in the boxes on the side while the middle view shows their general position within the complex. Types and distances of interactions between the RC and the LH1 are displayed in Table S3.

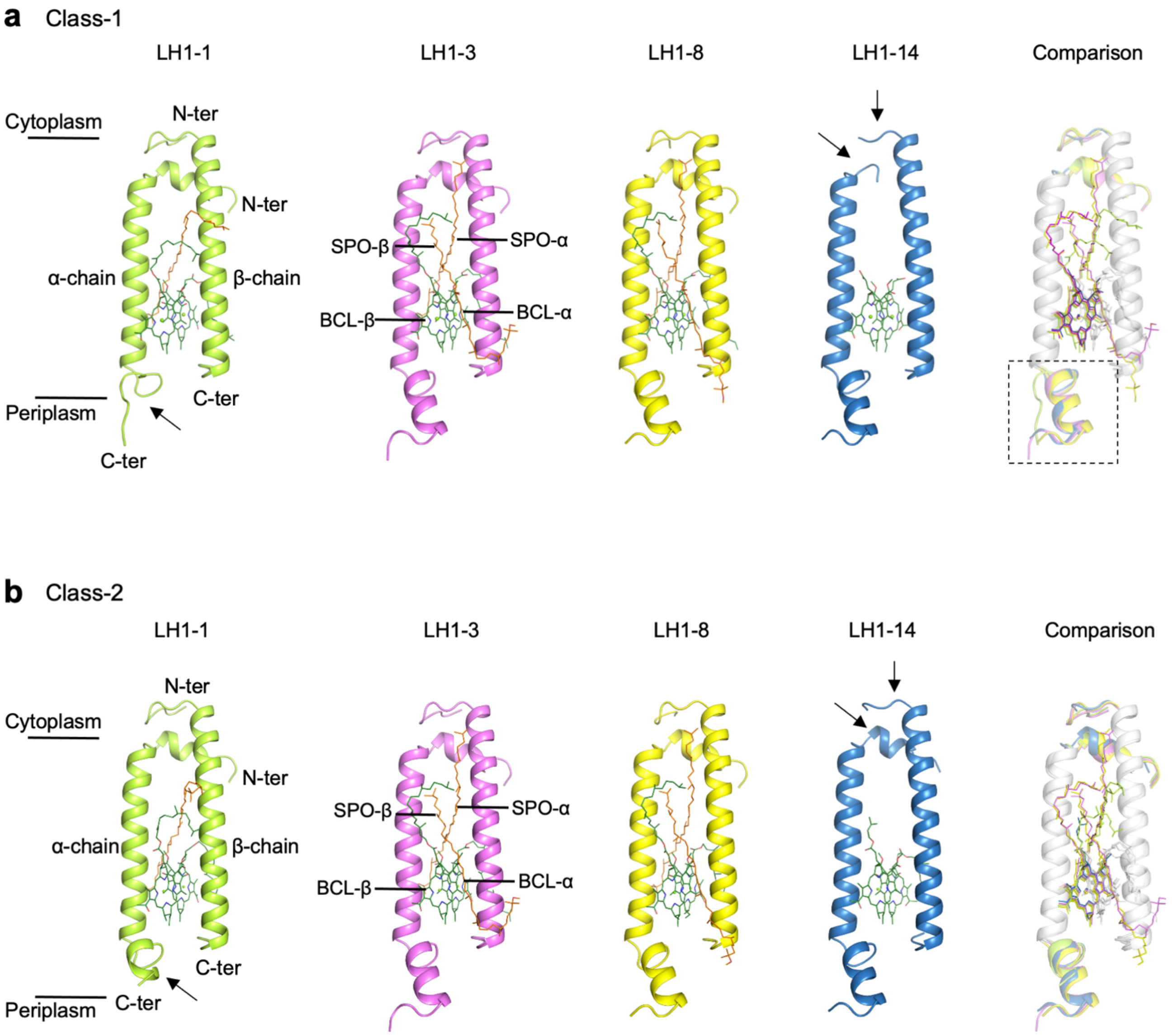

Fig. S14. Structural comparison of the 14 LH1 αβ-heterodimers of *Rba. sphaeroides*. (**a**) Overall structures and comparison of the LH1 subunits in the Class-1 dimer, including LH1-1 with 2 BChls (BChl-α and BChl-β) and 1 SPO-α, LH1-14 with only 2 BChls, as well as LH1-3 and LH1-8 with 2 BChls and 2 SPOs (SPO-α and SPO-β) representing other 12 similar LH1 heterodimers (LH1-2~LH1-13). The N-terminus and C-terminus are labeled in the LH1-1. SPO and BCL are labeled in the LH1-3. The C-terminus of LH1-1α and the N-termini of LH1-14α/β (arrows) exhibit distinct conformations compared with those of other LH1 polypeptides. The phytol tail of BCL-β and the SPO-α molecules in LH1-1 adopt different conformation than those in other LH1 subunits, enabling the association of PufX. Moreover, LH1-1 lacks SPO-β, which also facilitates its binding with PufX as otherwise the SPO molecule would clash with PufX. The SPO molecules in LH1-14 exhibit poor density, likely due to the terminal location and hence a higher mobility, and thus were not built in the structure. The tail regions of SPO-β molecules are highly flexible since they reach out into the solvent zone and adopt two major conformations as shown in LH1-3 and 1-8. Structural comparison of individual LH1 subunits is shown on the right. The similar part of LH1s is colored white and the different parts are highlighted in colors. (**b**) Structures and comparison of the LH1 subunits in the Class-2 dimer, including LH1-1 with 2 BChls and 1 SPO-α, LH1-14 with only 2 BChls, as well as LH1-3 and LH1-8 with 2 BChls and 2 SPOs representing other 12 similar LH1 heterodimers (LH1-2~LH1-13).

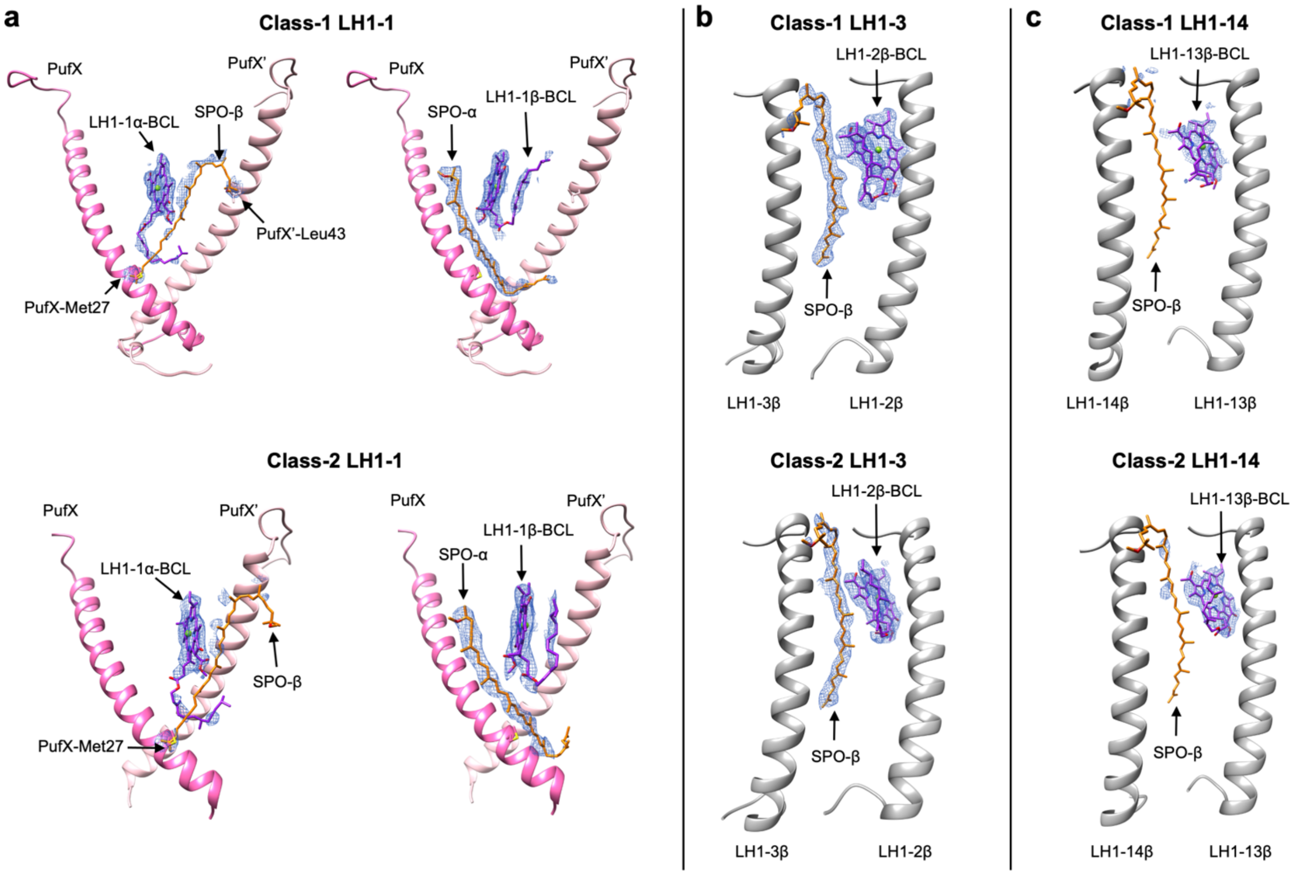

Fig. S15. Densities around the position of potential SPO-β molecule in LH1-1 and LH1-14, and the SPO-β density in LH1-3 in both Class-1 and Class-2 dimers. (**a**) Densities of the potential SPO-β and other cofactors in LH1-1 (shown in two panels for clarity). Left panel shows the densities of BChl of LH1-1α and the potential SPO-β molecule. Right panel shows the densities of BCL of LH1-1β and the SPO-α molecule. Compared with other cofactors, the potential SPO-β shows no density. In addition, the potential SPO-β molecule clashes with the phytol chain of BChl of LH1-1α, as well as Met27 of PufX and Leu43 of PufX', suggesting that LH1-1 does not possess SPO-β molecule. PufX and PufX' are shown as ribbons in pink and salmon, respectively. Residues are shown in sticks. (**b**) Densities of SPO-β of LH1-3 and BCL of LH1-2β. LH1-2β and LH1-3β are shown as ribbons. (**c**) Densities of potential SPO-β of LH1-14 and BCL of LH1-13β. LH1-13β and LH1-14β are shown as ribbons.

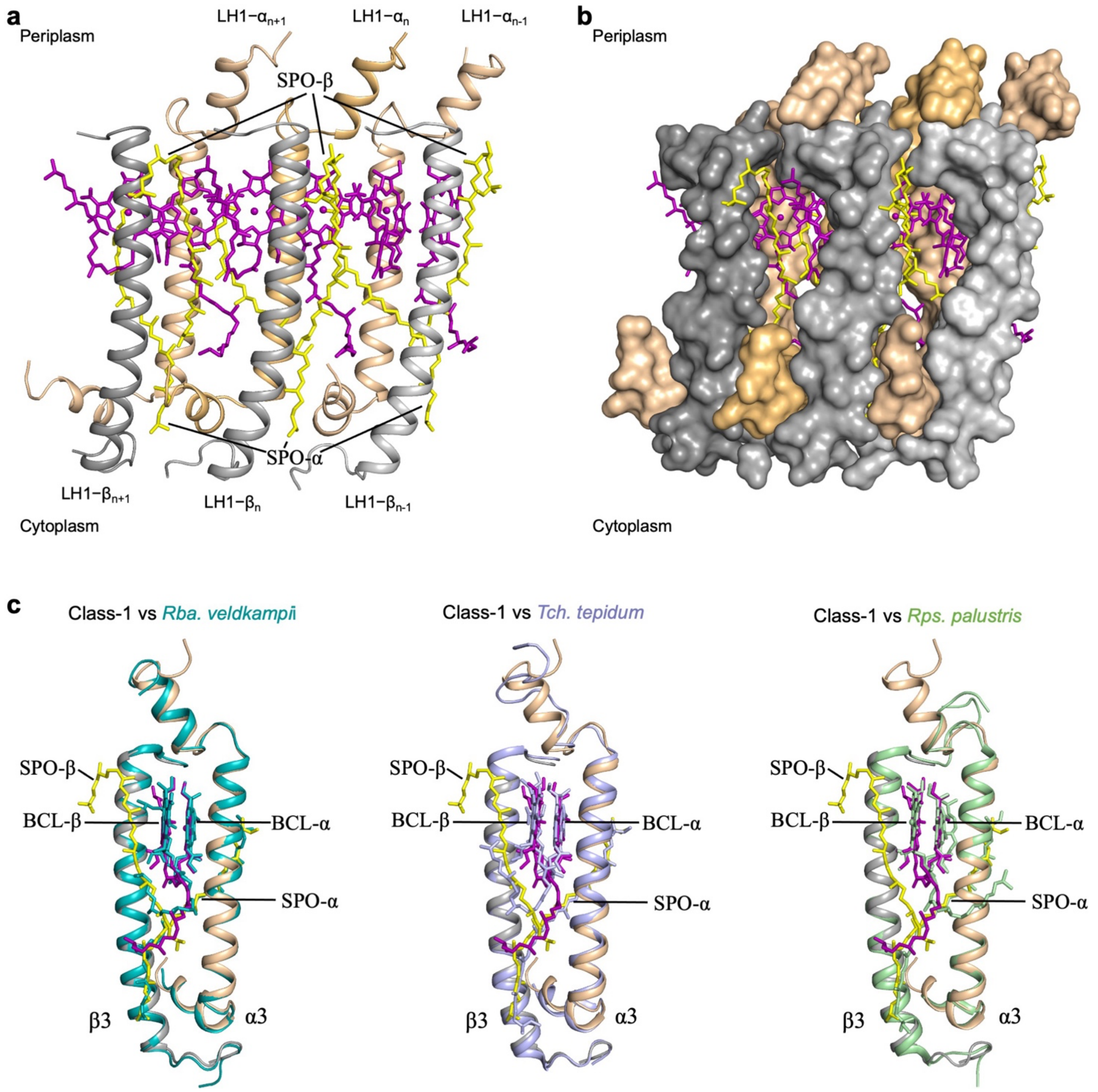

Fig. S16. Analysis of the LH1 structure. (**a**) Architecture of three adjacent LH1 αβ-apoproteins and their bound BChls and SPOs. Pigments are involved in the interaction with neighboring LH1s. (**b**) Dense arrangement of pigment molecules within the LH1 barrier, which potentially blocks the shuttle of quinones/quinols across the LH1 ring. (**c**) Structural comparison of LH1 αβ-heterodimers from the *Rba. sphaeroides* Class-1 dimer (represented by LH1-3) with LH1 from *Rba. veldkampii* (PDB ID: 7DDQ [http://doi.org/10.2210/pdb7DDQ/pdb]), *Tch. tepidum* (PDB ID: 5Y5S [http://doi.org/10.2210/pdb5Y5S/pdb]), and *Rps. palustris* (PDB ID: 6Z5S [http://doi.org/10.2210/pdb6Z5S/pdb]). The presence of a second SPO (SPO-β) is unique in the LH1 of *Rba. sphaeroides*.

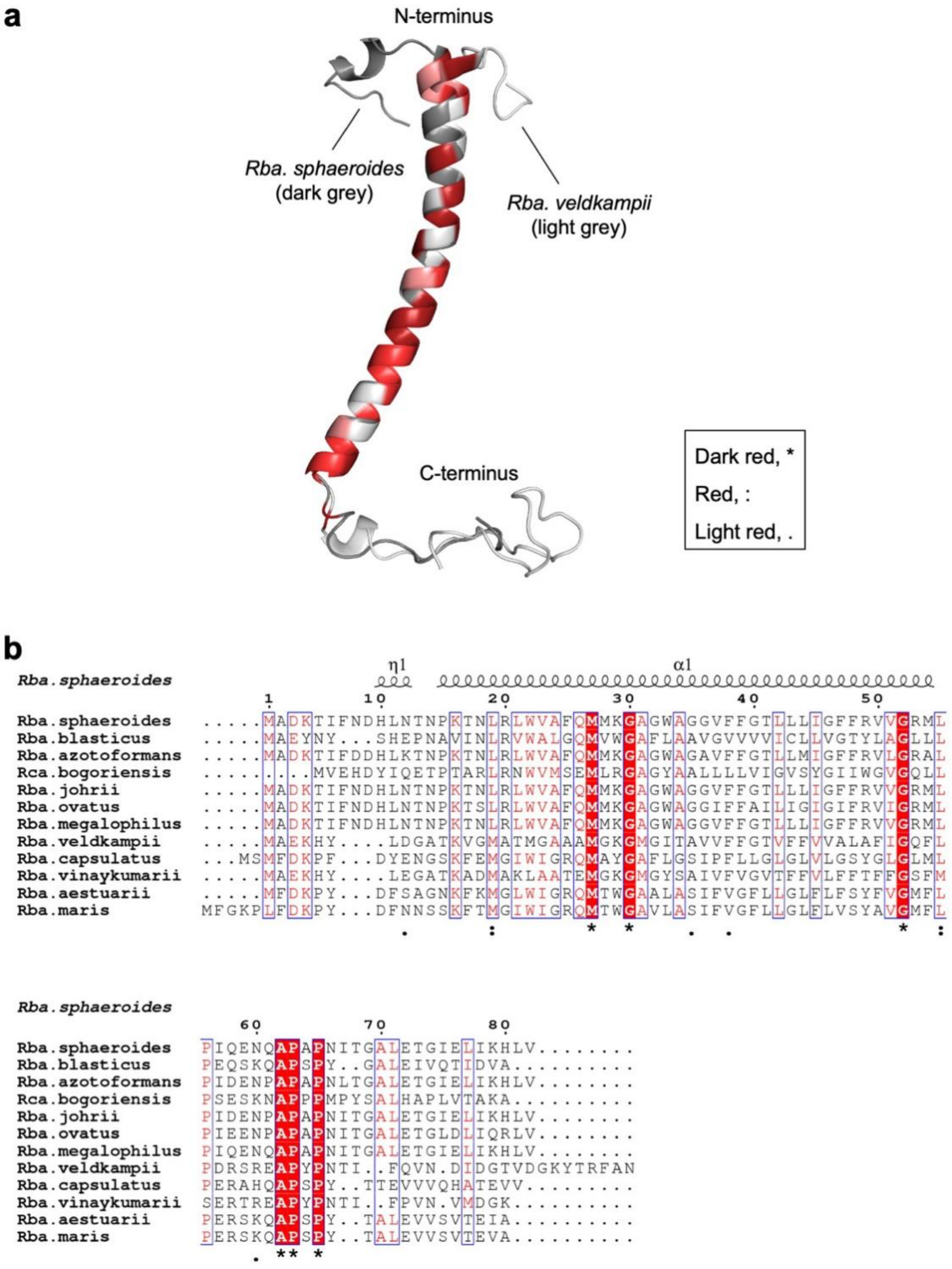

Fig. S17. Comparison of the PufX polypeptides from *Rba. sphaeroides* and other species. (**a**) Comparison of the cryo-EM structure of PufX in the RC−LH1 complexes from *Rba. sphaeroides* and *Rba. veldkampii* (PDB ID: 7DDQ [http://doi.org/10.2210/pdb7DDQ/pdb]). Fully conserved residues are colored in dark red; residues with strongly and weakly similar properties are colored in red and light red, respectively; non-conserved residues are colored in white. (**b**) Sequence alignment of the PufX subunits from *Rba. sphaeroides* and other *Rhodobacter* species. Conserved residues are highlighted.

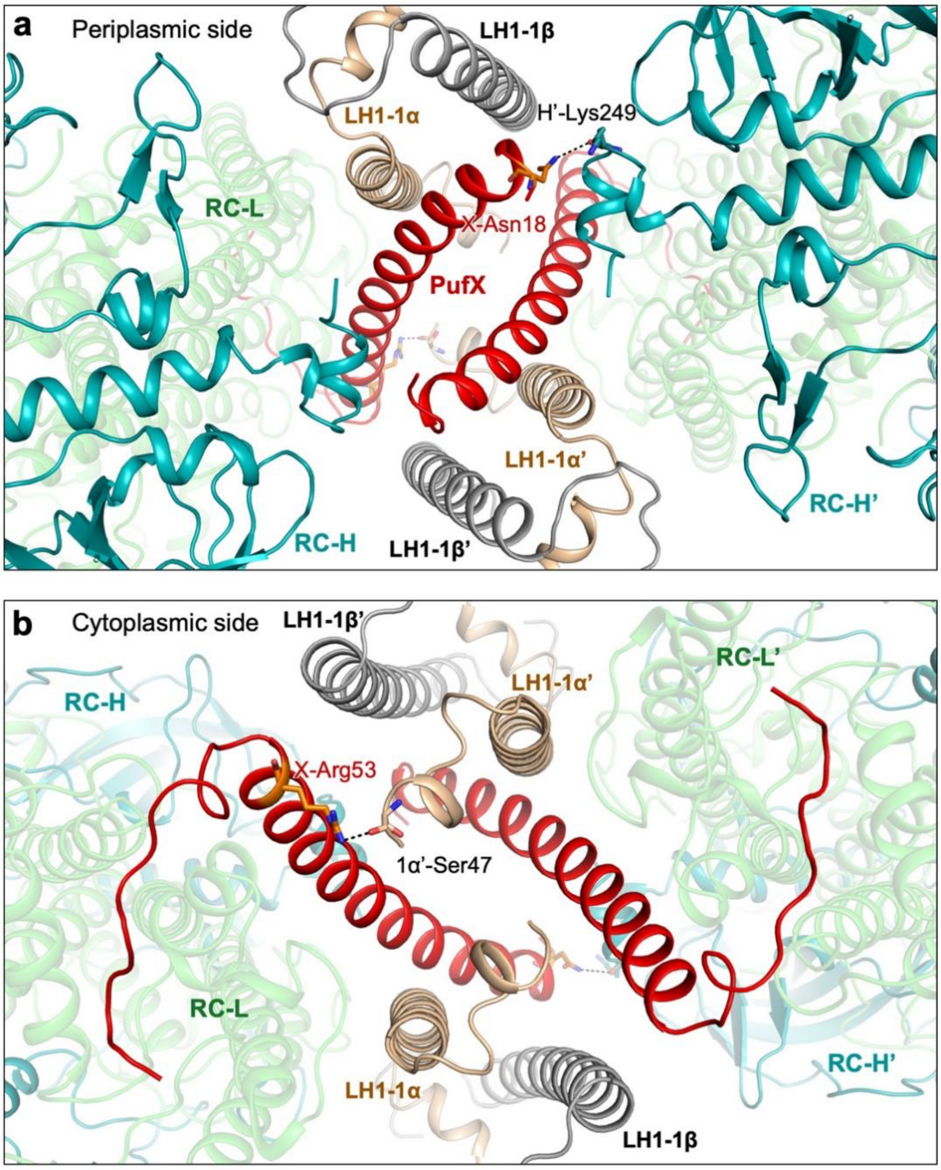

Fig. S18. The interactions of PufX in the Class-2 dimer. (**a**) At the periplasmic side, Asn18 of PufX is hydrogen bonded with the Lys249 residue of RC-H from the neighboring monomer. (**b**) At the cytoplasmic side, Arg53 of PufX forms a hydrogen bond with Ser47 of LH1-1α from the neighboring monomer.

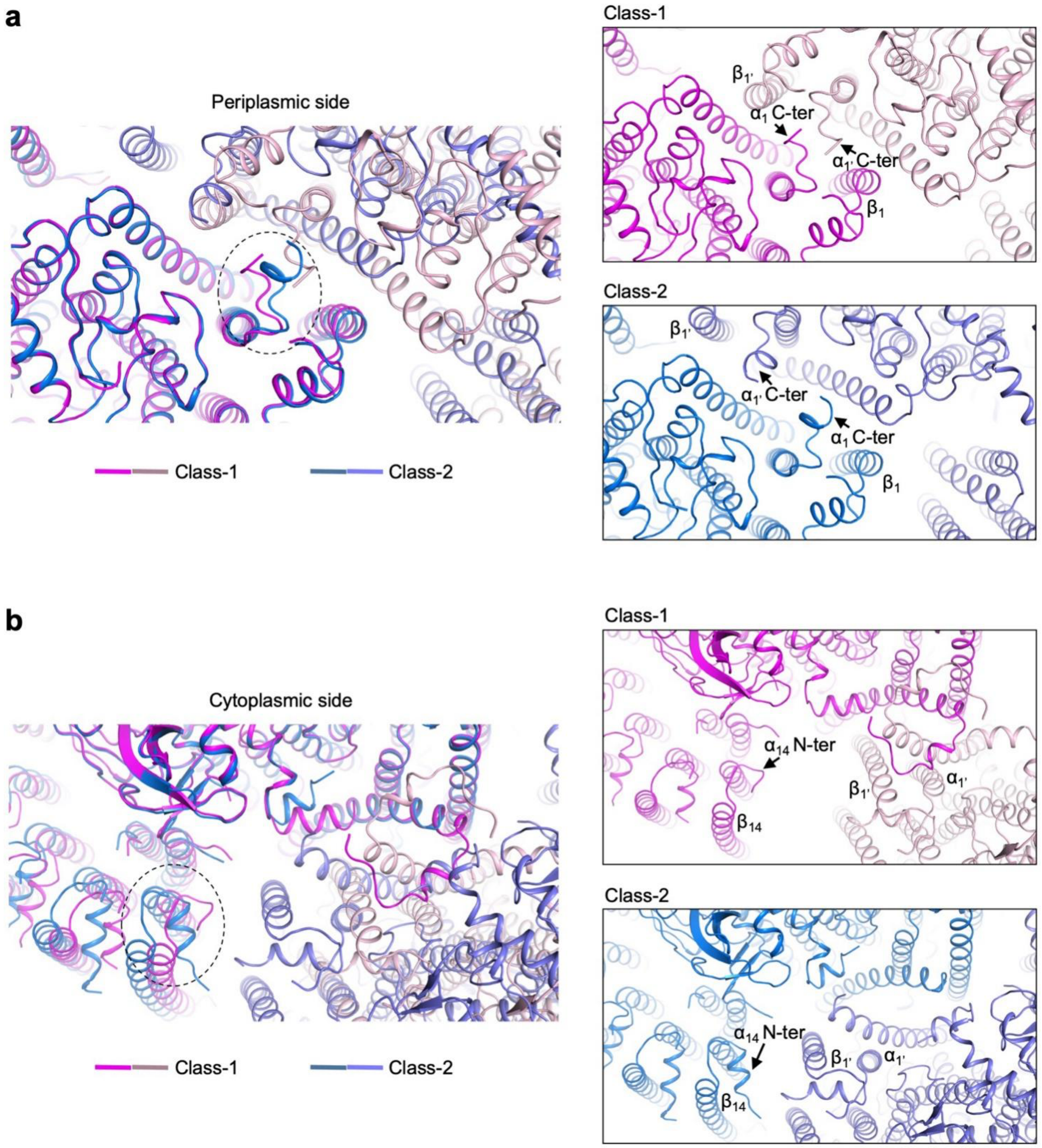

Fig. S19. Structural comparisons of the two classes of RC-LH1 dimers. (**a**) periplasmic view. (**b**) cytoplasmic view. The monomers within the two classes of dimers exhibit structural differences, mainly at the dimerization interface, in particular the last two LH1s and the C-terminal tails of LH1-1α (α_1) as indicated by arrows. At the periplasmic side, the C-terminal tail of LH1-1α (α_1) shows slightly different conformations between Class-1 and Class-2. It is hydrogen bonded with the C-terminal region of LH1-1β (β_1) in Class-1, whereas the two LH1 units are separated without any contacts in Class-2. At the cytoplasmic side, the C-terminal helix of RC-H orientates differently in Class-1 and Class-2. It interacts with the N-terminal region of PufX' in Class-1, whereas the interaction is absent in Class-2. In contrast, the N-terminus of LH1-14α (α_{14}) is better identified in Class-2. It forms contacts with LH1-1β' ($\beta_{1'}$) in Class-2, whereas they are separated in Class-1. Moreover, the last pairs of LH1 slightly shift outward in Class-2 compared with those in Class-1, forming interactions with the neighboring monomer. The closer association of LH1-13/14 region with the neighboring monomer stabilizes the terminal LH1 units and PufY, thus resulting in clearer densities in Class-2.

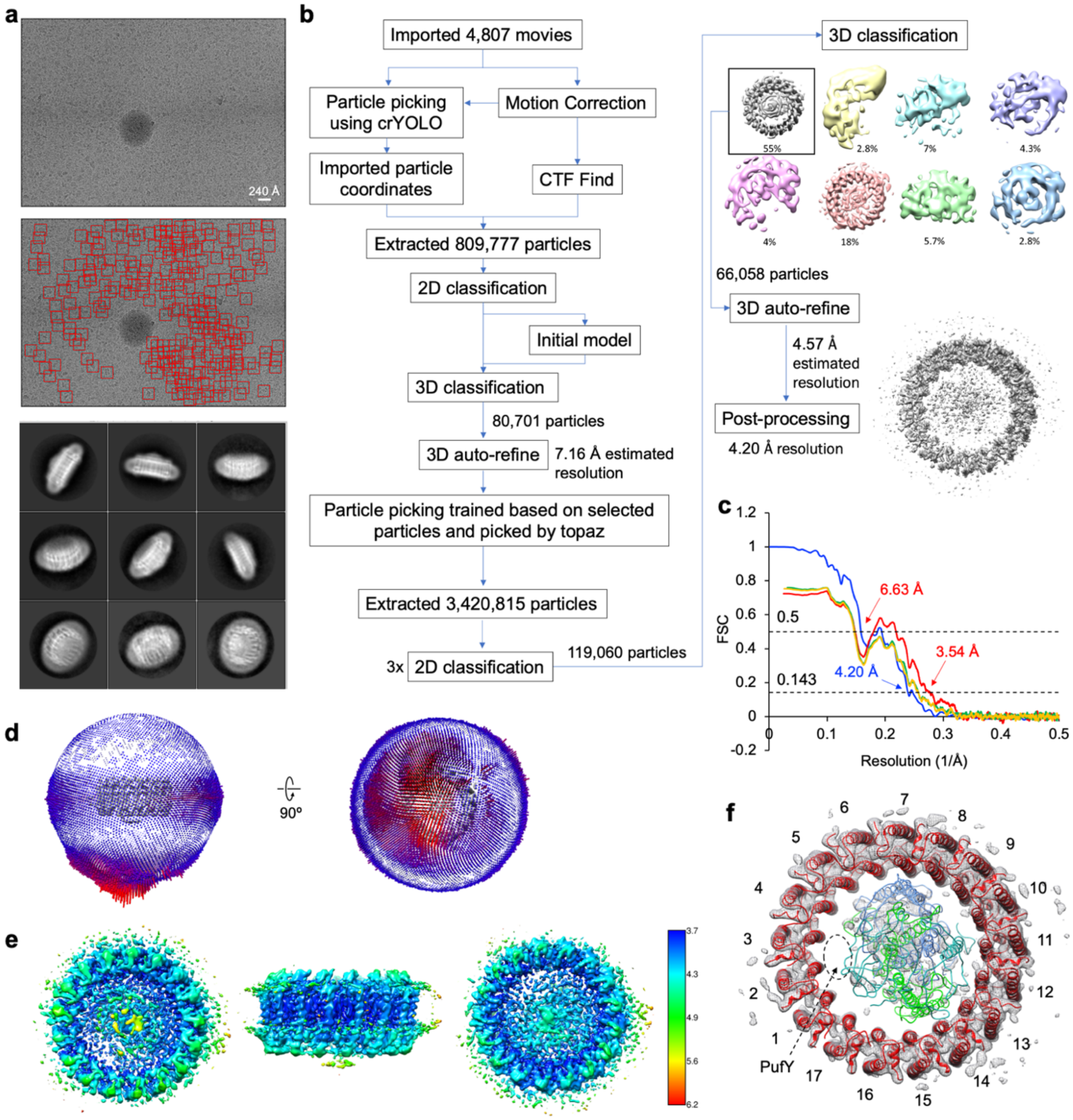

Fig. S20. Cryo-EM data process of the Δ*pufX* RC–LH1 monomer. (**a**) Motion-corrected example of a cryo-EM captured movie (4,807 movies in total) is shown on top and all particles picked from that micrograph are marked with red squares below. Imaged area is 610.6 nm × 433.7 nm. The red squares measure 240 × 240 Å. Representative reference-free 2D class averages are shown at the bottom. (**b**) Overview of cryo-EM data processing workflow for the Δ*pufX* RC–LH1 dataset. Every major step is shown with the associated number of particles, percentage of particles per class, or estimated resolution. Selected 3D class that went into further processing is marked with a rectangle. (**c**) FSC between two independently refined half-maps generated by RELION 3.1 (blue) and model-to-map FSC generated by Phenix (red). Green line: FSC of the model refined against the 1st half map versus the 1st half map; Yellow line: FSC of the model refined against the 1st half map versus the 2nd half map. Global resolutions of 4.20 Å and 3.54 Å were calculated using the FSC cut-off of 0.143 and 6.63 Å using the FSC cut-off of 0.5. Source data are provided as a Source Data file. (**d**) Angular distribution of the particles used to generate the final electron density map. The direction and length of each cylinder represent the view and the number of particles respectively. (**e**) Local resolution of the cryo-EM map as seen from the periplasmic view (left), side view (middle), and the cytoplasmic view (right), estimated by RELION 3.1. (**f**) Cryo-EM map densities of the Δ*pufX* RC–LH1 monomer and the fitting of 17 LH1 subunits and RC subunits. The internal structures of the Δ*pufX* RC–LH1 monomer exhibit poor densities probably due to unstable association with LH1 in the absence of PufX. Hence, PufY was not built in the structure, and its possible location was indicated by a dashed circle and arrow.

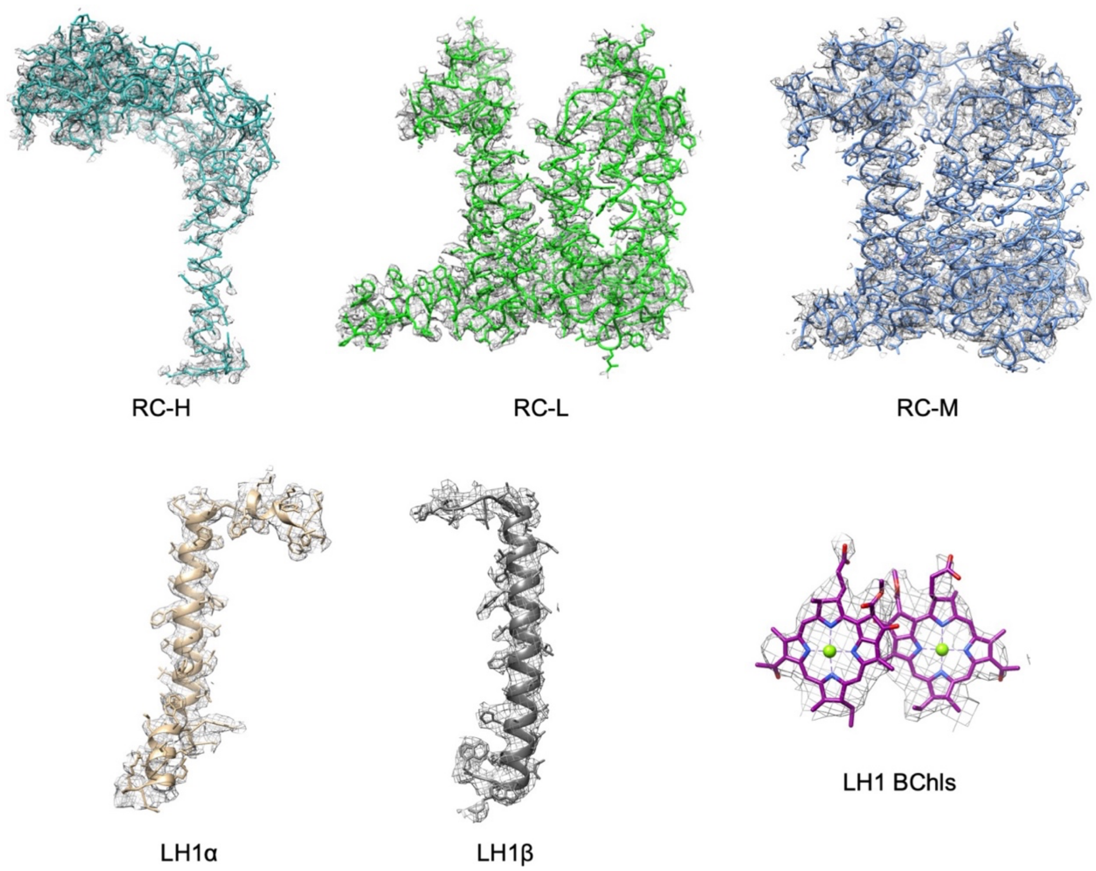

Fig. S21. Cryo-EM map densities and structural models of protein peptides and cofactors in the Δ*pufX* RC–LH1 monomer.

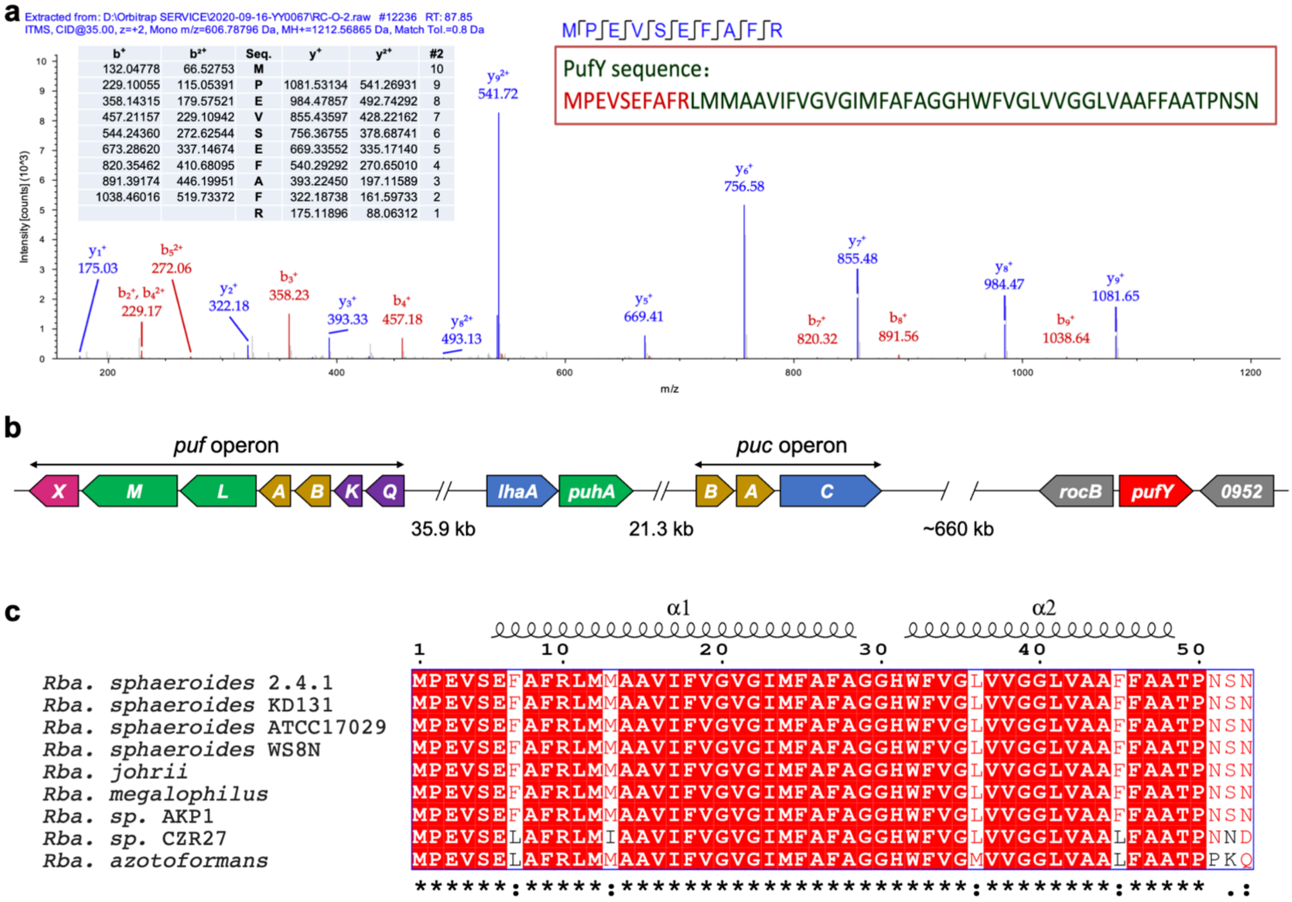

Fig. S22. Identification and analysis of the PufY protein (RSP_7571). (**a**), Liquid chromatography-mass spectrometry (LC-MS/MS) identification of the unique peptide of PufY. Inset: b-ions refer to the N-terminal fragments of the peptide, and y-ions represent the C-terminal fragments. The full PufY sequence is shown in the red box, in which the amino acid residues identified in LC-MS/MS is highlighted in red. (**b**, **c**) Bioinformatic analysis of the *pufY* gene in the genome of *Rba. sphaeroides* (b) and PufY protein sequence alignment (c). PufY was previously annotated as a hypothetical protein in the genome of *Rba. sphaeroides*. Fully conserved residues are highlighted and indicated as "*". ":" indicates the residues with strongly similar properties. "." indicates the residues with weakly similar properties.

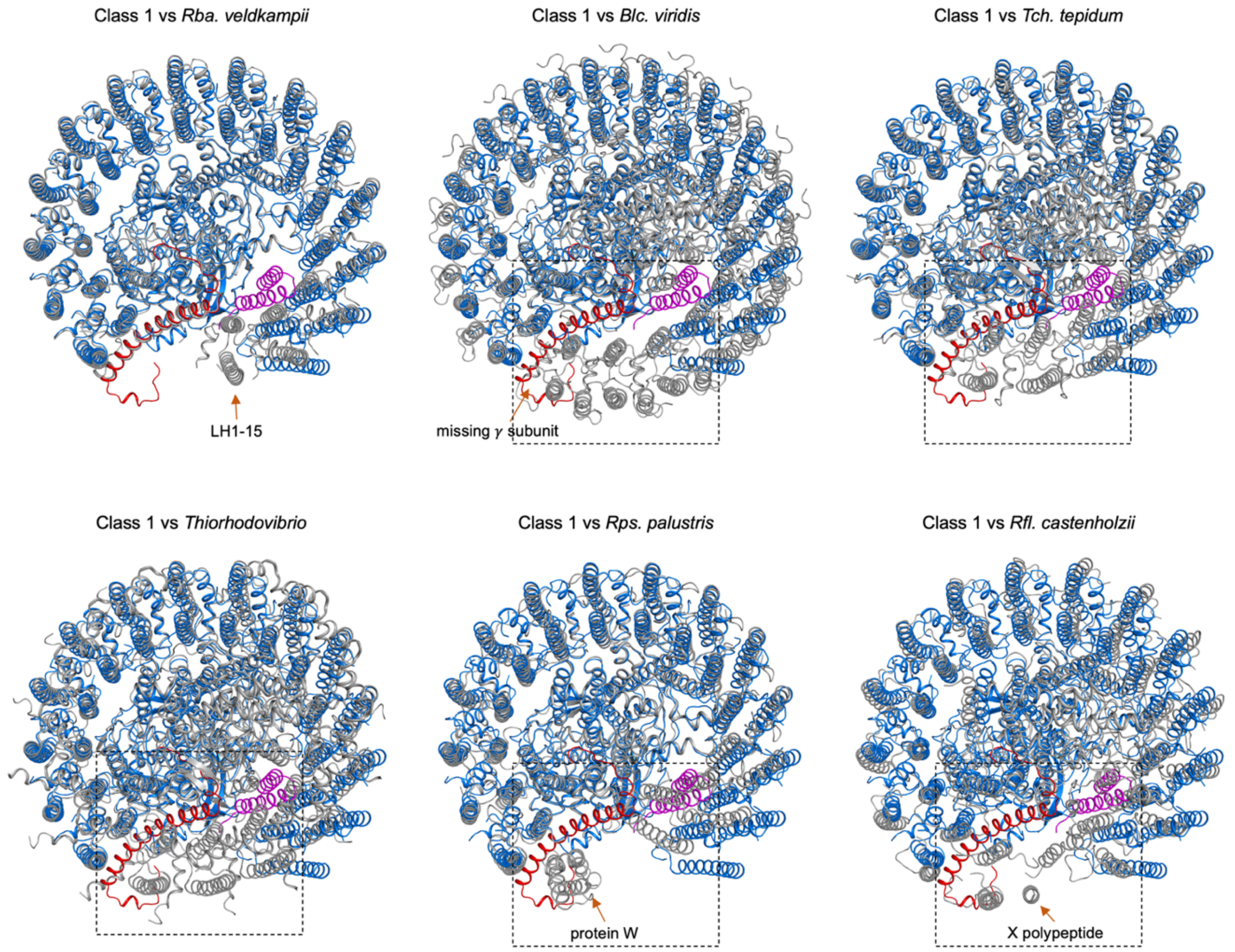

Fig. S23. Structural diversity of the photosynthetic RC−LH1 core complexes from different species. The *Rba. sphaeroides* RC−LH1 monomer in the Class-1 dimer is shown in blue and other RC−LH1 structures are shown in grey. In addition to the presence of PufY (purple), the main structural differences were observed at the large opening regions formed by PufX (red). Compared with the *Rba. sphaeroides* RC−LH1 monomer, the RC−LH1−PufX monomer (PDB ID: 7DDQ [http://doi.org/10.2210/pdb7DDQ/pdb]) of *Rba. veldkampii* has an extra LH1 pair (arrow, 15 LH1 subunits in total); the *Blc. viridis* RC−LH1 (PDB ID: 6ET5 [http://doi.org/10.2210/pdb6ET5/pdb]) has a small gap in the LH1 ring due to the absence of a γ subunit (arrow); the RC−LH1 structures from *Tch. tepidum* (PDB ID: 5Y5S [http://doi.org/10.2210/pdb5Y5S/pdb]) and *Thiorhodovibrio* strain 970 (PDB ID: 7C9R [http://doi.org/10.2210/pdb7C9R/pdb]) have a closed LH1 ring; the *Rps. palustris* RC−LH1 (PDB ID: 1PYH [http://doi.org/10.2210/pdb1PYH/pdb]) has a gap in the LH1 ring created by a protein W (arrow); the *Roseiflexus* (*Rfl.*) *castenholzii* RC−LH1 (PDB ID: 5YQ7 [http://doi.org/10.2210/pdb5YQ7/pdb]) has a gap in the LH1 ring created by an X polypeptide (arrow).

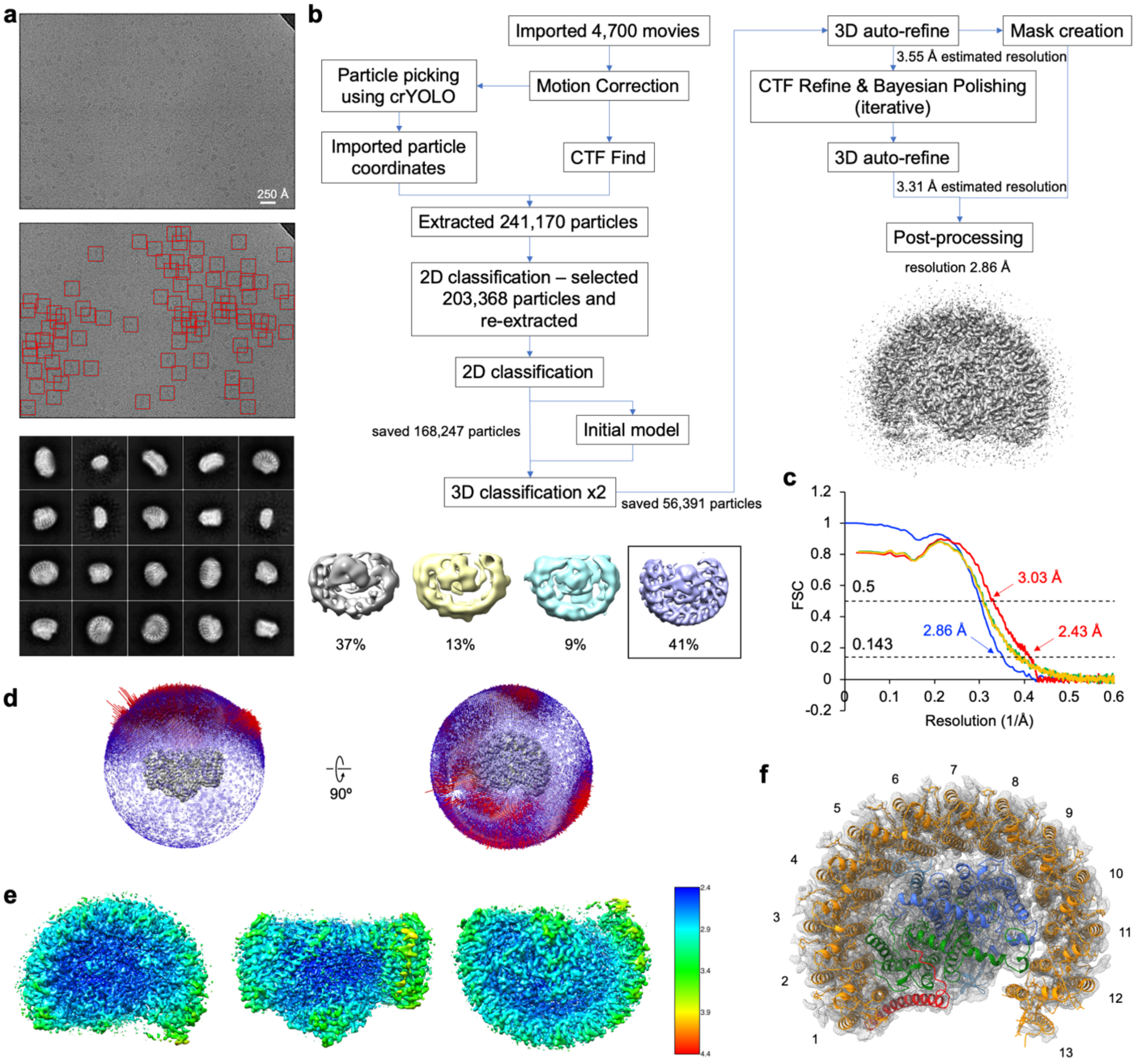

Fig. S24. Cryo-EM data process of the Δ*pufY* RC–LH1 monomer. (**a**) Motion-corrected example of a cryo-EM captured movie (4,700 movies in total) is shown on top and all particles picked from that micrograph are marked with red squares below. Imaged area is 477.2 nm × 339.0 nm. The red squares measure 250 × 250 Å. Representative reference-free 2D class averages are shown at the bottom. (**b**) Overview of cryo-EM data processing workflow for the Δ*pufY* RC–LH1 monomer dataset. Every major step is shown with the associated number of particles, percentage of particles per class, or estimated resolution. Selected 3D class that went into further processing is marked with a rectangle. (**c**) FSC curves including FSC between two independently refined half-maps generated by RELION 3.1 (blue) and model-to-map FSC generated by Phenix (red). Green line: FSC of the model refined against the 1st half map versus the 1st half map; Yellow line: FSC of the model refined against the 1st half map versus the 2nd half map. Global resolutions of 2.86 Å and 2.43 Å were calculated using the FSC cut-off of 0.143. Source data are provided as a Source Data file. (**d**) Angular distribution of the particles used to generate the final electron density map. The direction and length of each cylinder represent the view and the number of particles respectively. (**e**) Local resolution of the cryo-EM map as seen from the periplasmic view (left), side view (middle), and the cytoplasmic view (right), estimated by RELION 3.1. (**f**) Cryo-EM map densities and structural model of protein peptides in the Δ*pufY* RC–LH1 monomer, indicating the presence of PufX and 13 pairs of LH1 αβ-subunits.

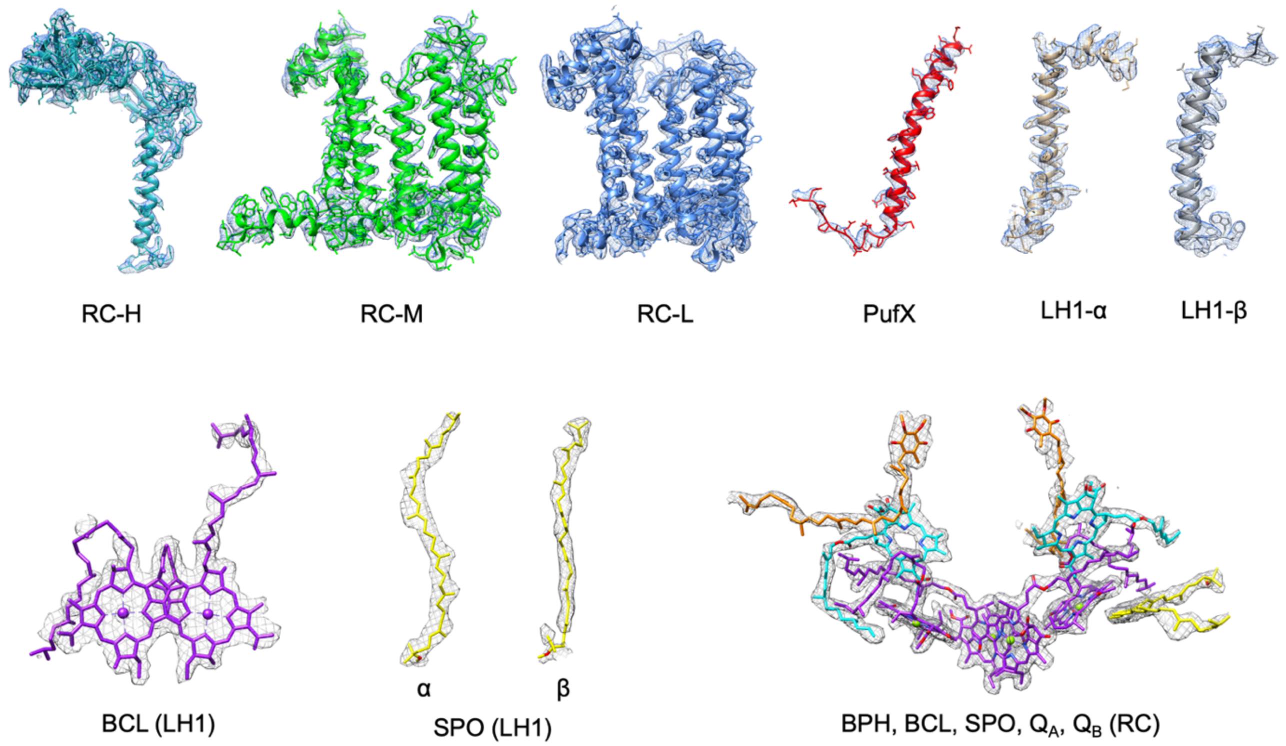

Fig. S25. Cryo-EM map densities and structural models of protein peptides and cofactors in the Δ*pufY* RC–LH1 monomer.

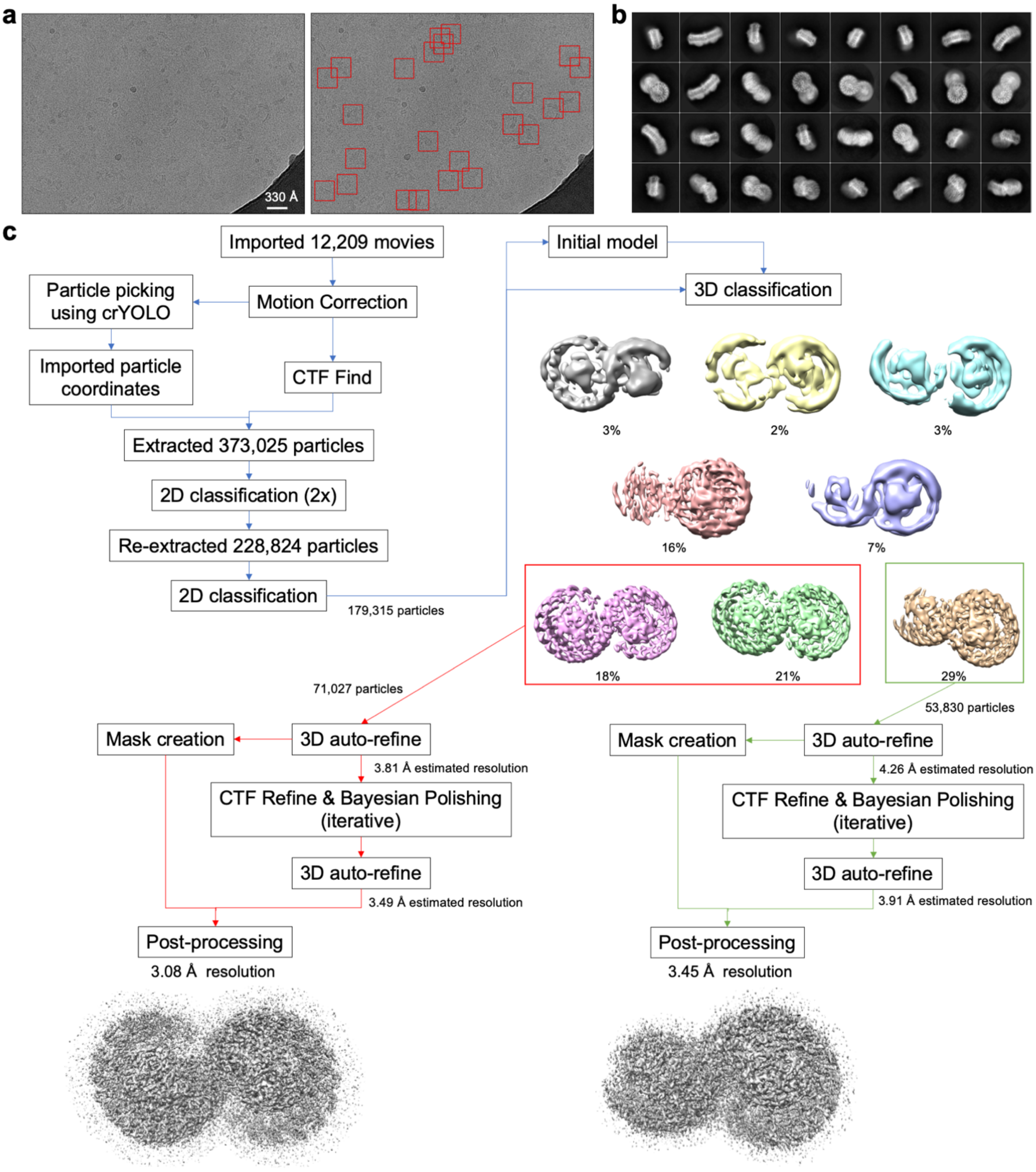

Fig. S26. Cryo-EM analysis of Δ*pufY* RC–LH1 dimers. (**a**) Motion-corrected example of a cryo-EM captured movie (12,209 movies in total) is shown on top and all particles picked from that micrograph are marked with red squares on the right. Imaged area is 477.2 nm × 339.0 nm. The red squares measure 330 × 330 Å. (**b**) Representative reference-free 2D class averages. (**c**) Overview of cryo-EM data processing workflow for the Δ*pufY* RC–LH1 dimer dataset. Every major step is shown with the associated number of particles, percentage of particles per class, or estimated resolution. Selected 3D classes that went into further processing are marked with rectangles (red – complete class, green – incomplete class). Two distinct classes were identified and separately refined, polished and sharpened.

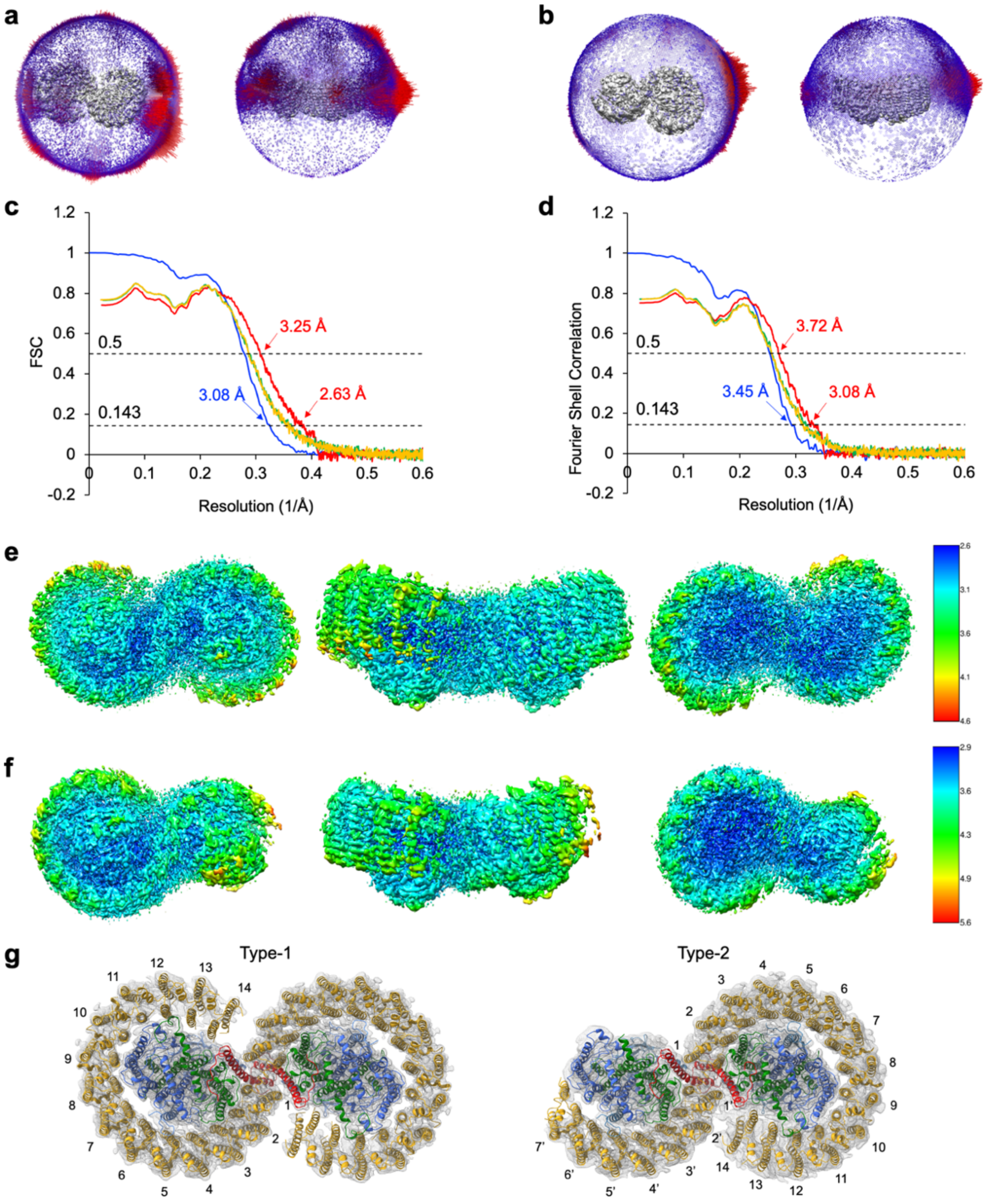

Fig. S27. Cryo-EM data processing of Δ*pufY* RC–LH1 dimers. (**a**-**b**) Angular distribution of the particles used to generate the final electron density maps. The direction and length of each cylinder represent the view and the number of particles respectively. (**a**) Complete (Type-1) dimer. (**b**) Incomplete (Type-2) dimer. (**c**) FSC curves of a complete (Type-1) dimer including FSC between two independently refined half-maps generated by RELION 3.1 (blue) and model-to-map FSC generated by Phenix (red). Global resolutions of 3.08 Å and 2.63 Å were calculated using the FSC cut-off of 0.143. Source data are provided as a Source Data file. (**d**) FSC curves of an incomplete (Type-2) dimer including FSC between two independently refined half-maps generated by RELION 3.1 (blue) and model-to-map FSC generated by Phenix (red). Green line: FSC of the model refined against the 1st half map versus the 1st half map; Yellow line: FSC of the model refined against the 1st half map versus the 2nd half map. Global resolutions of 3.45 Å and 3.08 Å were calculated using the FSC cut-off of 0.143. Source data are provided as a Source Data file. (**e**-**f**) Local resolution of the cryo-EM maps as seen from the cytoplasmic view (left), side view (middle), and the periplasmic view (right), estimated by RELION 3.1. (**e**) Complete (Type-1) dimer. (**f**) Incomplete (Type-2) dimer. (**g**) Cryo-EM map densities and structural model of protein peptides in the Δ*pufY* RC–LH1 dimers. Typ-1 Δ*pufY* RC–LH1 dimer (left) shows a 2-fold symmetry with 13-14 LH1 subunits in each monomer. Type-2 dimer adopts an asymmetric structure, with one monomer consisting of 13-14 LH1 subunits and the other having only 7-9 pairs of LH1 αβ-polypeptides. The last several pairs of LH1 in Type-2 dimer show weaker densities than other LH1s.

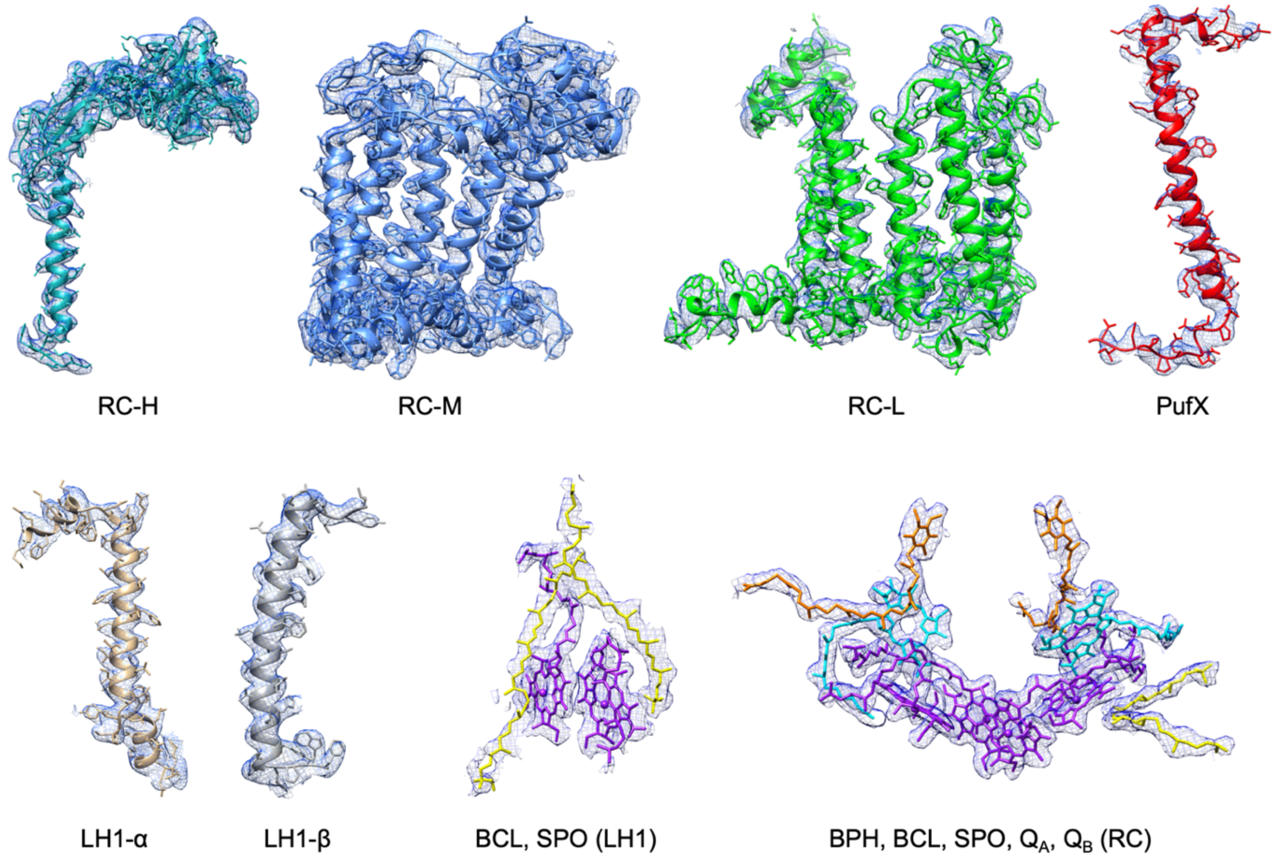

Fig. S28. Cryo-EM map densities and structural models of protein peptides and cofactors in the Δ*pufY* RC–LH1 Type-1 dimer.

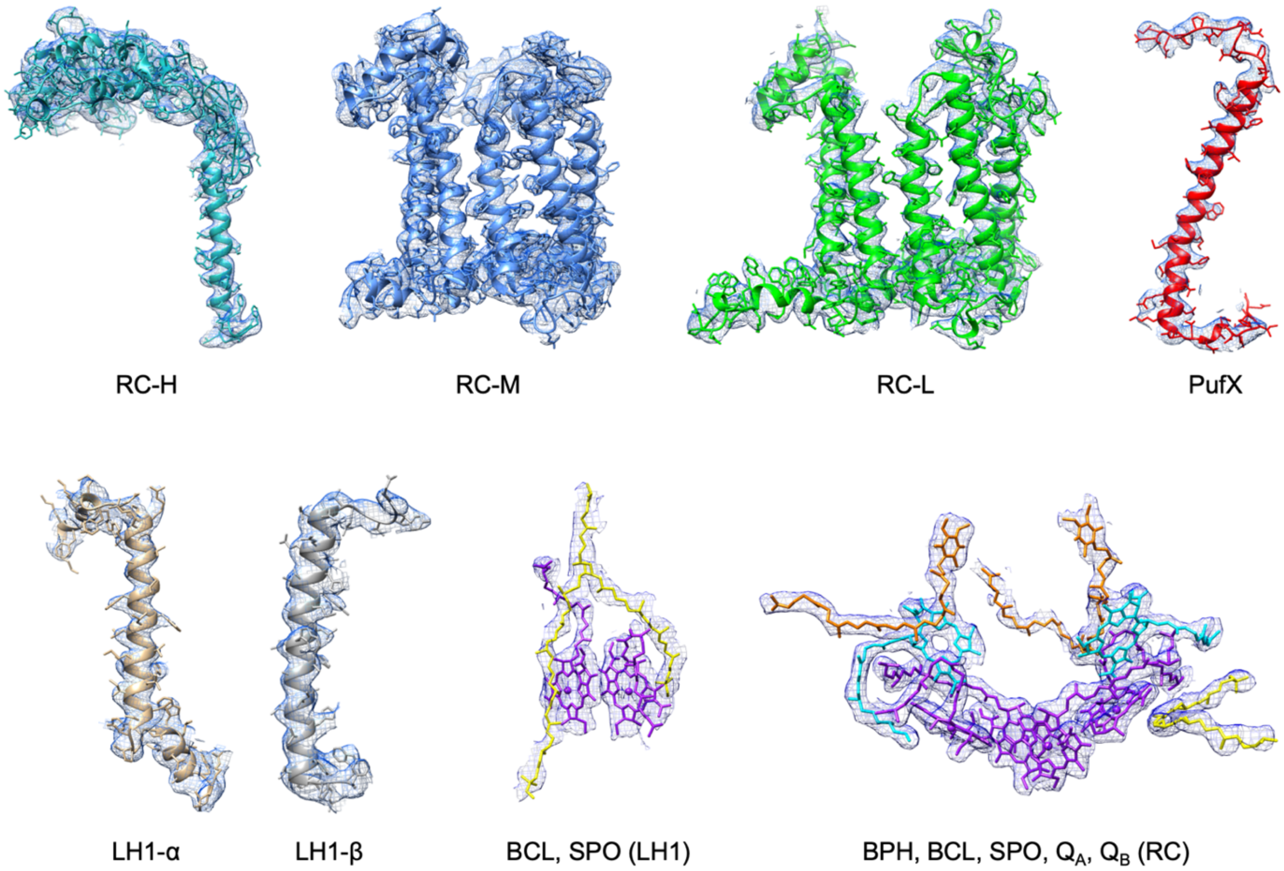

Fig. S29. Cryo-EM map densities and structural models of protein peptides and cofactors in the Δ*pufY* RC–LH1 Type-2 dimer.

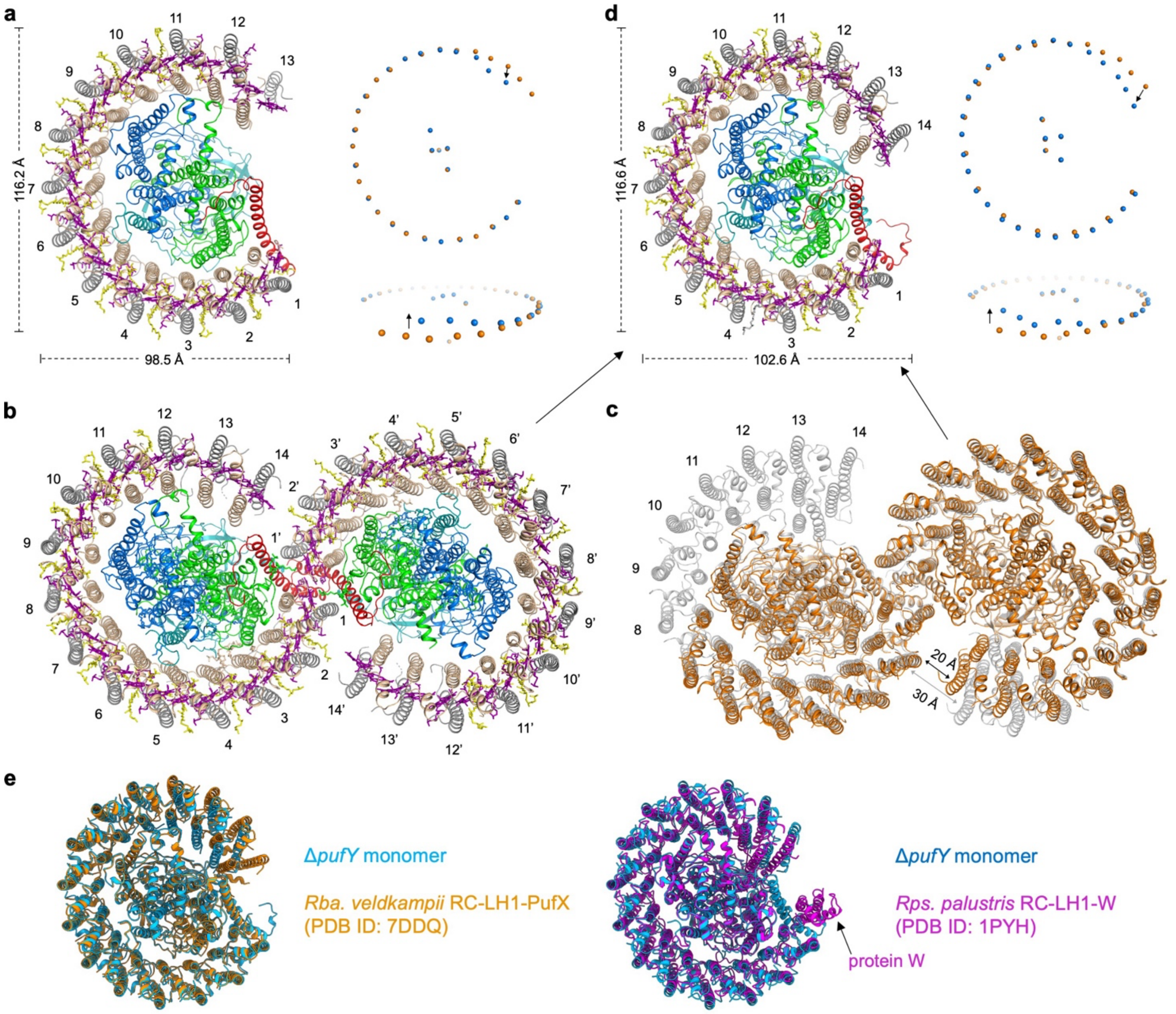

Fig. S30. Δ*pufY* RC–LH1 monomeric and dimeric structures. (**a**) Δ*pufY* RC–LH1 monomer that consists of RC associated with PufX and 13 LH1 subunits. The complex contains an elliptical LH1 ring (116 Å × 98.5 Å) with an enlarged opening in the LH1 array. Right, comparison of the Mg locations in the WT (orange) and Δ*pufY* (blue) RC–LH1 monomers. The absence of PufY caused the organizational shifts of LH1 terminal subunits and the lack of the 14th LH1 subunits. (**b**) Δ*pufY* Type-1 RC–LH1 dimer, with a 2-fold symmetry of 14 LH1 subunits and two PufX located at the monomer-monomer interface. (**c**) Δ*pufY* Type-2 RC–LH1 dimer, with an asymmetric structure and two PufX located at the monomer-monomer interface. One monomer has 14 LH1 subunits and the other has an incomplete LH1 array with 7 LH1 subunits modeled in the structure. (**d**) The monomeric RC–LH1 structure from the Δ*pufY* RC–LH1 dimers, which consists of a more closed, elliptical LH1 ring of 14 subunits (117 Å × 103 Å). Right, comparison of the Mg locations in the WT (orange) and Δ*pufY* (blue) RC–LH1 monomers. The absence of PufY caused the organizational shifts of LH1 terminal subunits. (**e**) In the absence of PufY, the organization of LH1 subunits in the Δ*pufY* RC–$LH1_{14}$ monomer is more comparable to that of the *Rba. veldkampii* RC−LH1−PufX monomer (left, PDB ID: 7DDQ [http://doi.org/10.2210/pdb7DDQ/pdb]) than that of the *Rps. palustris* RC−LH1 (right, PDB ID: 1PYH [http://doi.org/10.2210/pdb1PYH/pdb]) containing a protein W (arrow).

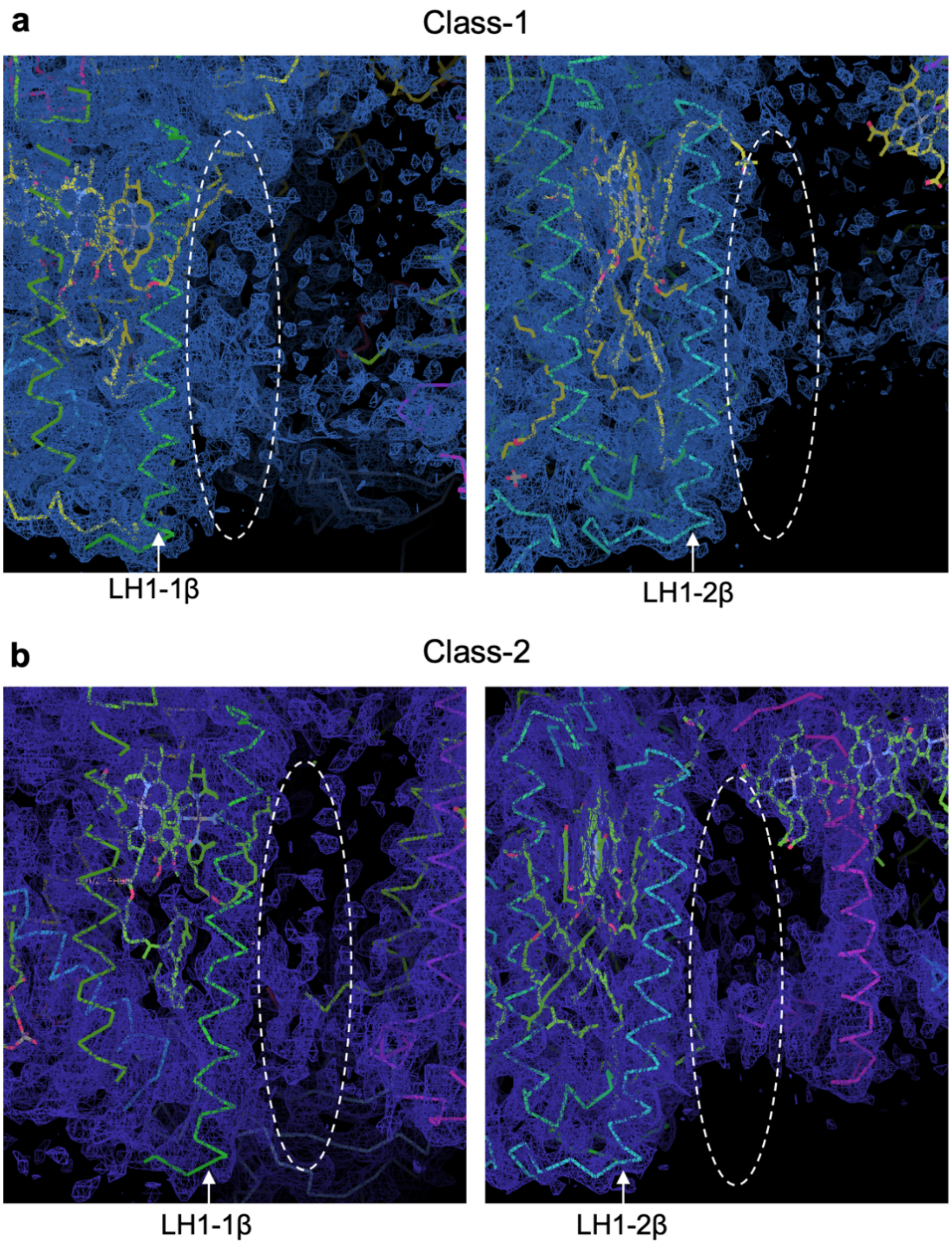

Fig. S31. Cryo-EM density maps showing the densities at the potential locations of "protein-Z" [15] (indicated by dash circles) near LH1-1β (left) and LH1-2β (right). (**a**) Local densities in the WT Class-1 dimer structure. (**b**) Local densities in the WT Class-2 dimer structure.

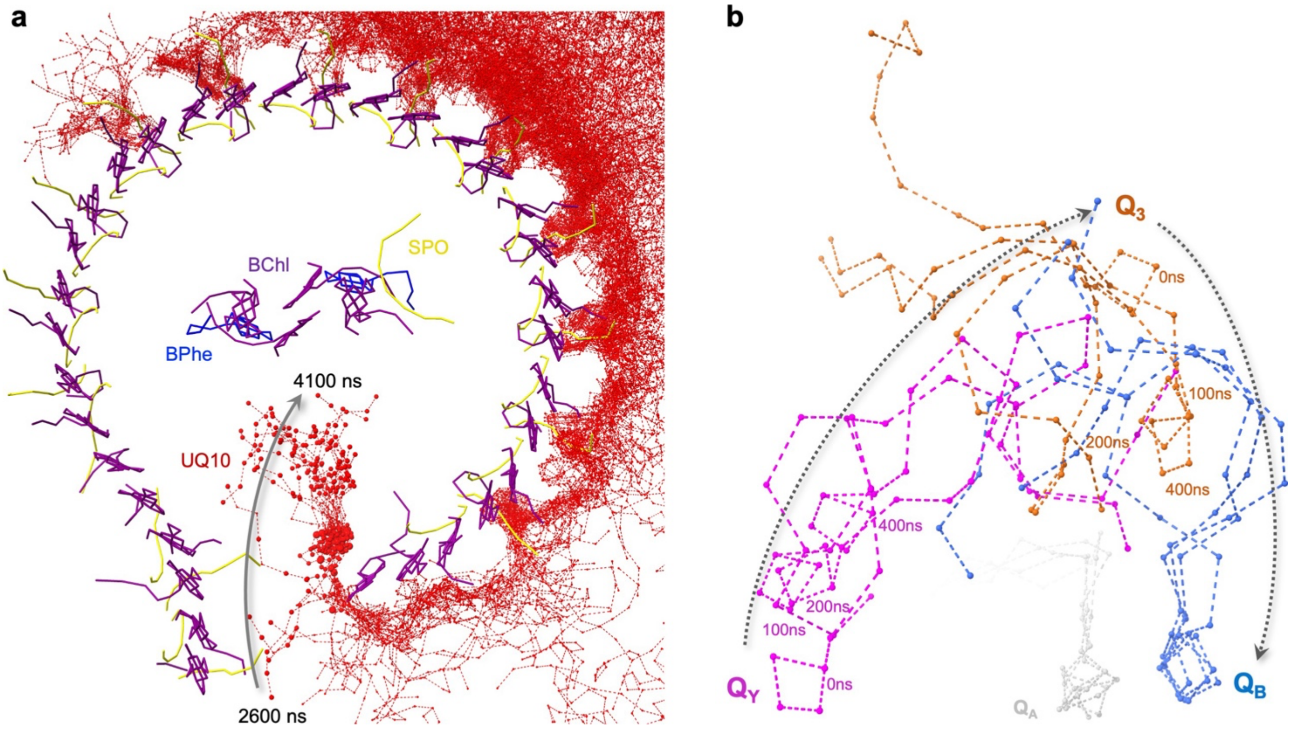

Fig. S32. MD simulations of quinone movement within the dimeric RC−LH1 complex. (**a**) CGMD simulations at the 5 µs timescale using a quinone-free RC−LH1 dimeric structure show quinones can enter the RC−LH1 complex through the large opening of the S-shaped LH1 ring as the predominant quinone transport pathway. See also Movie S2 (Entering of quinone into the complex occurred during 2600 and 4100 ns). (**b**) CGMD simulations using the RC−LH1 dimeric structure bound with Q_A (grey), Q_B (blue), Q_3 (orange), and Q_Y (magenta) show the movement and conformational shifts of Q_Y and Q_3. Dashed arrows imply the possible movement of Q_Y towards Q_3 and Q_3 towards Q_B within 300 ns. Q_A and Q_B are relatively stable, whereas Q_Y exhibits a greater conformational flexibility. See Movies S3 and S4.

```
RC-L            MALLSFERKYRVPGGTLVGGNLFDFWVGPFYVGFFGVATFFFAALGIILIAWSAVLQGTWNPQLISVYPPALEYGLGGAPLAKGGLW
                QIITICATGAFVSWALREVEICRKLGIGYHIPFAFAFAILAYLTLVLFRPVMMGAWGYAFPYGIWTHLDWVSNTGYTYGNFHYNPAH
                MIAISFFFTNALALALHGALVLSAANPEKGKEMRTPDHEDTFFRDLVGYSIGTLGIHRLGLLLSLSAVFFSALCMIITGTIWFDQWV
                DWWQWWVKLPWWANIPGGING

RC-M            MAEYQNIFSQVQVRGPADLGMTEDVNLANRSGVGPFSTLLGWFGNAQLGPIYLGSLGVLSLFSGLMWFFTIGIWFWYQAGWNPAVFL
                RDLFFFSLEPPAPEYGLSFAAPLKEGGLWLIASFFMFVAVWSWWGRTYLRAQALGMGKHTAWAFLSAIWLWMVLGFIRPILMGSWSE
                AVPYGIFSHLDWTNNFSLVHGNLFYNPFHGLSIAFLYGSALLFAMHGATILAVSRFGGERELEQIADRGTAAERAALFWRWTMGFNA
                TMEGIHRWAIWMAVLVTLTGGIGILLSGTVVDNWYVWGQNHGMAPLN

RC-H            MVGVTAFGNFDLASLAIYSFWIFLAGLIYYLQTENMREGYPLENEDGTPAANQGPFPLPKPKTFILPHGRGTLTVPGPESEDRPIAL
                ARTAVSEGFPHAPTGDPMKDGVGPASWVARRDLPELDGHGHNKIKPMKAAAGFHVSAGKNPIGLPVRGCDLEIAGKVVDIWVDIPEQ
                MARFLEVELKDGSTRLLPMQMVKVQSNRVHVNALSSDLFAGIPTIKSPTEVTLLEEDKICGYVAGGLMYAAPKRKSVVAAMLAEYA

Class-1 dimer   MVGVTAFGNFDLASLAIYSFWIFLAGLIYYLQTENMREGYPLENEDGTPAANQGPFPLPKPKTFILPHGRGTLTVPGPESEDRPIAL
                ARTAVSEGFPHAPTGDPMKDGVGPASWVARRDLPELDGHGHNKIKPMKAAAGFHVSAGKNPIGLPVRGCDLEIAGKVVDIWVDIPEQ
                MARFLEVELKDGSTRLLPMQMVKVQSNRVHVNALSSDLFAGIPTIKSPTEVTLLEEDKICGYVAGLMYAAPKRKSVVAAMLAEYA

Class-2 dimer   MVGVTAFGNFDLASLAIYSFWIFLAGLIYYLQTENMREGYPLENEDGTPAANQGPFPLPKPKTFILPHGRGTLTVPGPESEDRPIAL
                ARTAVSEGFPHAPTGDPMKDGVGPASWVARRDLPELDGHGHNKIKPMKAAAGFHVSAGKNPIGLPVRGCDLEIAGKVVDIWVDIPEQ
                MARFLEVELKDGSTRLLPMQMVKVQSNRVHVNALSSDLFAGIPTIKSPTEVTLLEEDKICGYVAGLMYAAPKRKSVVAAMLAEYA

WT monomer      MVGVTAFGNFDLASLAIYSFWIFLAGLIYYLQTENMREGYPLENEDGTPAANQGPFPLPKPKTFILPHGRGTLTVPGPESEDRPIAL
                ARTAVSEGFPHAPTGDPMKDGVGPASWVARRDLPELDGHGHNKIKPMKAAAGFHVSAGKNPIGLPVRGCDLEIAGKVVDIWVDIPEQ
                MARFLEVELKDGSTRLLPMQMVKVQSNRVHVNALSSDLFAGIPTIKSPTEVTLLEEDKICGYVAGLMYAAPKRKSVVAAMLAEYA

LH1α            MSKFYKIWMIFDPRRVFVAQGVFLFLLAVMIHLILLSTPSYNWLEISAAKYNRVAVAE
Class-1 dimer   MSKFYKIWMIFDPRRVFVAQGVFLFLLAVMIHLILLSTPSYNWLEISAAKYNRVAVAE
Class-2 dimer   MSKFYKIWMIFDPRRVFVAQGVFLFLLAVMIHLILLSTPSYNWLEISAAKYNRVAVAE
WT monomer      MSKFYKIWMIFDPRRVFVAQGVFLFLLAVMIHLILLSTPSYNWLEISAAKYNRVAVAE

LH1β            MADKSDLGYTGLTDEQAQELHSVYMSGLWLFSAVAIVAHLAVYIWRPWF
Class-1 dimer   MADKSDLGYTGLTDEQAQELHSVYMSGLWLFSAVAIVAHLAVYIWRPWF
Class-2 dimer   MADKSDLGYTGLTDEQAQELHSVYMSGLWLFSAVAIVAHLAVYIWRPWF
WT monomer      MADKSDLGYTGLTDEQAQELHSVYMSGLWLFSAVAIVAHLAVYIWRPWF

PufX            MADKTIFNDHLNTNPKTNLRLWVAFQMMKGAGWAGGVFFGTLLLIGFFRVVGRMLPIQENQAPAPNITGALETGIELIKHLV
Class-1 dimer   MADKTIFNDHLNTNPKTNLRLWVAFQMMKGAGWAGGVFFGTLLLIGFFRVVGRMLPIQENQAPAPNITGALETGIELIKHLV
Class-2 dimer   MADKTIFNDHLNTNPKTNLRLWVAFQMMKGAGWAGGVFFGTLLLIGFFRVVGRMLPIQENQAPAPNITGALETGIELIKHLV
WT monomer      MADKTIFNDHLNTNPKTNLRLWVAFQMMKGAGWAGGVFFGTLLLIGFFRVVGRMLPIQENQAPAPNITGALETGIELIKHLV

PufY            MPEVSEFAFRLMMAAVIFVGVGIMFAFAGGHWFVGLVVGGLVAAFFAATPNSN
Class-1 dimer   MPEVSEFAFRLMMAAVIFVGVGIMFAFAGGHWFVGLVVGGLVAAFFAATPNSN
Class-2 dimer   MPEVSEFAFRLMMAAVIFVGVGIMFAFAGGHWFVGLVVGGLVAAFFAATPNSN
WT monomer      MPEVSEFAFRLMMAAVIFVGVGIMFAFAGGHWFVGLVVGGLVAAFFAATPNSN
```

Fig. S33. Amino acid sequences of protein polypeptides in the RC−LH1 complex from *Rba. sphaeroides*, and resolved residues in the dimeric and monomeric structures. Protein residues that were not resolved in the structures of Class-1 and Class-2 dimers and the WT monomer are highlighted in red. Protein Uniprot ID: RC-L, Q3J1A5 [https://www.uniprot.org/uniprot/Q3J1A5]; RC-M, Q3J1A6 [https://www.uniprot.org/uniprot/Q3J1A6]; RC-H, Q3J170 [https://www.uniprot.org/uniprot/Q3J170]; LH1-α, Q3J1A4 [https://www.uniprot.org/uniprot/Q3J1A4]; LH1-β, Q3J1A3 [https://www.uniprot.org/uniprot/Q3J1A3]; PufX, P13402 [https://www.uniprot.org/uniprot/P13402]; PufY, U5NME9 [https://www.uniprot.org/uniprot/U5NME9].

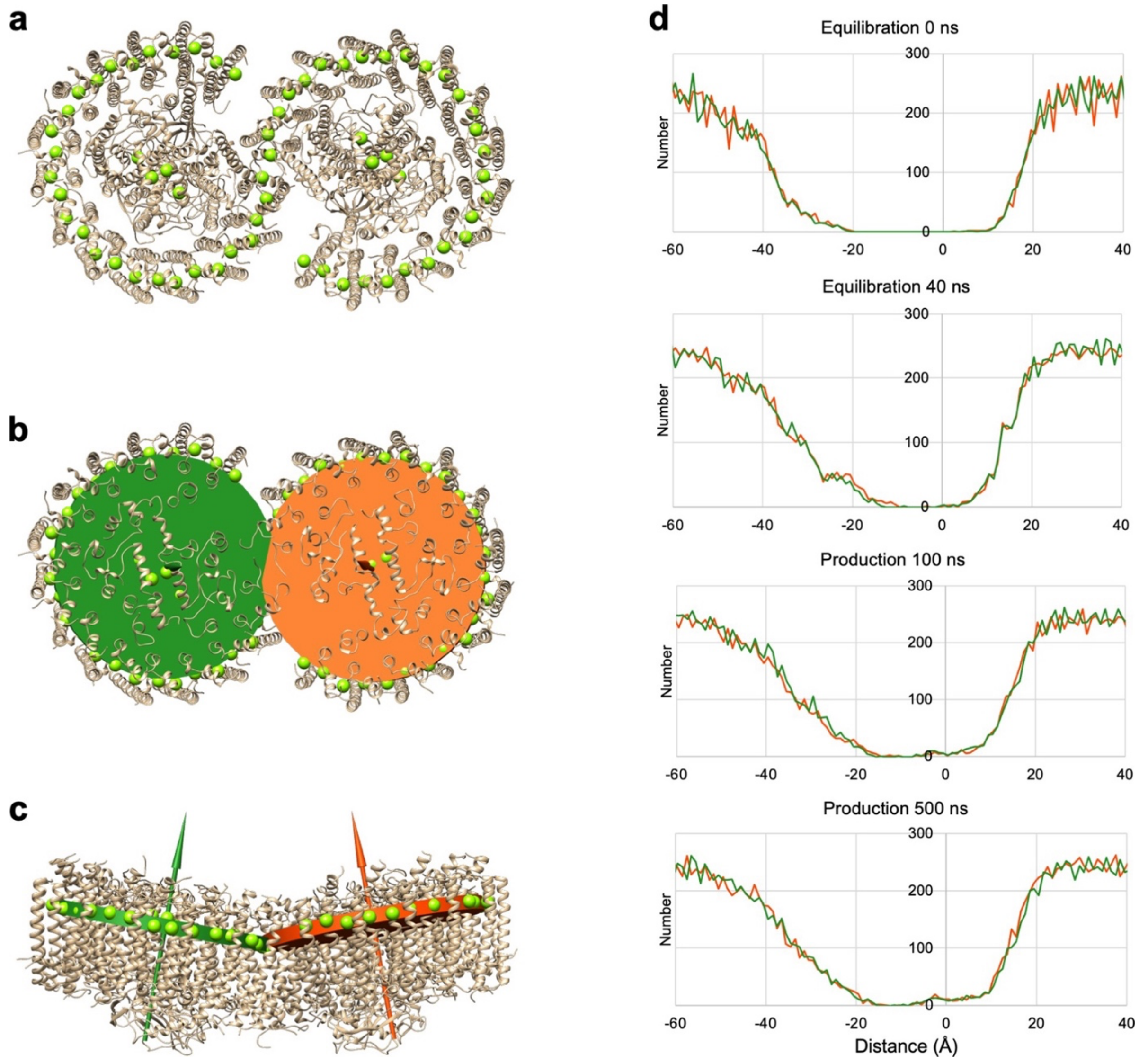

Fig. S34. Distribution of water molecules in the simulations system. (**a**) Top view of the RC-LH1 dimer. (**b**) A circle is defined by the Mg^{2+} ion (light green sphere) bound to the LH1 array in each monomer with the dimer. (**c**) A cylinder is developed by moving the circle along its normal vector (orange and green arrows). (**d**) Distribution of water molecules within the cylinder along the normal vector between -60 Å and 40 Å at 0 ns and 40 ns of the equilibration run and 100 ns and 500 ns of the production run. Green and orange profiles correspond to the distribution of water molecules within the green and orange cylinders along the normal vectors shown in panels **b**-**c**. Zero Å represents the position of the Mg^{2+} ion. The number of water molecules falls to zero near the center of the complex, indicating that no water exists in the inner membrane region (some water molecules appear within the RC, forming hydrogen bonds with polar residues of the RC). Source data are provided as a Source Data file.

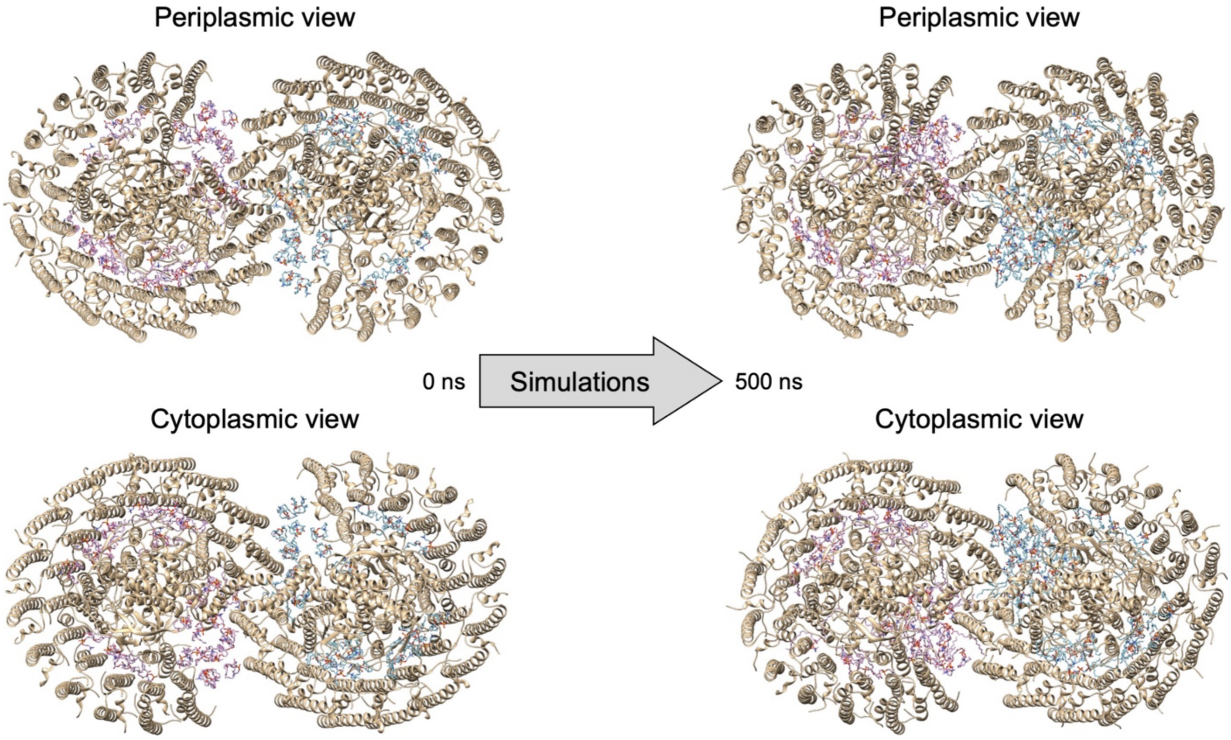

Fig. S35. The lipid arrangement within the RC-LH1 dimer before and after simulations.

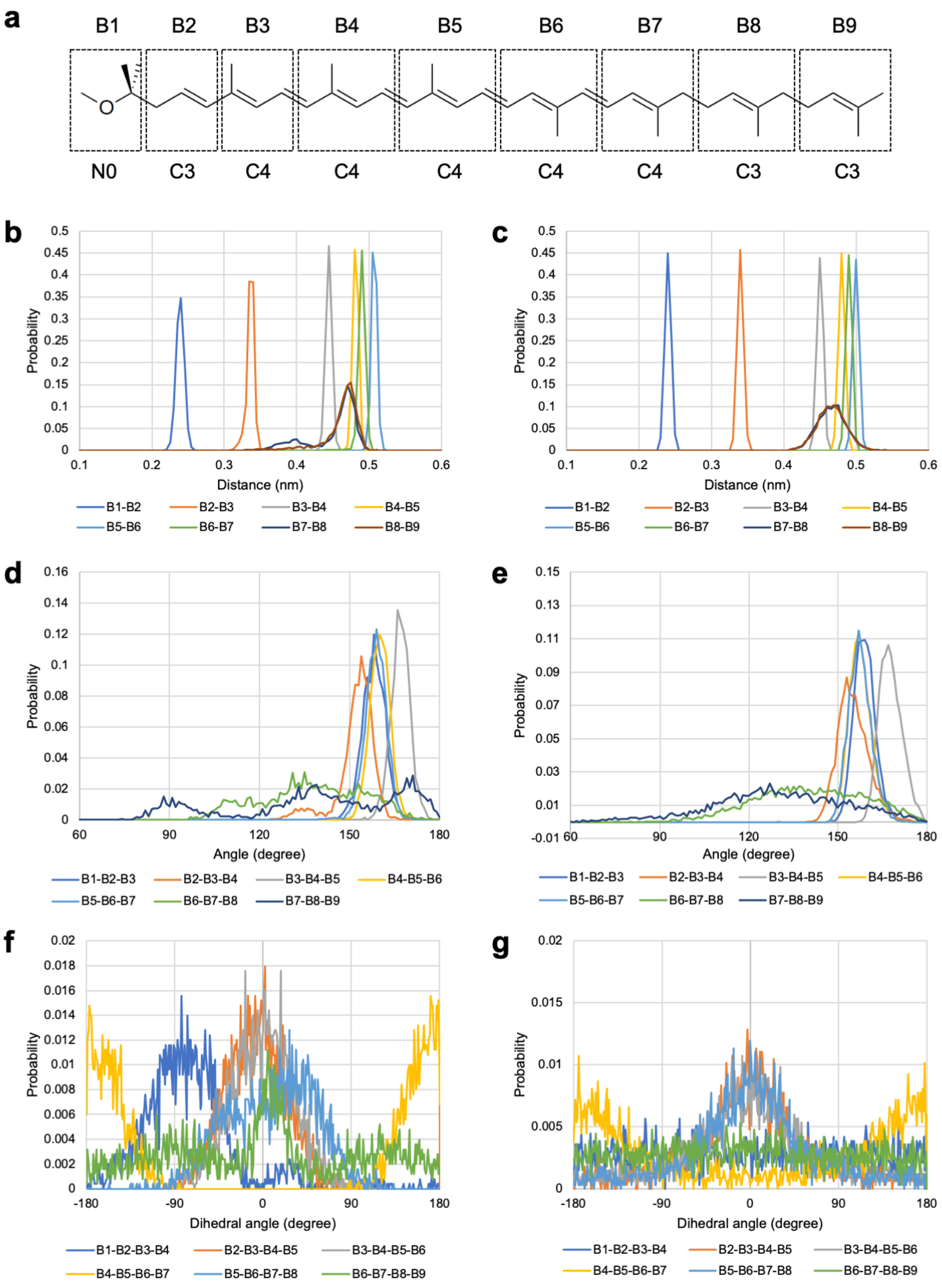

Fig. S36. Setting the coarse-grained models of cofactors. (**a**) Mapping of SPO atoms to coarse-grained (CG) beads. A CG bead was placed at the center of mass of the atoms within a dashed square. The name and the type of each CG bead are shown above and below the structure, respectively. (**b-g**) Comparison of the probability distributions of bond lengths (**b**, **c**), angles (**d**, **e**), and dihedral angles (**f**, **g**) between the AAMD (**b**, **d**, **f**) and CGMD (**c**, **e**, **g**) simulations.

SUPPLEMENTARY TABLES

Table S1. Cryo-EM data collection, refinement and validation statistics (to be continued).

	**WT dimer Class-1** (EMD-32058) (PDB 7VOR)	**WT dimer Class-2** (EMD-32059) (PDB 7VOT)	**WT monomer** (EMD-32047) (PDB 7VNY)
**Data collection and processing**			
Magnification	130,000 x	-	81,000 x
Voltage (kV)	300	-	300
Electron exposure ($e^{-}/Å^2$)	60	-	45.026
Defocus range (μm)	1.8-2.2	-	0.8-1.8
Pixel size (Å)	1.04	-	1.06
Symmetry imposed	C2	C2	C1
Initial particle images (no.)	957,176	-	241,170
Final particle images (no.)	145,392	147,085	68,554
Map resolution (Å)	2.74	2.90	2.79
FSC threshold	0.143	0.143	0.143
Map resolution range (Å)	2.5-5.3	2.7-5.2	2.3-4.5
**Refinement**			
Initial model used (PDB code)	5Y5S	Class-1 dimer	Class-2 dimer
Model resolution (Å)	2.74	2.90	2.76
FSC threshold	0.143	0.143	0.143
Map resolution range (Å)	-	-	-
Map sharpening *B* factor ($Å^2$)	-41.0	-47.0	-86.3
Model composition			
Non-hydrogen atoms	44,980	44,984	22,506
Protein residues	4,614	4,594	2,284
Ligands	148	152	76
B factors ($Å^2$)			
Protein	38.6	52.4	68.98
Ligand	39.3	44.4	63.88
R.m.s. deviations			
Bond lengths (Å)	0.006	0.005	0.012
Bond angles (°)	0.917	0.726	1.159
**Validation**			
MolProbity score	1.45	1.48	1.64
Clashscore	8.3	9.0	10.46
Poor rotamers (%)	0.37	0.37	0.05
Ramachandran plot			
Favored (%)	98.2	98.5	97.47
Allowed (%)	1.8	1.5	2.53
Disallowed (%)	0.00	0.00	0.00

Table S1. Cryo-EM data collection, refinement and validation statistics (continued).

	**Δ*pufX* monomer** (EMD-32062) (PDB 7VOY)	**Δ*pufY* monomer** (EMD-32042) (PDB 7VNM)	**Δ*pufY* dimer Type-1** (EMD-31835) (PDB 7VA9)	**Δ*pufY* dimer Type-2** (EMD-31875) (PDB 7VB9)
**Data collection and processing**				
Magnification	81,000 x	105,000 x	105,000 x	-
Voltage (kV)	300	300	300	-
Electron exposure (e^{-}/Å^2)	46.549	51.527	50.868	-
Defocus range (μm)	0.8-1.8	0.8-2.0	0.8-2.0	-
Pixel size (Å)	1.06	0.8285	0.8285	-
Symmetry imposed	C1	C1	C1	C1
Initial particle images (no.)	3,420,815	241,170	373,025	-
Final particle images (no.)	66,058	56,391	71,027	53,830
Map resolution (Å)	4.20	2.86	3.08	3.45
FSC threshold	0.143	0.143	0.143	0.143
Map resolution range (Å)	3.8-6.2	2.5-4.5	2.70-4.63	2.99-5.66
**Refinement**				
Initial model used (PDB code)	WT monomer	WT monomer	WT Class-1 dimer	Δ*pufY* RC−LH1 Type-1 dimer
Model resolution (Å)	3.55	2.43	2.62	3.08
FSC threshold	0.143	0.143	0.143	0.143
Map resolution range (Å)	-	-	-	-
Map sharpening *B* factor (Å^2)	-163.9	-94.3	-122.5	-122.7
Model composition				
Non-hydrogen atoms	22,119	20,589	42,176	36,469
Protein residues	2,479	2,112	4,350	3,807
Ligands	44	65	131	107
B factors (Å^2)				
Protein	77.32	25.32	23.27	33.61
Ligand	68.62	22.74	26.66	34.48
R.m.s. deviations				
Bond lengths (Å)	0.008	0.007	0.007	0.008
Bond angles (°)	0.929	1.006	1.069	0.912
**Validation**				
MolProbity score	2.10	1.57	2.09	2.23
Clashscore	15.97	10.03	15.42	15.18
Poor rotamers (%)	1.25	0.62	2.78	3.26
Ramachandran plot				
Favored (%)	95.38	97.81	97.70	97.11
Allowed (%)	4.58	2.19	2.30	2.86
Disallowed (%)	0.04	0.00	0.00	0.03

Table S2. Components of the WT RC−LH1 monomer and dimers from *Rba. sphaeroides.*

Proteins		Numbers			Cofactors	Numbers		
		Monomer	Dimer Class-1	Dimer Class-2		Monomer	Dimer Class-1	Dimer Class-2
LH1	α subunit	14	28	28	BChl *a*	28	56	56
	β subunit	14	28	28	Spheroidene (SPO)	25	50	50
RC	H	1	2	2	BChl *a*	4	8	8
	L	1	2	2	Spheroidene (SPO)	1	2	2
	M	1	2	2				
					BPhe	2	4	4
					Fe^{2+}	1	2	2
					UQ-10	4	6	8
					Cardiolipin (CDL)	2	4	4
					Phosphatidylcholine (PC1)	9	16	18
PufX		1	2	2				
PufY		1	2	2				
**Total**		**33**	**66**	**66**	**Total**	**76**	**148**	**152**

Table S3. Interactions within the RC−LH1 assembly.

RC-M:LH1

	Structure 1	Structure 2	Dist. [Å]	Interaction
1	M:ALA27[O]	9α:ARG15[NH2]	2.8	Hydrogen bond
2	M:SER54[OG]	8α:ARG15[NH1]	3.3	Hydrogen bond
3	M:ASN81[ND2]	11α:SER37[OG]	2.7	Hydrogen bond
4	M:TYR134[OH]	7α:ARG15[NH1]	3.4	Hydrogen bond

RC-L:LH1

	Structure 1	Structure 2	Dist. [Å]	Interaction
1	L:TRP25[O]	2α:ARG15[NH2]	3.0	Hydrogen bond
2	L:TRP25[O]	2α:ARG15[NE]	3.4	Hydrogen bond
3	L:TRP51[NE1]	2α:SER37[OG]	3.0	Hydrogen bond
4	L:TRP59[NE1]	3α:SER37[OG]	3.1	Hydrogen bond
5	L:ALA78[O]	1α:SER37[OG]	2.9	Hydrogen bond

RC-H:LH1

	Structure 1	Structure 2	Dist. [Å]	Interaction
1	H:ALA6[O]	6α:ASN42[ND2]	3.5	Hydrogen bond
2	H:GLY8[N]	5α:SER37[OG]	3.4	Hydrogen bond
3	H:GLY54[O]	4α:ARG15[NH1]	2.2	Hydrogen bond
4	H:PHE56[O]	4α:ARG15[NH1]	3.2	Hydrogen bond
5	H:SER93[OG]	2α:ARG15[NH1]	3.4	Hydrogen bond
6	H:GLU94[O]	2α:ARG15[NH1]	2.6	Hydrogen bond
7	H:ALA260[OXT]	1α:ARG14[NH2]	3.3	Hydrogen bond

LH1-1α:LH1-1β

	Structure 1	Structure 2	Dist. [Å]	Interaction
1	1α:TYR5[OH]	1β:ASP14[OD1]	3.2	Hydrogen bond
2	1α:TYR5[OH]	1β:ASP14[OD2]	3.1	Hydrogen bond
3	1α:TRP8[O]	1β:THR10[OG1]	3.0	Hydrogen bond
4	1α:MET9[O]	1β:TYR9[N]	3.0	Hydrogen bond
5	1α:GLN20[OE1]	1β:TYR24[OH]	2.3	Hydrogen bond
6	1α:GLN20[NE2]	1β:TYR24[OH]	3.2	Hydrogen bond
7	1α:TYR41[OH]	1β:ARG46[O]	3.0	Hydrogen bond
8	1α:TYR41[OH]	1β:PRO47[O]	2.9	Hydrogen bond

LH1-1α:LH1-2α

	Structure 1	Structure 2	Dist. [Å]	Interaction
1	1α:TYR41[OH]	2α:ARG53[NH1]	2.9	Hydrogen bond

Table S4. PufX interactions in the Class-1 RC−LH1 dimer. Note: no direct PufX-PufX interactions were identified.

	**Structure 1**	**Structure 2**	**Dist. [Å]**	**Interaction**
PufX:L	X:ARG49[NH2]	L:VAL137[O]	2.9	Hydrogen bond
	X:ARG49[NH2]	L:MET138[O]	3.2	Hydrogen bond
	X:ALA62[O]	L:TYR144[OH]	2.6	Hydrogen bond
	X:PRO65[O]	L:ASN159[ND2]	2.5	Hydrogen bond
	X:ASN66[OD1]	L:GLY143[O]	3.2	Hydrogen bond
	X:ASN66[ND2]	L:ALA145[O]	2.6	Hydrogen bond
PufX:H	X:LYS29[NZ]	H:TYR259[O]	2.3	Hydrogen bond
PufX:H (neighbouring monomer)	X:ASN14[OD1]	h:GLU258[O]	2.8	Hydrogen bond
	X:THR17[OG1]	h:GLU258[O]	2.4	Hydrogen bond
	X:ARG20[NH1]	h:TYR259[OH]	2.5	Hydrogen bond
	X:ARG20[NH2]	h:GLU258[OE2]	3.3	Salt bridge
PufX:LH1-1α (neighbouring monomer)	X:ASP9[O]	1α:ARG14[NH2]	3.6	Hydrogen bond
	X:ASP9[OD2]	1α:ARG14[NE]	3.9	Salt bridge
	X:ASP9[OD1]	1α:ARG14[NH2]	3.8	Salt bridge
PufX:LH1-1β (neighbouring monomer)	X:ILE6[O]	1β:GLN16[NE2]	2.8	Hydrogen bond
	X:ILE6[N]	1β:GLU19[OE1]	2.8	Hydrogen bond
	X:PHE7[O]	1β:GLN16[NE2]	3.1	Hydrogen bond
	X:PHE7[N]	1β:GLU19[OE1]	3.1	Hydrogen bond
	X:ARG53[NH2]	1β:ILE44[O]	2.9	Hydrogen bond
	X:ARG53[NH1]	1β:ILE44[O]	2.8	Hydrogen bond

Table S5. PufY interactions in the RC−LH1 monomer.

	Structure 1	Structure 2	Dist. [Å]	Interaction
PufY:LH1−14α	Y:Val4[O]	14α:Arg15[NH1]	3.4	Hydrogen bond
	Y:Val4[O]	14α:Arg15[NH2]	3.3	Hydrogen bond
	Y:Ser5[O]	14α:Arg15[NH2]	3.4	Hydrogen bond
	Y:Glu6[OE1]	14α:Arg15[NH2]	3.9	Salt bridge
PufY:LH1−13α	Y:Ala28[O]	13α:Ser37[OG]	3.3	Hydrogen bond
	Y:Thr49[O]	13α:Arg15[NH1]	2.7	Hydrogen bond
	Y:Pro50[O]	13α:Arg15[NH1]	3.0	Hydrogen bond
	Y:Asn51[OD1]	13α:Arg15[NE]	3.0	Hydrogen bond
PufY:RC-H	Y:Glu3[OE1]	H:Arg202[NH1]	3.9	Salt bridge

SUPPLEMENTARY REFERENCES

1 de Jong DH, Liguori N, van den Berg T, Arnarez C, Periole X, Marrink SJ. Atomistic and Coarse Grain Topologies for the Cofactors Associated with the Photosystem II Core Complex. *Journal of Physical Chemistry B* 2015; **119**:7791-7803.
2 Dahlberg M, Maliniak A. Mechanical Properties of Coarse-Grained Bilayers Formed by Cardiolipin and Zwitterionic Lipids. *J Chemical Theory and Computation* 2010; **6**:1638-1649.
3 Wassenaar TA, Ingolfsson HI, Bockmann RA, Tieleman DP, Marrink SJ. Computational Lipidomics with insane: A Versatile Tool for Generating Custom Membranes for Molecular Simulations. *Journal of Chemical Theory and Computation* 2015; **11**:2144-2155.
4 Jorgensen WL, Tiradorives J. The OPLS potential functions for proteins - energy minimizations for crystals of cyclic peptides and crambin. *Journal of the American Chemical Society* 1988; **110**:1657-1666.
5 Dodda LS, de Vaca IC, Tirado-Rives J, Jorgensen WL. LigParGen web server: an automatic OPLS-AA parameter generator for organic ligands. *Nucleic Acids Research* 2017; **45**:W331-W336.
6 Nose S. A molecular dynamics method for simulations in the canonical ensemble. *Molecular Physics* 1984; **52**:255-268.
7 Hoover WG. Canonical dynamics: Equilibrium phase-space distributions. *Physical Review A* 1985; **31**:1695-1697.
8 Parrinello M, Rahman A. Polymorphic transitions in single crystals: A new molecular dynamics method. *Journal of Applied Physics* 1981; **52**:7182-7190.
9 Tironi IG, Sperb R, Smith PE, Vangunsteren WF. A generalized reaction field method for molecular dynamics simulations. *Journal of Chemical Physics* 1995; **102**:5451-5459.
10 Hess B, Bekker H, Berendsen HJC, Fraaije J. LINCS: A linear constraint solver for molecular simulations. *Journal of Computational Chemistry* 1997; **18**:1463-1472.
11 Hess B. P-LINCS: A parallel linear constraint solver for molecular simulation. *Journal of Chemical Theory and Computation* 2008; **4**:116-122.
12 Bennett CH. Efficient estimation of free energy differences from Monte Carlo data. *Journal of Computational Physics* 1976; **22**:245-268.
13 Shirts MR, Bair E, Hooker G, Pande VS. Equilibrium free energies from nonequilibrium measurements using maximum-likelihood methods. *Physical Review Letters* 2003; **91**.
14 Cheng TJ, Zhao Y, Li X *et al.* Computation of octanol-water partition coefficients by guiding an additive model with knowledge. *J Chemical Information and Modeling* 2007; **47**:2140-2148.
15 Qian P, Croll TI, Hitchcock A *et al.* Cryo-EM structure of the dimeric *Rhodobacter sphaeroides* RC-LH1 core complex at 2.9 Å: the structural basis for dimerisation. *Biochem J* 2021; **478**:3923-3937.
