# Supplementary material for: Structural basis for the assembly and quinone transport mechanisms of the dimeric photosynthetic RC–LH1 supercomplex
Source: Nat Commun. 2022 Apr 13;13:1977. doi: 10.1038/s41467-022-29563-3 (PMC9007983; doi:10.1038/s41467-022-29563-3)
Supplement: Supplementary file 2 — Description of additional Supplementary File [file 41467_2022_29563_MOESM2_ESM.pdf]

Descriptions of additional Supplementary Information files

Movie S1. Comparison of the structures of the RC−LH1 Class-1 and Class-2 dimers. See also Fig. S10.

Movie S2. Coarse-grained molecular dynamics simulations of a quinone-free RC−LH1 Class-1 dimer illustrates that quinones can enter the RC−LH1 complex through the large opening of the S-shaped LH1 ring as the predominant quinone transport pathway. The full duration of the MD simulations was 5 μs. The progression of the quinone movement is presented every 100 ns. Entering of quinone into the complex occurred during 2600 and 4100 ns.

Movie S3. Coarse-grained molecular dynamics simulations of the *Rba. sphaeroides* RC−LH1 Class-1 dimer containing the quinones Q_A, Q_B, Q_3 and Q_Y (periplasmic view). The full duration of the MD simulations was 5 μs. The progression of the quinone movement is presented every 10 ns.

Movie S4. Coarse-grained molecular dynamics simulations of the *Rba. sphaeroides* RC−LH1 Class-1 dimer containing the quinones Q_A, Q_B, Q_3 and Q_Y (side view). The full duration of the MD simulations was 5 μs. The progression of the quinone movement is presented every 10 ns.

Data S1. Mass spectrometry data to identify the presence of PufY.

Data S2. Topologies and force-field parameters of TSCL in the itp format.

Data S3. Topologies and force-field parameters of SPO in the itp format.
