# Supplementary material for: Structural basis for the assembly and quinone transport mechanisms of the dimeric photosynthetic RC–LH1 supercomplex
Source: Nat Commun. 2022 Apr 13;13:1977. doi: 10.1038/s41467-022-29563-3 (PMC9007983; doi:10.1038/s41467-022-29563-3)

Accession	Description	Score	Coverage	# Proteins	# Unique Peptides	# Peptides	# PSMs	# AAs	MW [kDa]	calc. pI
Q3IZ15	ATP synthase subunit b 1 OS=Rhodobacter sphaeroides (strain ATCC 17023 / 2.4.1 / NCIB 8253 / DSM 158) OX=272943 GN=atpF1 PE=3 SV=2 - [ATPF1_RHOS4]	114.21	45.11	1	11	11	58	184	19.7	9.07
Q3J1A4	Light-harvesting protein B-875 alpha chain OS=Rhodobacter sphaeroides (strain ATCC 17023 / 2.4.1 / NCIB 8253 / DSM 158) OX=272943 GN=pufA PE=3 SV=1 - [LHA1_RHOS4]	88.37	39.66	1	6	6	101	58	6.8	9.70
Q3J4K5	Uncharacterized protein OS=Rhodobacter sphaeroides (strain ATCC 17023 / 2.4.1 / NCIB 8253 / DSM 158) OX=272943 GN=RSP_2125 PE=4 SV=1 - [Q3J4K5_RHOS4]	76.78	71.70	1	9	9	72	106	11.9	5.30
Q3J685	Light-harvesting complex, beta subunit OS=Rhodobacter sphaeroides (strain ATCC 17023 / 2.4.1 / NCIB 8253 / DSM 158) OX=272943 GN=puc2B PE=4 SV=1 - [Q3J685_RHOS4]	76.72	47.06	1	2	3	35	51	5.5	6.51
Q3J434	ATP synthase subunit delta OS=Rhodobacter sphaeroides (strain ATCC 17023 / 2.4.1 / NCIB 8253 / DSM 158) OX=272943 GN=atpH PE=3 SV=1 - [ATPD_RHOS4]	69.86	65.59	1	10	10	46	186	19.3	6.86
Q3J036	Uncharacterized protein OS=Rhodobacter sphaeroides (strain ATCC 17023 / 2.4.1 / NCIB 8253 / DSM 158) OX=272943 GN=RSP_6124 PE=4 SV=2 - [Q3J036_RHOS4]	63.54	48.45	1	4	4	33	97	10.8	4.88
Q3IZ14	ATP synthase subunit b 2 OS=Rhodobacter sphaeroides (strain ATCC 17023 / 2.4.1 / NCIB 8253 / DSM 158) OX=272943 GN=atpF2 PE=3 SV=1 - [ATPF2_RHOS4]	62.98	46.67	1	9	9	32	180	18.7	5.00
Q3J093	Uncharacterized protein OS=Rhodobacter sphaeroides (strain ATCC 17023 / 2.4.1 / NCIB 8253 / DSM 158) OX=272943 GN=RSP_0617 PE=4 SV=2 - [Q3J093_RHOS4]	55.67	77.78	1	8	10	30	90	10.1	6.55
Q3IY17	DNA-binding protein HU, form N OS=Rhodobacter sphaeroides (strain ATCC 17023 / 2.4.1 / NCIB 8253 / DSM 158) OX=272943 GN=RSP_1388 PE=3 SV=1 - [Q3IY17_RHOS4]	54.07	48.42	1	3	3	21	95	9.9	9.64
P13402	Intrinsic membrane protein PufX OS=Rhodobacter sphaeroides (strain ATCC 17023 / 2.4.1 / NCIB 8253 / DSM 158) OX=272943 GN=pufX PE=1 SV=1 - [PUFX_RHOS4]	40.44	25.61	1	2	2	32	82	9.0	9.54
Q3J338	Uncharacterized protein OS=Rhodobacter sphaeroides (strain ATCC 17023 / 2.4.1 / NCIB 8253 / DSM 158) OX=272943 GN=RSP_2641 PE=4 SV=1 - [Q3J338_RHOS4]	39.19	26.47	1	3	3	19	136	15.4	9.29
U5NRE4	Uncharacterized protein OS=Rhodobacter sphaeroides (strain ATCC 17023 / 2.4.1 / NCIB 8253 / DSM 158) OX=272943 GN=RSP_7517 PE=4 SV=1 - [U5NRE4_RHOS4]	36.93	47.73	1	2	2	12	44	4.8	7.96
Q3J5R4	50S ribosomal protein L29 OS=Rhodobacter sphaeroides (strain ATCC 17023 / 2.4.1 / NCIB 8253 / DSM 158) OX=272943 GN=rpmC PE=3 SV=1 - [Q3J5R4_RHOS4]	34.04	73.53	1	7	7	22	68	7.6	10.83
Q3J5J0	Sec translocon accessory complex subunit YajC OS=Rhodobacter sphaeroides (strain ATCC 17023 / 2.4.1 / NCIB 8253 / DSM 158) OX=272943 GN=YajC PE=3 SV=1 - [Q3J5J0_RHOS4]	31.74	42.59	1	4	4	23	108	11.5	9.63
U5NMI5	Uncharacterized protein OS=Rhodobacter sphaeroides (strain ATCC 17023 / 2.4.1 / NCIB 8253 / DSM 158) OX=272943 GN=RSP_7616 PE=4 SV=1 - [U5NMI5_RHOS4]	31.60	66.67	1	5	7	32	90	10.0	8.25
Q3J3E7	NADH-quinone oxidoreductase subunit K OS=Rhodobacter sphaeroides (strain ATCC 17023 / 2.4.1 / NCIB 8253 / DSM 158) OX=272943 GN=nuoK PE=3 SV=1 - [NUOK_RHOS4]	30.11	12.87	1	2	2	14	101	10.9	5.17
Q3J1A6	Reaction center protein M chain OS=Rhodobacter sphaeroides (strain ATCC 17023 / 2.4.1 / NCIB 8253 / DSM 158) OX=272943 GN=pufM PE=1 SV=3 - [RCEM_RHOS4]	29.26	15.91	1	5	5	27	308	34.5	6.64
U5NME9	Uncharacterized protein OS=Rhodobacter sphaeroides (strain ATCC 17023 / 2.4.1 / NCIB 8253 / DSM 158) OX=272943 GN=RSP_7571 PE=4 SV=1 - [U5NME9_RHOS4]	28.18	18.87	1	1	1	20	53	5.5	5.63
Q3J5M5	Uncharacterized protein OS=Rhodobacter sphaeroides (strain ATCC 17023 / 2.4.1 / NCIB 8253 / DSM 158) OX=272943 GN=RSP_1762 PE=4 SV=1 - [Q3J5M5_RHOS4]	28.10	29.83	1	7	7	18	238	26.9	9.47
Q3J145	Light-harvesting protein B-800/850 beta chain OS=Rhodobacter sphaeroides (strain ATCC 17023 / 2.4.1 / NCIB 8253 / DSM 158) OX=272943 GN=pucB PE=3 SV=3 - [LHB2_RHOS4]	23.94	58.82	1	3	4	16	51	5.4	5.67
Q3J1A5	Reaction center protein L chain OS=Rhodobacter sphaeroides (strain ATCC 17023 / 2.4.1 / NCIB 8253 / DSM 158) OX=272943 GN=pufL PE=1 SV=3 - [RCEL_RHOS4]	18.79	15.25	1	4	4	18	282	31.4	7.53
Q3IZ13	ATP synthase subunit c OS=Rhodobacter sphaeroides (strain ATCC 17023 / 2.4.1 / NCIB 8253 / DSM 158) OX=272943 GN=atpE PE=3 SV=1 - [ATPL_RHOS4]	18.59	12.82	1	1	1	12	78	7.7	4.89
Q3J045	ATP-dependent zinc metalloprotease FtsH OS=Rhodobacter sphaeroides (strain ATCC 17023 / 2.4.1 / NCIB 8253 / DSM 158) OX=272943 GN=ftsH PE=3 SV=1 - [Q3J045_RHOS4]	14.77	6.16	1	3	3	8	633	68.5	5.92
Q3J2Z2	Cytochrome b-c1 subunit IV OS=Rhodobacter sphaeroides (strain ATCC 17023 / 2.4.1 / NCIB 8253 / DSM 158) OX=272943 GN=fbcQ PE=4 SV=1 - [Q3J2Z2_RHOS4]	14.12	37.10	1	6	6	14	124	14.4	9.39
Q3J5M7	Uncharacterized protein OS=Rhodobacter sphaeroides (strain ATCC 17023 / 2.4.1 / NCIB 8253 / DSM 158) OX=272943 GN=RSP_1760 PE=4 SV=1 - [Q3J5M7_RHOS4]	13.81	21.10	1	4	4	9	218	24.9	10.10
Q3J433	ATP synthase subunit alpha OS=Rhodobacter sphaeroides (strain ATCC 17023 / 2.4.1 / NCIB 8253 / DSM 158) OX=272943 GN=atpA PE=3 SV=1 - [ATPA_RHOS4]	13.75	10.35	1	5	5	9	512	55.2	6.60
Q3IZ07	50S ribosomal protein L31 OS=Rhodobacter sphaeroides (strain ATCC 17023 / 2.4.1 / NCIB 8253 / DSM 158) OX=272943 GN=rpmE PE=3 SV=1 - [RL31_RHOS4]	13.35	45.21	1	2	2	8	73	8.1	9.09
Q3IW93	Putative integral membrane protein OS=Rhodobacter sphaeroides (strain ATCC 17023 / 2.4.1 / NCIB 8253 / DSM 158) OX=272943 GN=RSP_3588 PE=4 SV=2 - [Q3IW93_RHOS4]	12.21	22.22	1	4	4	9	126	14.2	10.61
Q3J2B5	Uncharacterized protein OS=Rhodobacter sphaeroides (strain ATCC 17023 / 2.4.1 / NCIB 8253 / DSM 158) OX=272943 GN=RSP_2908 PE=4 SV=1 - [Q3J2B5_RHOS4]	11.56	34.69	1	4	4	12	147	16.1	10.11
Q3J3D5	Sec-independent protein translocase protein TatB OS=Rhodobacter sphaeroides (strain ATCC 17023 / 2.4.1 / NCIB 8253 / DSM 158) OX=272943 GN=tatB PE=3 SV=1 - [TATB_RHOS4]	11.18	10.13	1	1	1	4	158	16.2	8.41
Q3J170	Reaction center protein H chain OS=Rhodobacter sphaeroides (strain ATCC 17023 / 2.4.1 / NCIB 8253 / DSM 158) OX=272943 GN=puhA PE=1 SV=1 - [RCEH_RHOS4]	11.08	38.85	1	7	7	13	260	28.0	6.30
Q3J5C1	Uncharacterized protein OS=Rhodobacter sphaeroides (strain ATCC 17023 / 2.4.1 / NCIB 8253 / DSM 158) OX=272943 GN=RSP_1864 PE=4 SV=1 - [Q3J5C1_RHOS4]	10.82	27.78	1	2	2	10	72	7.8	8.62
Q3J5S2	50S ribosomal protein L3 OS=Rhodobacter sphaeroides (strain ATCC 17023 / 2.4.1 / NCIB 8253 / DSM 158) OX=272943 GN=rplC PE=3 SV=1 - [RL3_RHOS4]	10.63	11.25	1	2	2	7	240	25.2	9.99
Q3J430	ATP synthase epsilon chain 1 OS=Rhodobacter sphaeroides (strain ATCC 17023 / 2.4.1 / NCIB 8253 / DSM 158) OX=272943 GN=atpC1 PE=3 SV=1 - [ATPE1_RHOS4]	10.50	18.94	1	3	3	14	132	13.8	4.64
Q3IZ77	Succinate dehydrogenase subunit C OS=Rhodobacter sphaeroides (strain ATCC 17023 / 2.4.1 / NCIB 8253 / DSM 158) OX=272943 GN=RSP_0974 PE=4 SV=1 - [Q3IZ77_RHOS4]	10.46	26.77	1	4	4	10	127	14.3	7.44
Q3J1Q0	NADH-quinone oxidoreductase subunit K OS=Rhodobacter sphaeroides (strain ATCC 17023 / 2.4.1 / NCIB 8253 / DSM 158) OX=272943 GN=nuoK PE=3 SV=1 - [Q3J1Q0_RHOS4]	10.39	14.71	1	2	2	4	102	10.6	6.51
Q3J5T6	Protein translocase subunit SecE OS=Rhodobacter sphaeroides (strain ATCC 17023 / 2.4.1 / NCIB 8253 / DSM 158) OX=272943 GN=secE PE=3 SV=1 - [Q3J5T6_RHOS4]	9.81	32.81	1	2	2	7	64	7.1	10.83
Q3J3D6	Sec-independent protein translocase protein TatA OS=Rhodobacter sphaeroides (strain ATCC 17023 / 2.4.1 / NCIB 8253 / DSM 158) OX=272943 GN=tatA PE=3 SV=1 - [Q3J3D6_RHOS4]	9.39	64.29	1	5	5	18	70	7.5	8.57
Q3J5T4	50S ribosomal protein L11 OS=Rhodobacter sphaeroides (strain ATCC 17023 / 2.4.1 / NCIB 8253 / DSM 158) OX=272943 GN=rplK PE=3 SV=1 - [RL11_RHOS4]	8.83	25.33	1	3	3	11	150	15.7	9.99
Q3J5F8	Uncharacterized protein OS=Rhodobacter sphaeroides (strain ATCC 17023 / 2.4.1 / NCIB 8253 / DSM 158) OX=272943 GN=RSP_6021 PE=4 SV=1 - [Q3J5F8_RHOS4]	8.78	34.48	1	1	1	5	58	6.2	11.41
Q3IZN3	Uncharacterized protein OS=Rhodobacter sphaeroides (strain ATCC 17023 / 2.4.1 / NCIB 8253 / DSM 158) OX=272943 GN=RSP_0822 PE=4 SV=1 - [Q3IZN3_RHOS4]	8.55	16.80	1	2	2	5	125	13.6	11.19
Q3J5M8	Uncharacterized protein OS=Rhodobacter sphaeroides (strain ATCC 17023 / 2.4.1 / NCIB 8253 / DSM 158) OX=272943 GN=RSP_1759 PE=4 SV=1 - [Q3J5M8_RHOS4]	8.54	35.96	1	3	3	15	89	10.3	7.42
Q9X4E2	Integration host factor subunit beta OS=Rhodobacter sphaeroides (strain ATCC 17023 / 2.4.1 / NCIB 8253 / DSM 158) OX=272943 GN=ihfB PE=3 SV=1 - [IHFB_RHOS4]	8.09	29.03	1	3	3	9	93	10.6	9.86
Q3IZG4	Nitrogen regulatory protein P-II family OS=Rhodobacter sphaeroides (strain ATCC 17023 / 2.4.1 / NCIB 8253 / DSM 158) OX=272943 GN=glnK PE=3 SV=1 - [Q3IZG4_RHOS4]	7.95	21.43	1	2	2	6	112	12.3	5.57
Q3J164	Cytochrome c2 OS=Rhodobacter sphaeroides (strain ATCC 17023 / 2.4.1 / NCIB 8253 / DSM 158) OX=272943 GN=cycA PE=1 SV=1 - [CYC2_RHOS4]	7.87	33.79	1	4	4	9	145	15.6	8.38
Q3J5A0	50S ribosomal protein L33 OS=Rhodobacter sphaeroides (strain ATCC 17023 / 2.4.1 / NCIB 8253 / DSM 158) OX=272943 GN=rpmG PE=3 SV=1 - [RL33_RHOS4]	7.53	25.45	1	1	1	4	55	6.4	10.20
Q3J660	Uncharacterized protein OS=Rhodobacter sphaeroides (strain ATCC 17023 / 2.4.1 / NCIB 8253 / DSM 158) OX=272943 GN=RSP_1581 PE=4 SV=1 - [Q3J660_RHOS4]	7.36	25.61	1	2	2	4	82	9.2	5.52
Q3J168	Putative photosynthetic complex assembly protein OS=Rhodobacter sphaeroides (strain ATCC 17023 / 2.4.1 / NCIB 8253 / DSM 158) OX=272943 GN=RSP_0293 PE=4 SV=1 - [Q3J168_RHOS4]	7.02	19.48	1	3	3	4	154	16.8	5.60
Q3IZ76	Succinate dehydrogenase subunit D OS=Rhodobacter sphaeroides (strain ATCC 17023 / 2.4.1 / NCIB 8253 / DSM 158) OX=272943 GN=RSP_0975 PE=4 SV=1 - [Q3IZ76_RHOS4]	6.92	10.57	1	1	1	4	123	13.6	9.38
Q3J497	Uncharacterized protein OS=Rhodobacter sphaeroides (strain ATCC 17023 / 2.4.1 / NCIB 8253 / DSM 158) OX=272943 GN=RSP_2231 PE=4 SV=1 - [Q3J497_RHOS4]	6.20	15.48	1	1	1	3	84	9.3	9.41
Q3J6D5	Putative HemY-like membrane protein OS=Rhodobacter sphaeroides (strain ATCC 17023 / 2.4.1 / NCIB 8253 / DSM 158) OX=272943 GN=RSP_1508 PE=4 SV=1 - [Q3J6D5_RHOS4]	5.96	5.19	1	2	2	3	482	52.4	6.89
Q3J0C1	Uncharacterized protein OS=Rhodobacter sphaeroides (strain ATCC 17023 / 2.4.1 / NCIB 8253 / DSM 158) OX=272943 GN=RSP_0589 PE=4 SV=1 - [Q3J0C1_RHOS4]	5.68	14.93	1	1	1	5	67	7.4	8.66
Q3J3E8	NADH-quinone oxidoreductase subunit J OS=Rhodobacter sphaeroides (strain ATCC 17023 / 2.4.1 / NCIB 8253 / DSM 158) OX=272943 GN=nuoJ PE=3 SV=1 - [Q3J3E8_RHOS4]	5.65	11.27	1	2	2	7	204	22.4	7.18
Q3J6I4	Peptidase inhibitor I78 family OS=Rhodobacter sphaeroides (strain ATCC 17023 / 2.4.1 / NCIB 8253 / DSM 158) OX=272943 GN=RSP_6001 PE=4 SV=1 - [Q3J6I4_RHOS4]	5.58	35.56	1	3	3	5	90	9.6	8.37
Q3J1Q9	NADH-quinone oxidoreductase subunit OS=Rhodobacter sphaeroides (strain ATCC 17023 / 2.4.1 / NCIB 8253 / DSM 158) OX=272943 GN=nuoA1 PE=3 SV=1 - [Q3J1Q9_RHOS4]	5.46	13.28	1	1	1	4	128	13.6	5.38
Q3J431	ATP synthase subunit beta 1 OS=Rhodobacter sphaeroides (strain ATCC 17023 / 2.4.1 / NCIB 8253 / DSM 158) OX=272943 GN=atpD1 PE=3 SV=1 - [ATPB1_RHOS4]	5.42	9.47	1	3	3	5	475	50.4	4.98
Q3IZW1	Acetyl-CoA acetyltransferase OS=Rhodobacter sphaeroides (strain ATCC 17023 / 2.4.1 / NCIB 8253 / DSM 158) OX=272943 GN=RSP_0745 PE=3 SV=2 - [Q3IZW1_RHOS4]	5.17	2.81	1	1	1	2	391	40.5	6.62
Q3J2H2	Multidrug/cation efflux pump, membrane fusion protein subunit OS=Rhodobacter sphaeroides (strain ATCC 17023 / 2.4.1 / NCIB 8253 / DSM 158) OX=272943 GN=RSP_2802 PE=3 SV=1 - [Q3J2H2_RHOS4]	4.87	2.81	1	1	1	2	391	41.7	4.60
Q3J5M3	50S ribosomal protein L20 OS=Rhodobacter sphaeroides (strain ATCC 17023 / 2.4.1 / NCIB 8253 / DSM 158) OX=272943 GN=rplT PE=3 SV=1 - [RL20_RHOS4]	4.84	17.50	1	1	1	2	120	13.5	11.02
Q3J1Z7	Putative conserved small protein OS=Rhodobacter sphaeroides (strain ATCC 17023 / 2.4.1 / NCIB 8253 / DSM 158) OX=272943 GN=RSP_0019 PE=4 SV=1 - [Q3J1Z7_RHOS4]	4.82	17.33	1	1	1	2	75	8.6	11.58
Q3J5Q4	50S ribosomal protein L30 OS=Rhodobacter sphaeroides (strain ATCC 17023 / 2.4.1 / NCIB 8253 / DSM 158) OX=272943 GN=rpmD PE=3 SV=1 - [RL30_RHOS4]	4.13	17.74	1	2	2	4	62	6.9	10.92
Q3IVL2	Uncharacterized protein OS=Rhodobacter sphaeroides (strain ATCC 17023 / 2.4.1 / NCIB 8253 / DSM 158) OX=272943 GN=RSP_1421 PE=4 SV=1 - [Q3IVL2_RHOS4]	3.62	10.77	1	1	1	2	65	7.3	10.78
Q3J0X8	Uncharacterized protein OS=Rhodobacter sphaeroides (strain ATCC 17023 / 2.4.1 / NCIB 8253 / DSM 158) OX=272943 GN=RSP_0381 PE=4 SV=1 - [Q3J0X8_RHOS4]	3.48	14.77	1	2	2	3	149	15.7	5.71
Q3IZ41	Uncharacterized protein OS=Rhodobacter sphaeroides (strain ATCC 17023 / 2.4.1 / NCIB 8253 / DSM 158) OX=272943 GN=RSP_1011 PE=4 SV=1 - [Q3IZ41_RHOS4]	3.31	14.95	1	1	1	1	107	12.2	4.88
Q3J3G1	NADH-quinone oxidoreductase subunit A OS=Rhodobacter sphaeroides (strain ATCC 17023 / 2.4.1 / NCIB 8253 / DSM 158) OX=272943 GN=nuoA PE=3 SV=1 - [Q3J3G1_RHOS4]	3.18	12.40	1	1	1	5	121	13.7	4.67
Q3J4G0	Biotin transporter OS=Rhodobacter sphaeroides (strain ATCC 17023 / 2.4.1 / NCIB 8253 / DSM 158) OX=272943 GN=RSP_2173 PE=3 SV=1 - [Q3J4G0_RHOS4]	2.53	9.94	1	2	2	5	181	18.2	11.49
Q3IWY8	Bacterioferritin OS=Rhodobacter sphaeroides (strain ATCC 17023 / 2.4.1 / NCIB 8253 / DSM 158) OX=272943 GN=bfr PE=3 SV=1 - [Q3IWY8_RHOS4]	2.44	7.88	1	1	1	1	165	18.8	4.83
Q3IYT8	30S ribosomal protein S15 OS=Rhodobacter sphaeroides (strain ATCC 17023 / 2.4.1 / NCIB 8253 / DSM 158) OX=272943 GN=rpsO PE=3 SV=1 - [RS15_RHOS4]	2.26	19.10	1	1	1	2	89	10.2	10.18
Q3IX22	Uncharacterized protein OS=Rhodobacter sphaeroides (strain ATCC 17023 / 2.4.1 / NCIB 8253 / DSM 158) OX=272943 GN=RSP_6236 PE=4 SV=2 - [Q3IX22_RHOS4]	2.19	15.00	1	1	1	3	80	8.8	8.73
Q3J366	Integration host factor subunit alpha OS=Rhodobacter sphaeroides (strain ATCC 17023 / 2.4.1 / NCIB 8253 / DSM 158) OX=272943 GN=ihfA PE=3 SV=1 - [IHFA_RHOS4]	2.18	19.00	1	2	2	2	100	11.0	9.98
Q3IZZ0	30S ribosomal protein S21 OS=Rhodobacter sphaeroides (strain ATCC 17023 / 2.4.1 / NCIB 8253 / DSM 158) OX=272943 GN=rpsU PE=3 SV=1 - [RS21_RHOS4]	2.14	13.24	1	1	1	2	68	8.0	11.43
Q3J5S4	Elongation factor Tu OS=Rhodobacter sphaeroides (strain ATCC 17023 / 2.4.1 / NCIB 8253 / DSM 158) OX=272943 GN=tuf1 PE=3 SV=1 - [EFTU_RHOS4]	2.12	3.07	1	1	1	1	391	42.8	5.44
Q3IXR3	SH3 domain-containing protein OS=Rhodobacter sphaeroides (strain ATCC 17023 / 2.4.1 / NCIB 8253 / DSM 158) OX=272943 GN=RSP_6161 PE=4 SV=1 - [Q3IXR3_RHOS4]	2.00	21.28	1	2	2	3	94	9.9	8.76
Q3IZG2	Putative yrbC OS=Rhodobacter sphaeroides (strain ATCC 17023 / 2.4.1 / NCIB 8253 / DSM 158) OX=272943 GN=RSP_0890 PE=4 SV=1 - [Q3IZG2_RHOS4]	1.92	5.80	1	1	1	1	207	22.8	10.18
Q3IZD1	Outer membrane transport energization protein ExbD OS=Rhodobacter sphaeroides (strain ATCC 17023 / 2.4.1 / NCIB 8253 / DSM 158) OX=272943 GN=exbD PE=3 SV=1 - [Q3IZD1_RHOS4]	1.90	10.64	1	1	1	1	141	15.0	4.60
Q3J2Z4	Cytochrome c-type biogenesis protein, cycH OS=Rhodobacter sphaeroides (strain ATCC 17023 / 2.4.1 / NCIB 8253 / DSM 158) OX=272943 GN=RSP_2685 PE=4 SV=2 - [Q3J2Z4_RHOS4]	1.86	3.29	1	1	1	1	426	45.2	4.89
Q3J4Q5	Transcriptional regulator OS=Rhodobacter sphaeroides (strain ATCC 17023 / 2.4.1 / NCIB 8253 / DSM 158) OX=272943 GN=RSP_2075 PE=4 SV=1 - [Q3J4Q5_RHOS4]	1.85	6.90	1	1	1	2	116	12.9	9.33
Q3J4K2	Phosphoribosylformylglycinamidine synthase subunit PurQ OS=Rhodobacter sphaeroides (strain ATCC 17023 / 2.4.1 / NCIB 8253 / DSM 158) OX=272943 GN=purQ PE=3 SV=1 - [PURQ_RHOS4]	1.82	7.21	1	1	1	1	222	23.4	6.39
Q3J5B9	Transcriptional regulator, AsnC family OS=Rhodobacter sphaeroides (strain ATCC 17023 / 2.4.1 / NCIB 8253 / DSM 158) OX=272943 GN=RSP_1866 PE=4 SV=1 - [Q3J5B9_RHOS4]	1.78	8.61	1	1	1	1	151	17.2	6.80
Q3J144	Light-harvesting protein B-800/850 alpha chain OS=Rhodobacter sphaeroides (strain ATCC 17023 / 2.4.1 / NCIB 8253 / DSM 158) OX=272943 GN=pucA PE=3 SV=1 - [LHA2_RHOS4]	1.74	11.11	1	1	1	5	54	5.6	8.68
Q3J5Q3	50S ribosomal protein L15 OS=Rhodobacter sphaeroides (strain ATCC 17023 / 2.4.1 / NCIB 8253 / DSM 158) OX=272943 GN=rplO PE=3 SV=1 - [RL15_RHOS4]	1.73	20.50	1	2	2	2	161	16.7	10.37
Q3J3B5	Putative succinoglycan biosynthesis transport protein ExoP OS=Rhodobacter sphaeroides (strain ATCC 17023 / 2.4.1 / NCIB 8253 / DSM 158) OX=272943 GN=exoP PE=4 SV=2 - [Q3J3B5_RHOS4]	1.72	2.28	1	1	1	3	789	85.7	6.98
Q3J0X3	Cold-shock DNA-binding protein family OS=Rhodobacter sphaeroides (strain ATCC 17023 / 2.4.1 / NCIB 8253 / DSM 158) OX=272943 GN=RSP_0386 PE=4 SV=1 - [Q3J0X3_RHOS4]	1.71	13.24	1	1	1	5	68	7.3	8.57
Q3J0S4	Yip1 domain-containing protein OS=Rhodobacter sphaeroides (strain ATCC 17023 / 2.4.1 / NCIB 8253 / DSM 158) OX=272943 GN=RSP_0433 PE=4 SV=1 - [Q3J0S4_RHOS4]	1.69	8.18	1	1	1	1	159	17.1	8.57
Q3J509	Uncharacterized protein OS=Rhodobacter sphaeroides (strain ATCC 17023 / 2.4.1 / NCIB 8253 / DSM 158) OX=272943 GN=RSP_6027 PE=4 SV=1 - [Q3J509_RHOS4]	1.67	11.01	1	1	1	2	109	11.7	11.44
Q3J1S0	Haloacid dehalogenase superfamily protein, subfamily IA, variant 3 with third motif having DD or ED OS=Rhodobacter sphaeroides (strain ATCC 17023 / 2.4.1 / NCIB 8253 / DSM 158) OX=272943 GN=RSP_0089 PE=4 SV=1 - [Q3J1S0_RHOS4]	1.65	4.39	1	1	1	1	228	24.6	5.91
Q3J0L7	Hydrogenase protein large subunit OS=Rhodobacter sphaeroides (strain ATCC 17023 / 2.4.1 / NCIB 8253 / DSM 158) OX=272943 GN=hupL PE=3 SV=1 - [Q3J0L7_RHOS4]	1.65	2.68	1	1	1	1	596	66.3	6.39
Q3J258	Uncharacterized protein OS=Rhodobacter sphaeroides (strain ATCC 17023 / 2.4.1 / NCIB 8253 / DSM 158) OX=272943 GN=RSP_2964 PE=4 SV=2 - [Q3J258_RHOS4]	1.64	11.94	1	1	1	1	67	7.2	9.11
U5NMT9	Uncharacterized protein OS=Rhodobacter sphaeroides (strain ATCC 17023 / 2.4.1 / NCIB 8253 / DSM 158) OX=272943 GN=RSP_7537 PE=4 SV=1 - [U5NMT9_RHOS4]	1.60	15.25	1	1	1	2	59	6.1	12.60
Q3J2Z1	Putative sarcosine oxidase delta subunit protein OS=Rhodobacter sphaeroides (strain ATCC 17023 / 2.4.1 / NCIB 8253 / DSM 158) OX=272943 GN=soxD PE=4 SV=1 - [Q3J2Z1_RHOS4]	0.00	7.48	1	1	1	1	107	12.2	7.37
Q3IZ12	ATP synthase subunit a OS=Rhodobacter sphaeroides (strain ATCC 17023 / 2.4.1 / NCIB 8253 / DSM 158) OX=272943 GN=atpB PE=3 SV=2 - [ATP6_RHOS4]	0.00	3.07	1	1	1	2	261	28.5	7.33
Q3J5S5	Elongation factor G OS=Rhodobacter sphaeroides (strain ATCC 17023 / 2.4.1 / NCIB 8253 / DSM 158) OX=272943 GN=fusA PE=3 SV=1 - [EFG_RHOS4]	0.00	1.13	1	1	1	1	705	77.7	5.14
Q53228	Photosynthetic apparatus regulatory protein RegA OS=Rhodobacter sphaeroides (strain ATCC 17023 / 2.4.1 / NCIB 8253 / DSM 158) OX=272943 GN=regA PE=1 SV=2 - [REGA_RHOS4]	0.00	8.70	1	1	1	1	184	20.5	7.39
Q3IYZ1	50S ribosomal protein L34 OS=Rhodobacter sphaeroides (strain ATCC 17023 / 2.4.1 / NCIB 8253 / DSM 158) OX=272943 GN=rpmH PE=3 SV=1 - [RL34_RHOS4]	0.00	20.45	1	1	1	3	44	5.0	12.95
Q3J5R1	50S ribosomal protein L24 OS=Rhodobacter sphaeroides (strain ATCC 17023 / 2.4.1 / NCIB 8253 / DSM 158) OX=272943 GN=rplX PE=3 SV=1 - [RL24_RHOS4]	0.00	10.89	1	1	1	1	101	10.8	10.17
Q3J5Q7	50S ribosomal protein L6 OS=Rhodobacter sphaeroides (strain ATCC 17023 / 2.4.1 / NCIB 8253 / DSM 158) OX=272943 GN=rplF PE=3 SV=1 - [RL6_RHOS4]	0.00	7.91	1	1	1	1	177	19.4	9.99
Q3J2H4	Queuine tRNA-ribosyltransferase OS=Rhodobacter sphaeroides (strain ATCC 17023 / 2.4.1 / NCIB 8253 / DSM 158) OX=272943 GN=tgt PE=3 SV=1 - [TGT_RHOS4]	0.00	2.39	1	1	1	1	376	41.5	7.14
Q3IWE4	Hemolysin-type calcium-binding region, RTX OS=Rhodobacter sphaeroides (strain ATCC 17023 / 2.4.1 / NCIB 8253 / DSM 158) OX=272943 GN=RSP_3539 PE=4 SV=2 - [Q3IWE4_RHOS4]	0.00	0.46	1	1	1	1	2629	265.3	3.88
Q3J336	Bifunctional glutamine synthetase adenylyltransferase/adenylyl-removing enzyme OS=Rhodobacter sphaeroides (strain ATCC 17023 / 2.4.1 / NCIB 8253 / DSM 158) OX=272943 GN=glnE PE=3 SV=2 - [Q3J336_RHOS4]	0.00	1.81	1	1	1	1	941	102.2	6.16
Q3IZ74	Uncharacterized protein OS=Rhodobacter sphaeroides (strain ATCC 17023 / 2.4.1 / NCIB 8253 / DSM 158) OX=272943 GN=RSP_0977 PE=4 SV=1 - [Q3IZ74_RHOS4]	0.00	18.87	1	1	1	2	106	10.9	8.37
Q3IZW6	Aa3-type cytochrome c oxidase subunit IV OS=Rhodobacter sphaeroides (strain ATCC 17023 / 2.4.1 / NCIB 8253 / DSM 158) OX=272943 GN=RSP_6128 PE=4 SV=1 - [Q3IZW6_RHOS4]	0.00	55.74	1	2	2	2	61	6.4	6.93
Q3IYB1	Putative Aldehyde dehydrogenase OS=Rhodobacter sphaeroides (strain ATCC 17023 / 2.4.1 / NCIB 8253 / DSM 158) OX=272943 GN=RSP_1292 PE=3 SV=1 - [Q3IYB1_RHOS4]	0.00	2.23	1	1	1	1	494	51.7	6.10
Q3HKN0	Uncharacterized protein OS=Rhodobacter sphaeroides (strain ATCC 17023 / 2.4.1 / NCIB 8253 / DSM 158) OX=272943 GN=RSP_3856 PE=1 SV=1 - [Q3HKN0_RHOS4]	0.00	5.66	1	1	1	1	212	22.3	9.06
Q3IVD1	3-oxoadipate enol-lactonase OS=Rhodobacter sphaeroides (strain ATCC 17023 / 2.4.1 / NCIB 8253 / DSM 158) OX=272943 GN=pcaD PE=4 SV=1 - [Q3IVD1_RHOS4]	0.00	8.65	1	1	1	1	266	29.0	5.92
Q3J395	Amino acid/amide ABC transporter substrate-binding protein, HAAT family OS=Rhodobacter sphaeroides (strain ATCC 17023 / 2.4.1 / NCIB 8253 / DSM 158) OX=272943 GN=RSP_2582 PE=4 SV=2 - [Q3J395_RHOS4]	0.00	3.55	1	1	1	1	394	43.0	5.17
Q3IWF1	AMP-binding enzyme OS=Rhodobacter sphaeroides (strain ATCC 17023 / 2.4.1 / NCIB 8253 / DSM 158) OX=272943 GN=RSP_3532 PE=4 SV=1 - [Q3IWF1_RHOS4]	0.00	1.26	1	1	1	1	554	60.5	5.53
U5NMT8	Uncharacterized protein OS=Rhodobacter sphaeroides (strain ATCC 17023 / 2.4.1 / NCIB 8253 / DSM 158) OX=272943 GN=RSP_7581 PE=4 SV=1 - [U5NMT8_RHOS4]	0.00	12.24	1	1	1	1	49	5.6	6.60
Q3J099	Transcriptional regulator, HxlR family OS=Rhodobacter sphaeroides (strain ATCC 17023 / 2.4.1 / NCIB 8253 / DSM 158) OX=272943 GN=RSP_0611 PE=4 SV=1 - [Q3J099_RHOS4]	0.00	11.02	1	1	1	1	127	14.5	8.51
Q3IWQ2	Uncharacterized protein OS=Rhodobacter sphaeroides (strain ATCC 17023 / 2.4.1 / NCIB 8253 / DSM 158) OX=272943 GN=RSP_3428 PE=4 SV=1 - [Q3IWQ2_RHOS4]	0.00	2.15	1	1	1	1	652	69.8	7.01
Q3J6C4	Histone-like protein of HNS family OS=Rhodobacter sphaeroides (strain ATCC 17023 / 2.4.1 / NCIB 8253 / DSM 158) OX=272943 GN=spbA PE=4 SV=1 - [Q3J6C4_RHOS4]	0.00	13.46	1	1	1	1	104	11.5	9.31
Q3IZ55	Multisubunit potassium/proton antiporter, PhaF subunit OS=Rhodobacter sphaeroides (strain ATCC 17023 / 2.4.1 / NCIB 8253 / DSM 158) OX=272943 GN=phaF PE=4 SV=1 - [Q3IZ55_RHOS4]	0.00	11.24	1	1	1	4	89	9.5	8.19
Q3J1V2	FliK, flagellar hook-length control protein OS=Rhodobacter sphaeroides (strain ATCC 17023 / 2.4.1 / NCIB 8253 / DSM 158) OX=272943 GN=fliK PE=4 SV=1 - [Q3J1V2_RHOS4]	0.00	2.58	1	1	1	1	699	66.8	4.67
Q3J1Q6	NADH dehydrogenase subunit OS=Rhodobacter sphaeroides (strain ATCC 17023 / 2.4.1 / NCIB 8253 / DSM 158) OX=272943 GN=nuoE1 PE=4 SV=1 - [Q3J1Q6_RHOS4]	0.00	6.37	1	1	1	1	157	16.7	6.01
Q3IXZ7	Chaperone protein ClpB OS=Rhodobacter sphaeroides (strain ATCC 17023 / 2.4.1 / NCIB 8253 / DSM 158) OX=272943 GN=clpB PE=3 SV=1 - [Q3IXZ7_RHOS4]	0.00	1.26	1	1	1	1	870	95.6	5.53
Q3J3F7	NADH dehydrogenase subunit E OS=Rhodobacter sphaeroides (strain ATCC 17023 / 2.4.1 / NCIB 8253 / DSM 158) OX=272943 GN=nuoE PE=4 SV=1 - [Q3J3F7_RHOS4]	0.00	3.96	1	1	1	1	303	32.3	5.11
Q3IXI0	Gluconolactonase OS=Rhodobacter sphaeroides (strain ATCC 17023 / 2.4.1 / NCIB 8253 / DSM 158) OX=272943 GN=RSP_3138 PE=4 SV=1 - [Q3IXI0_RHOS4]	0.00	3.23	1	1	1	1	279	30.7	5.08

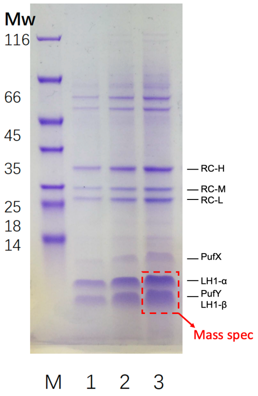

Supplement: Supplementary file 7 — Supplementary Data S1 [file 41467_2022_29563_MOESM7_ESM.pdf]
