# Supplementary material for: Structural basis for the assembly and quinone transport mechanisms of the dimeric photosynthetic RC–LH1 supercomplex
Source: Nat Commun. 2022 Apr 13;13:1977. doi: 10.1038/s41467-022-29563-3 (PMC9007983; doi:10.1038/s41467-022-29563-3)
Supplement: Supplementary file 8 — Supplementary Data S2 [file 41467_2022_29563_MOESM8_ESM.docx]

**Data S2. Topologies and force-field parameters of TSCL in the itp format.**

#define mb_pg1 0.370 5500.0 ; phosphatidyl/linking glycerol

#define mb_pg2 0.470 1250.0 ; phosphatidyl/glycerol

#define mb_gg 0.370 1250.0 ; glycerol linker

#define mb_cc 0.470 1250.0 ; tail

#define ma_pgp 105.000 45.0 ; linking glycerol

#define ma_gpg 110.000 25.0 ; linking glycerol/phosphatidyl/glycerol

#define ma_pgg 120.000 25.0 ; phosphatidyl/glycerol

#define ma_pgc 180.000 25.0 ; phosphatidyl/glycerol/tail

#define ma_gcc 180.000 25.0 ; glycerol/tail

#define ma_ccc 180.000 25.0 ; saturated tail

[ moleculetype ]

; molname nrexcl

CDL2 1

[ atoms ]

; i type resnr residue atom cgnr charge

1 Nda 1 CDL2 GL0 1 0.0

2 Qa 1 CDL2 PO41 2 -1.0

3 Na 1 CDL2 GL11 3 0.0

4 Na 1 CDL2 GL21 4 0.0

5 C1 1 CDL2 C1A1 5 0.0

6 C1 1 CDL2 C2A1 6 0.0

7 C1 1 CDL2 C3A1 7 0.0

8 C1 1 CDL2 C4A1 8 0.0

9 C1 1 CDL2 C1B1 10 0.0

10 C1 1 CDL2 C2B1 11 0.0

11 C1 1 CDL2 C3B1 12 0.0

12 C1 1 CDL2 C4B1 13 0.0

13 Qa 1 CDL2 PO42 15 -1.0

14 Na 1 CDL2 GL21 16 0.0

15 Na 1 CDL2 GL22 17 0.0

16 C1 1 CDL2 C1A2 18 0.0

17 C1 1 CDL2 C2A2 19 0.0

18 C1 1 CDL2 C3A2 20 0.0

19 C1 1 CDL2 C4A2 21 0.0

20 C1 1 CDL2 C1B2 23 0.0

21 C1 1 CDL2 C2B2 24 0.0

22 C1 1 CDL2 C3B2 25 0.0

23 C1 1 CDL2 C4B2 26 0.0

[ bonds ]

; i j funct alias

1 2 1 mb_pg1

1 15 1 mb_pg1

2 3 1 mb_pg2

3 4 1 mb_gg

3 5 1 mb_cc

5 6 1 mb_cc

6 7 1 mb_cc

7 8 1 mb_cc

4 9 1 mb_cc

9 10 1 mb_cc

10 11 1 mb_cc

11 12 1 mb_cc

13 14 1 mb_pg2

14 15 1 mb_gg

14 16 1 mb_cc

16 17 1 mb_cc

17 18 1 mb_cc

18 19 1 mb_cc

15 20 1 mb_cc

20 21 1 mb_cc

21 22 1 mb_cc

22 23 1 mb_cc

[ angles ]

; i j k funct alias

2 1 15 2 ma_pgp

1 2 3 2 ma_gpg

1 15 16 2 ma_gpg

2 3 4 2 ma_pgg

2 3 5 2 ma_pgc

3 5 6 2 ma_gcc

5 6 7 2 ma_ccc

6 7 8 2 ma_ccc

4 9 10 2 ma_gcc

9 10 11 2 ma_ccc

10 11 12 2 ma_ccc

13 14 15 2 ma_pgg

13 14 16 2 ma_pgc

14 16 17 2 ma_gcc

16 17 18 2 ma_ccc

17 18 19 2 ma_ccc

15 20 21 2 ma_gcc

20 21 22 2 ma_ccc

21 22 23 2 ma_ccc
