# Supplementary material for: Structural basis for the assembly and quinone transport mechanisms of the dimeric photosynthetic RC–LH1 supercomplex
Source: Nat Commun. 2022 Apr 13;13:1977. doi: 10.1038/s41467-022-29563-3 (PMC9007983; doi:10.1038/s41467-022-29563-3)
Supplement: Supplementary file 9 — Supplementary Data S3 [file 41467_2022_29563_MOESM9_ESM.docx]

**Data S3. Topologies and force-field parameters of SPO in the itp format.**

[moleculetype]

; molname nrexcl

SPO 1

[ atoms ]

; id type resnr residu atom cgnr charge

1 N0 1 SPO B1 1 0.0

2 C3 1 SPO B2 2 0.0

3 C4 1 SPO B3 3 0.0

4 C4 1 SPO B4 4 0.0

5 C4 1 SPO B5 5 0.0

6 C4 1 SPO B6 6 0.0

7 C4 1 SPO B7 7 0.0

8 C3 1 SPO B8 8 0.0

9 C3 1 SPO B9 9 0.0

[ bonds ]

; i j funct length force.c.

7 8 1 0.470 7000

8 9 1 0.470 7000

[ constraints ]

; i j funct length

1 2 1 0.240

2 3 1 0.340

3 4 1 0.450

4 5 1 0.480

5 6 1 0.500

6 7 1 0.490

[ angles ]

; i j k funct angle force.c.

1 2 3 2 159.0 6000

2 3 4 2 154.0 2000

3 4 5 2 165.0 15000

4 5 6 2 159.0 6000

5 6 7 2 159.0 6000

6 7 8 2 135.0 40

7 8 9 2 155.0 15

[ dihedrals ]

; i j k l funct k a0 a1 a2 a3 a4

2 3 4 5 11 5000 1 -1 0 0 0

3 4 5 6 11 5000 1 -1 0 0 0

4 5 6 7 11 900 -1 1 0 0 0

5 6 7 8 11 300 1 -1 0 0 0
